# Supplementary material for: Design, Synthesis, and Antioxidant and Anti-Tyrosinase Activities of (Z)-5-Benzylidene-2-(naphthalen-1-ylamino)thiazol-4(5H)-one Analogs: In Vitro and In Vivo Insights
Source: Molecules. 2025 Jan 13;30(2):289. doi: 10.3390/molecules30020289 (PMC11767423; doi:10.3390/molecules30020289)
Supplement: Supplementary file 1 [file molecules-30-00289-s001.zip › molecules-3368690-supplementary.pdf]

Supporting Information

For

Design, synthesis, and antioxidant and anti-tyrosinase activities of (*Z*)-5-benzylidene-2-(naphthalen-1-ylamino)thiazol-4(5*H*)-one analogs: In vitro and in vivo insights

Hee Jin Jung [1,†], Hye Jin Kim [1,†], Hyeon Seo Park [1], Hye Soo Park [1], Jeongin Ko [1], Dahye Yoon [1], Yujin Park [2], Pusoon Chun [3], Hae Young Chung [4], Hyung Ryong Moon [1,*]

[1] Department of Manufacturing Pharmacy, College of Pharmacy and Research Institute for Drug Development, Pusan National University, Busan 46241, Republic of Korea

[2] Department of Medicinal Chemistry, New Drug Development Center, Daegu-Gyeongbuk Medical Innovation Foundation, Daegu 41061, Republic of Korea

[3] College of Pharmacy and Inje Institute of Pharmaceutical Sciences and Research, Inje University, Gimhae 50834, Republic of Korea

[4] Department of Pharmacy, College of Pharmacy and Research Institute for Drug Development, Pusan National University, Busan 46241, Republic of Korea

Contents

Figure S1. ^{1}H NMR spectrum of analog **1** 5

Figure S2. ^{13}C NMR spectrum of analog **1** 6

Figure S3. LRMS (ESI+) spectrum of analog **1** 7

Figure S4. LRMS (ESI–) spectrum of analog **1** 8

Figure S5. ^{1}H NMR spectrum of analog **2** 9

Figure S6. ^{13}C NMR spectrum of analog **2** 10

Figure S7. LRMS (ESI–) spectrum of analog **2** 11

Figure S8. ^{1}H NMR spectrum of analog **3** 12

Figure S9. ^{13}C NMR spectrum of analog **3** 13

Figure S10. LRMS (ESI–) spectrum of analog **3** 14

Figure S11. ^{1}H NMR spectrum of analog **4** 15

Figure S12. ^{13}C NMR spectrum of analog **4** 16

Figure S13-1. HRMS (ESI+) spectrum of analog **4** 17

Figure S13-2. HRMS (ESI+) spectrum of analog **4** 18

Figure S13-3. HRMS (ESI+) spectrum of analog **4** 19

Figure S14. ^{1}H NMR spectrum of analog **5** 20

Figure S15. ^{13}C NMR spectrum of analog **5** 21

Figure S16. LRMS (ESI–) spectrum of analog **5** 22

Figure S17. ^{1}H NMR spectrum of analog **6** 23

Figure S18. ^{13}C NMR spectrum of analog **6** 24

Figure S19. LRMS (ESI–) spectrum of analog **6** 25

Figure S20. ^{1}H NMR spectrum of analog **7** 26

Figure S21. ^{13}C NMR spectrum of analog **7** 27

Figure S22. LRMS (ESI–) spectrum of analog **7** ... 28
Figure S23. ^{1}H NMR spectrum of analog **8** ... 29
Figure S24. ^{13}C NMR spectrum of analog **8** ... 30
Figure S25. LRMS (ESI+) spectrum of analog **8** ... 31
Figure S26. LRMS (ESI+) spectrum of analog **8** ... 32
Figure S27. ^{1}H NMR spectrum of analog **9** ... 33
Figure S28. ^{13}C NMR spectrum of analog **9** ... 34
Figure S29. LRMS (ESI+) spectrum of analog **9** ... 35
Figure S30. LRMS (ESI–) spectrum of analog **9** ... 36
Figure S31. ^{1}H NMR spectrum of analog **10** ... 37
Figure S32. ^{13}C NMR spectrum of analog **10** ... 38
Figure S33. LRMS (ESI+) spectrum of analog **10** ... 39
Figure S34-1. HRMS (ESI+) spectrum of analog **10** ... 40
Figure S34-2. HRMS (ESI+) spectrum of analog **10** ... 41
Figure S34-3. HRMS (ESI+) spectrum of analog **10** ... 42
Figure S35. ^{1}H NMR spectrum of analog **11** ... 43
Figure S36. ^{13}C NMR spectrum of analog **11** ... 44
Figure S37. LRMS (ESI+) spectrum of analog **11** ... 45
Figure S38. LRMS (ESI–) spectrum of analog **11** ... 46
Figure S39. ^{1}H NMR spectrum of analog **12** ... 47
Figure S40. ^{13}C NMR spectrum of analog **12** ... 48
Figure S41. LRMS (ESI–) spectrum of analog **12** ... 49
Figure S42. ^{1}H NMR spectrum of analog **13** ... 50
Figure S43. ^{13}C NMR spectrum of analog **13** ... 51

Figure S44. LRMS (ESI+) spectrum of analog **13** 52

Figure S45. LRMS (ESI–) spectrum of analog **13** 53

Figure S46. ^{1}H NMR spectrum of analog **14** 54

Figure S47. ^{13}C NMR spectrum of analog **14** 55

Figure S48. LRMS (ESI+) spectrum of analog **14** 56

Figure S49. LRMS (ESI–) spectrum of analog **14** 57

Figure S50. ^{1}H NMR spectrum of analog **15** 58

Figure S51. ^{13}C NMR spectrum of analog **15** 59

Figure S52. LRMS (ESI–) spectrum of analog **15** 60

Figure S53-1. HRMS (ESI+) spectrum of analog **15** 61

Figure S53-2. HRMS (ESI+) spectrum of analog **15** 62

Figure S53-3. HRMS (ESI+) spectrum of analog **15** 63

Figure S54. ^{1}H NMR spectrum of analog **16** 64

Figure S55. ^{1}H NMR spectrum of analog **17** 65

Figure S56. ^{13}C NMR spectrum of analog **17** 66

Figure S57. LRMS (ESI+) spectrum of analog **17** 67

Figure S58. LRMS (ESI+) spectrum of analog **17** 68

Figure S59. In silico 2D results obtained from docking simulation of ligands (**1**–**9** and **11**–**15**) with mushroom tyrosinase (PDB ID: 2Y9X) 69

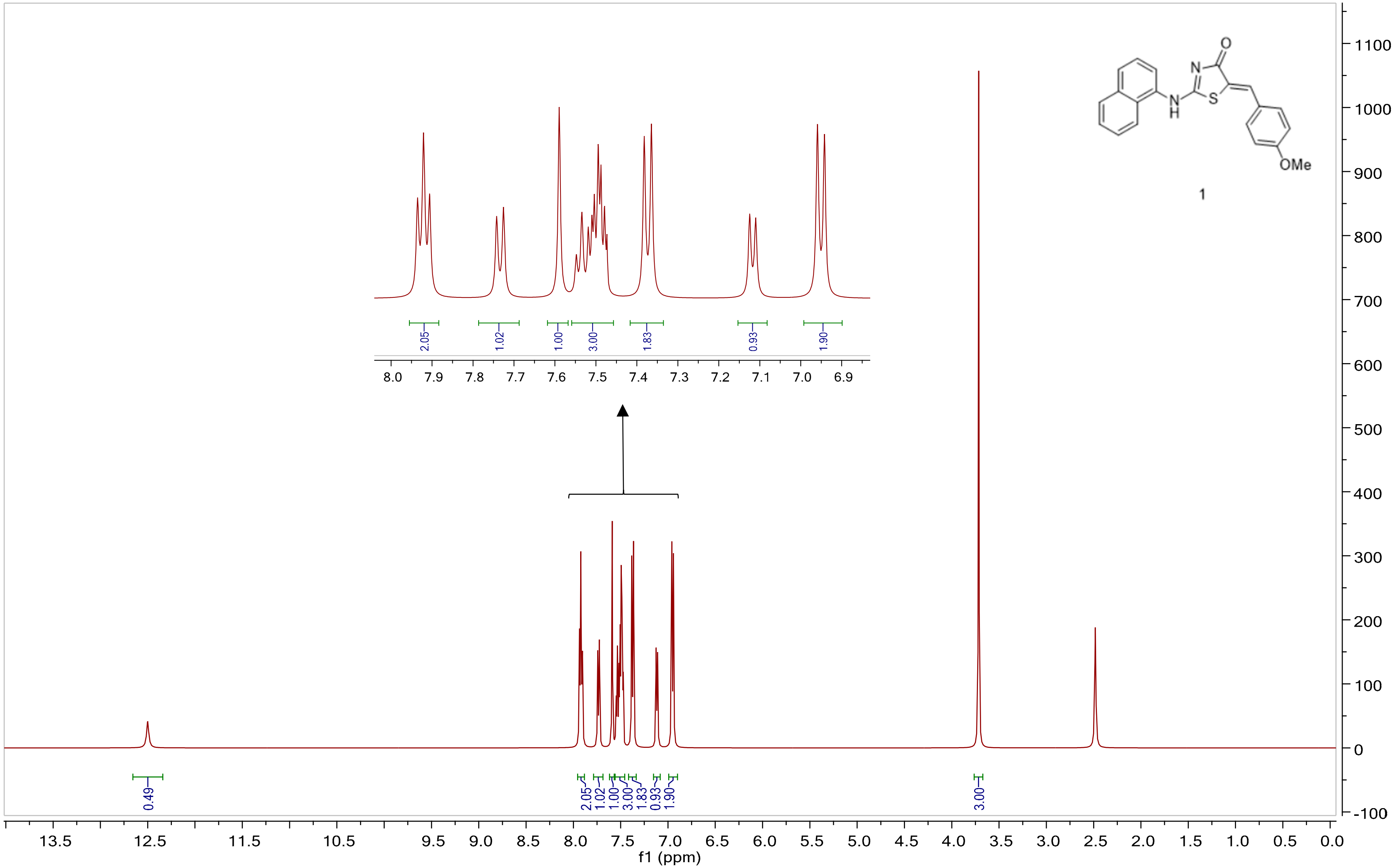

Figure S1. ^{1}H NMR spectrum of analog **1**

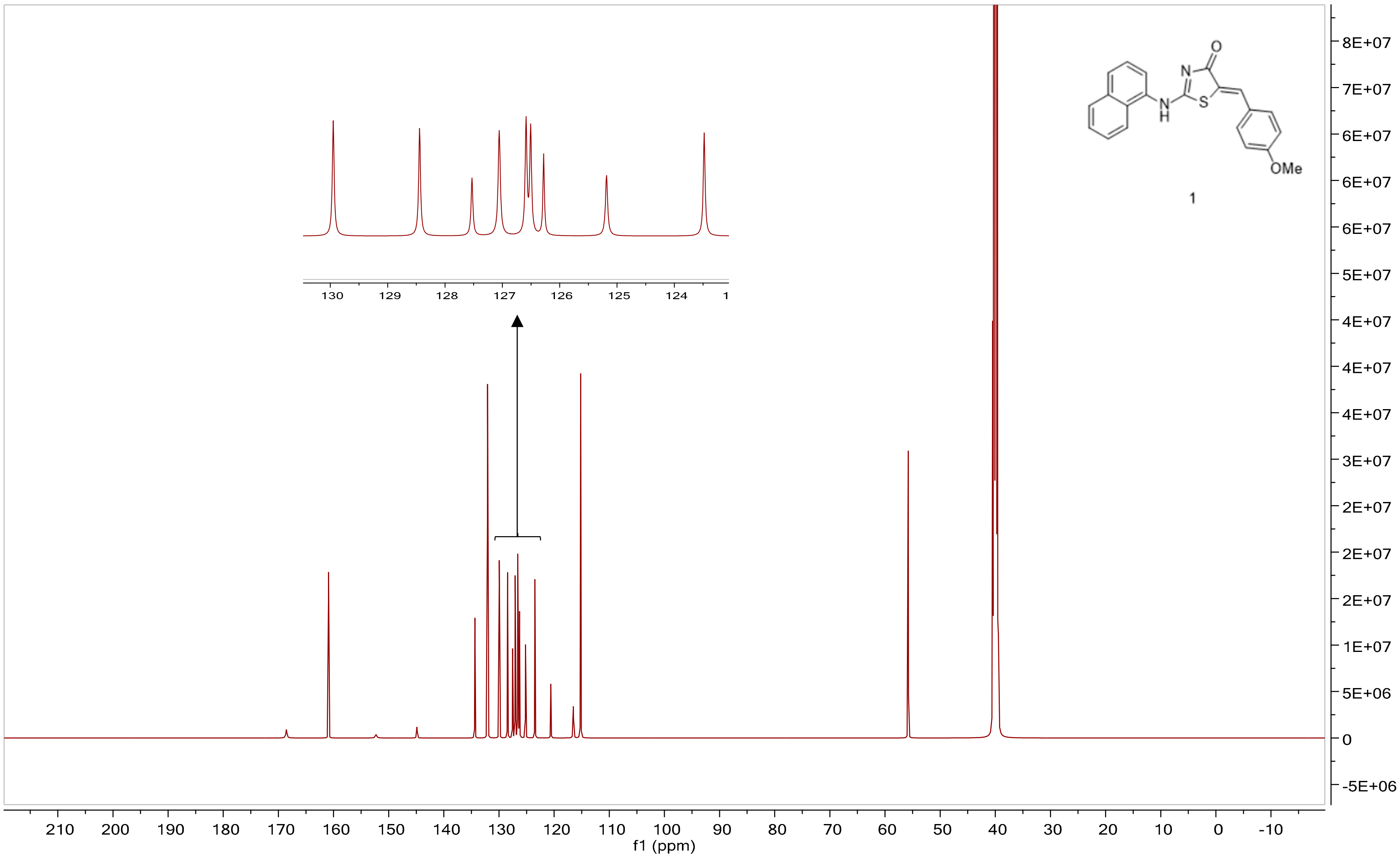

Figure S2. ^{13}C NMR spectrum of analog **1**

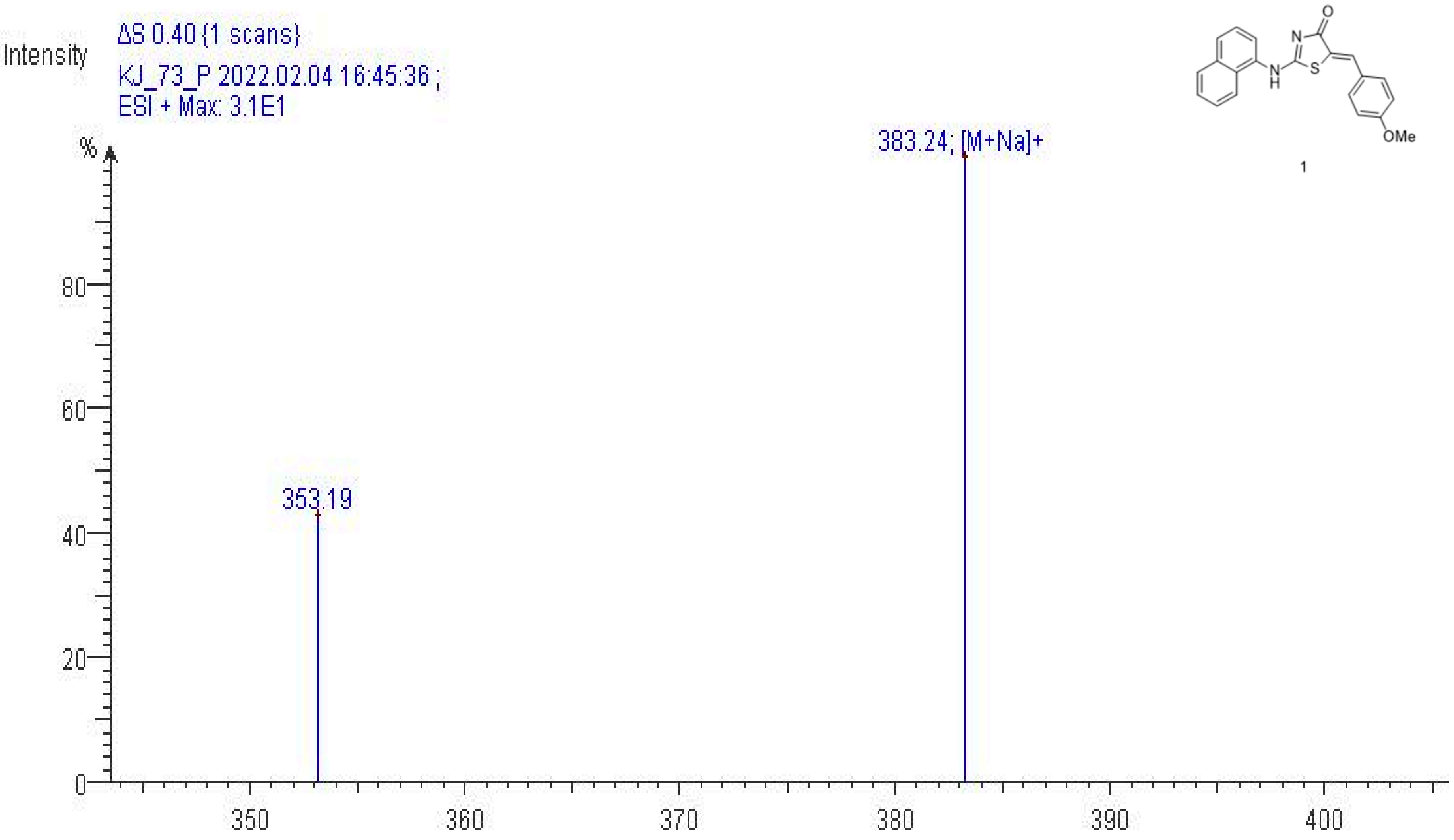

Figure S3. LRMS (ESI+) spectrum of analog **1**

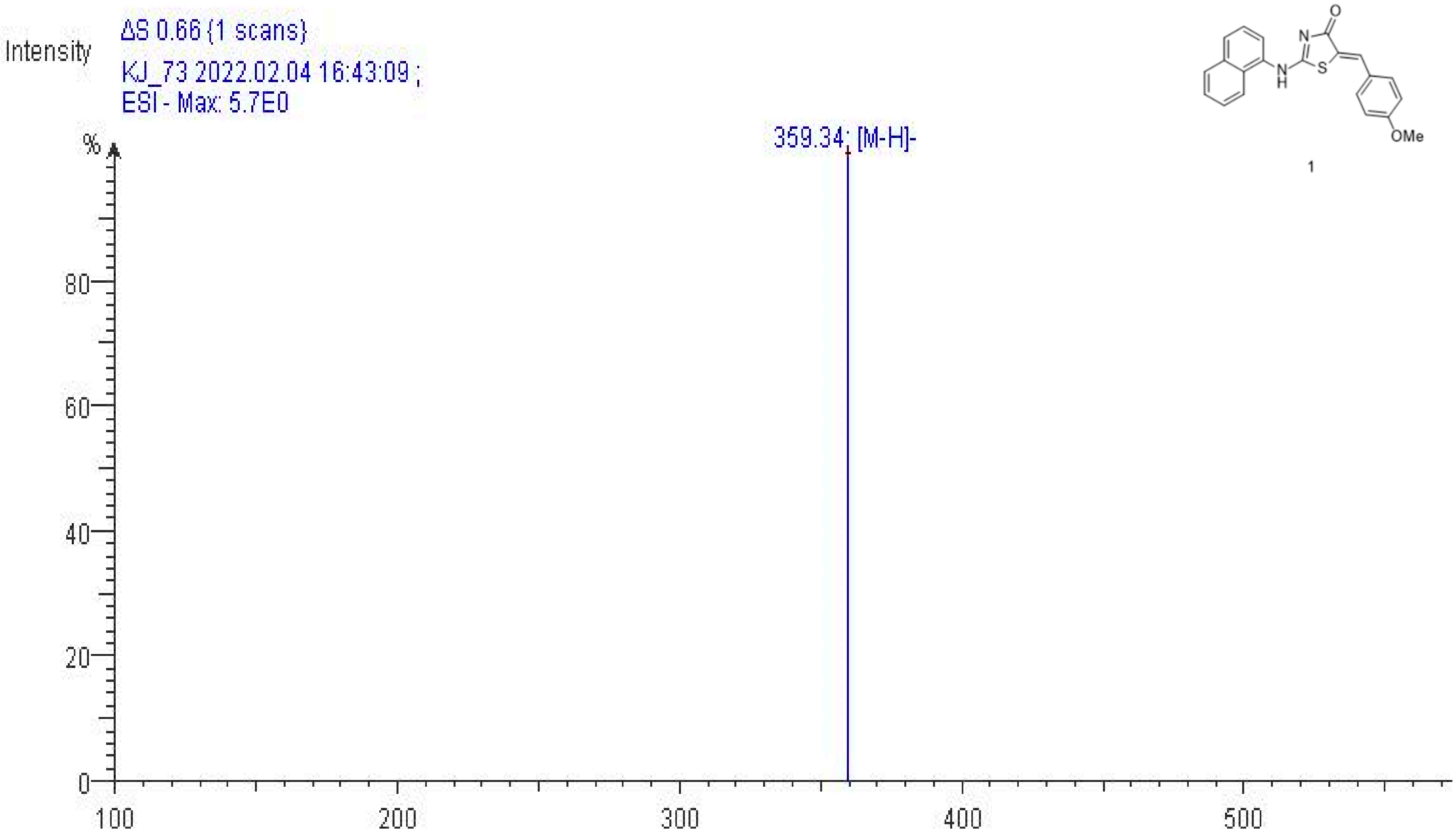

Figure S4. LRMS (ESI–) spectrum of analog **1**

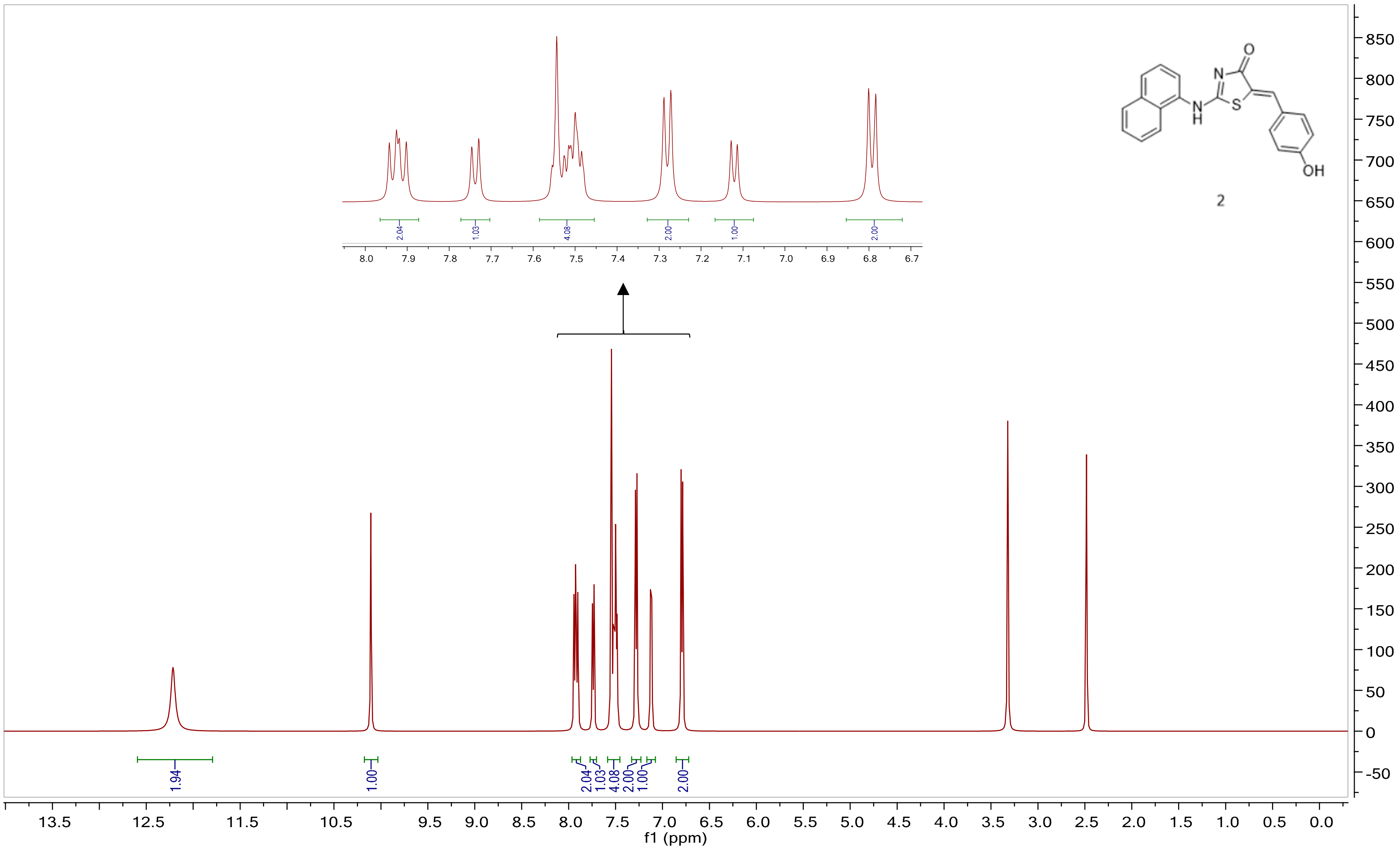

Figure S5. ^{1}H NMR spectrum of analog **2**

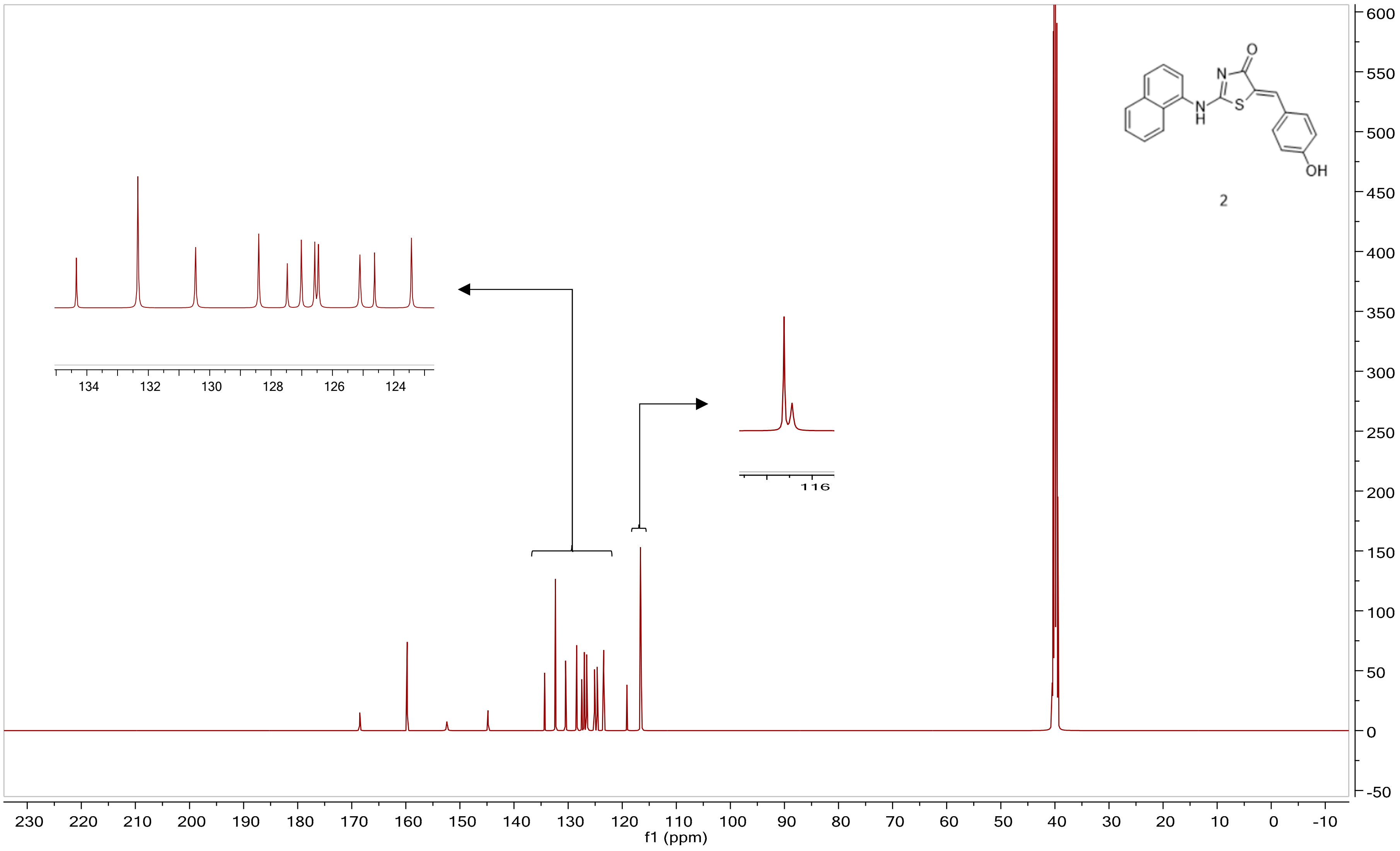

Figure S6. ^{13}C NMR spectrum of analog **2**

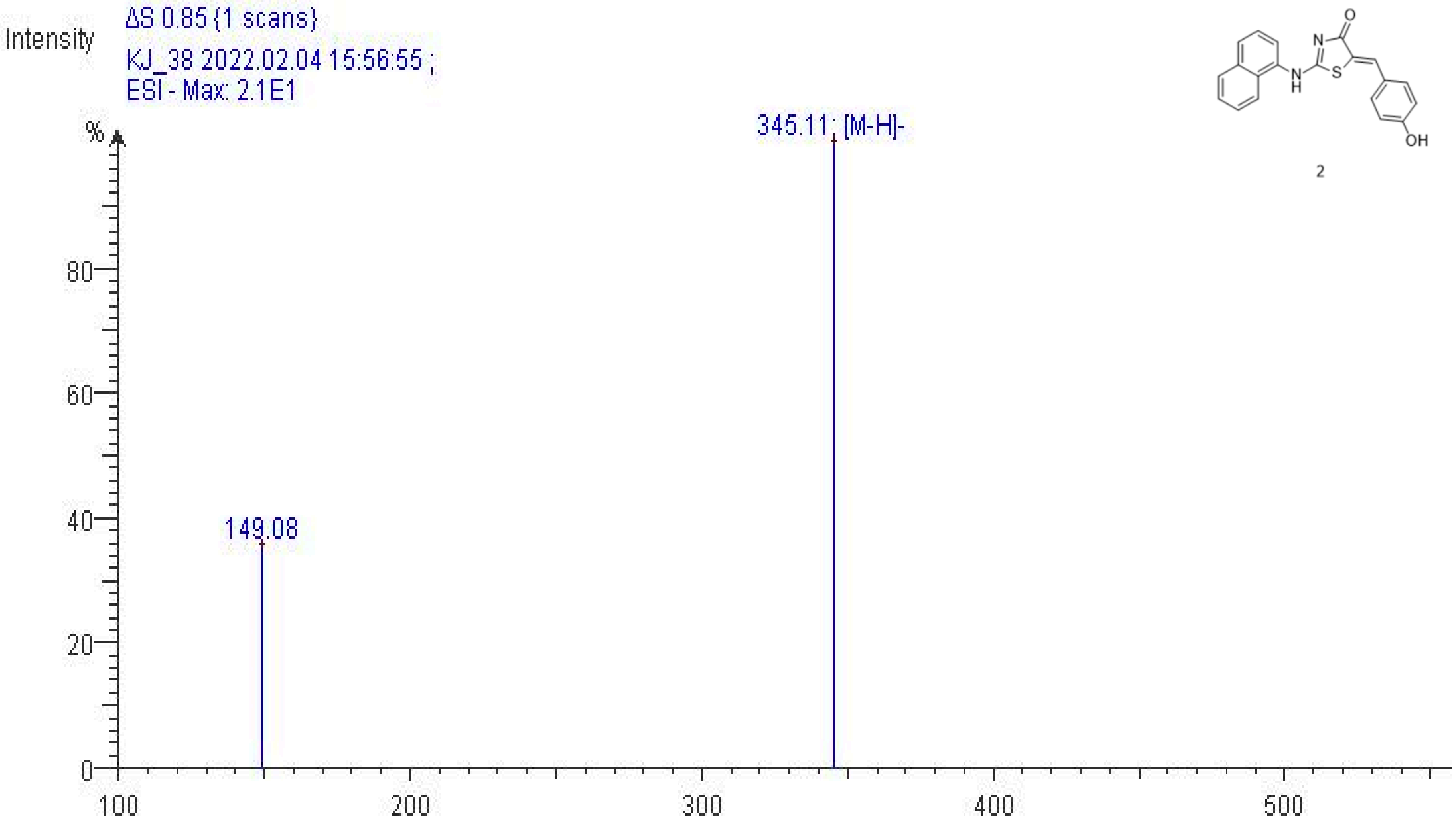

Figure S7. LRMS (ESI–) spectrum of analog **2**

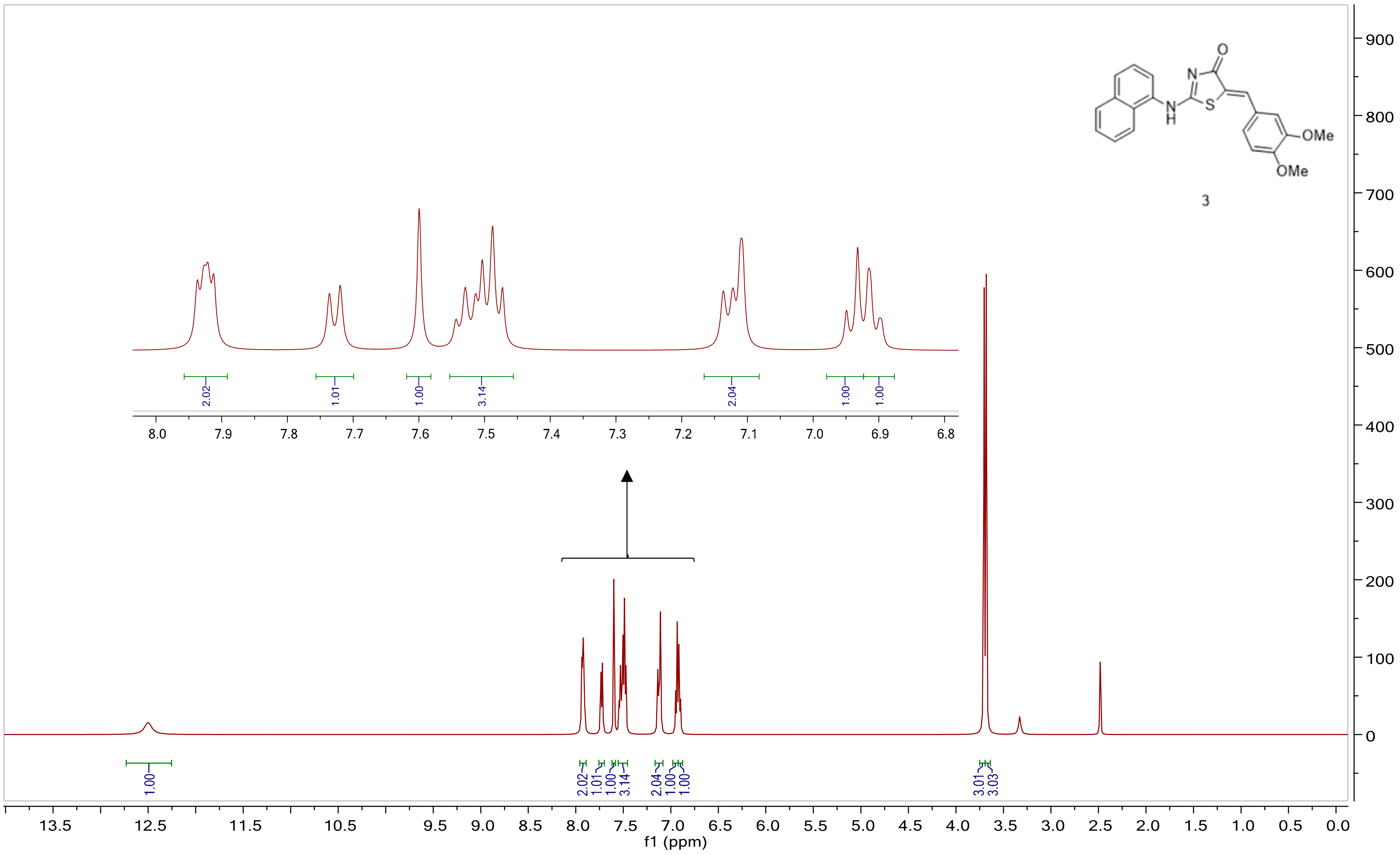

Figure S8. ^{1}H NMR spectrum of analog **3**

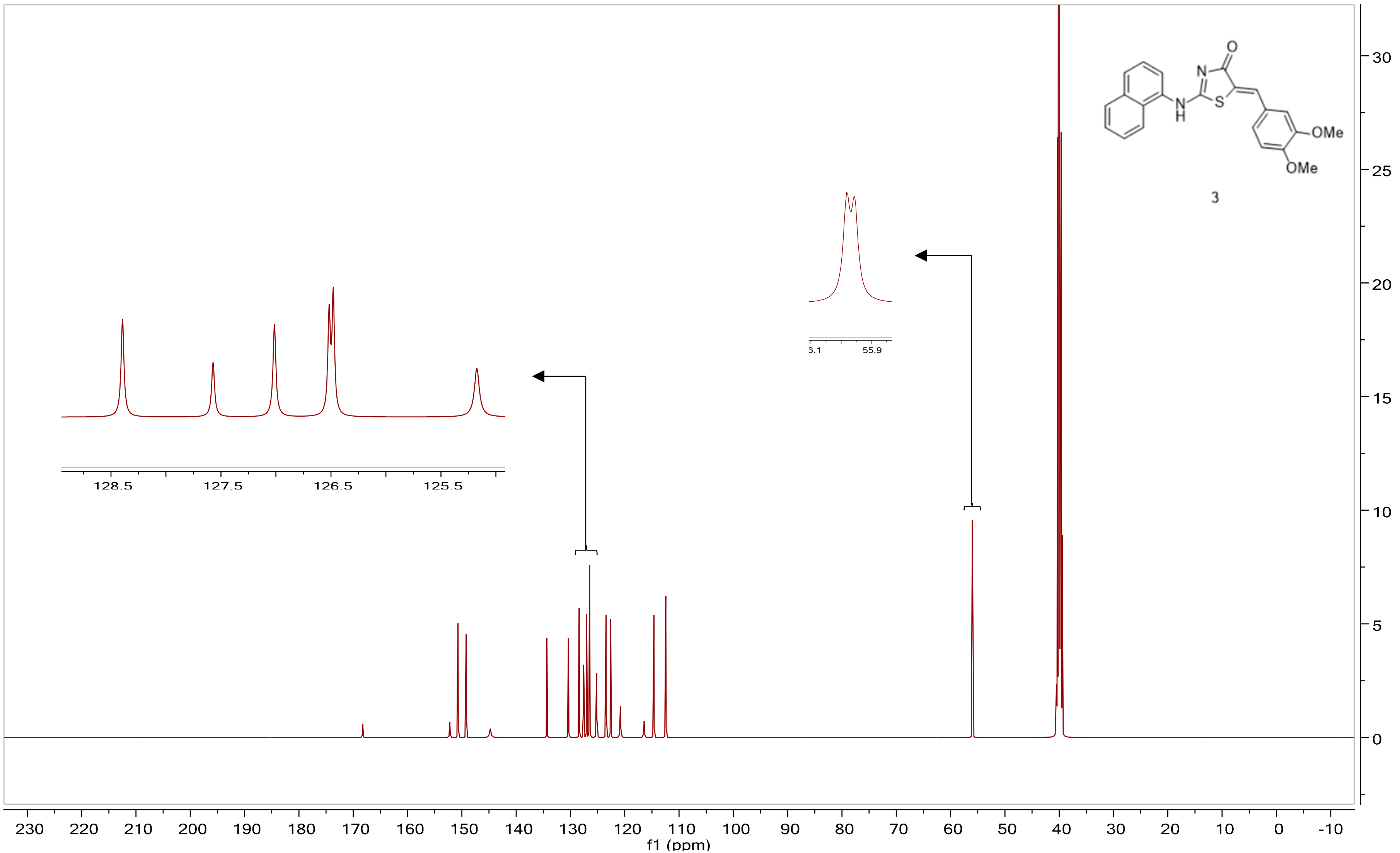

Figure S9. ^{13}C NMR spectrum of analog **3**

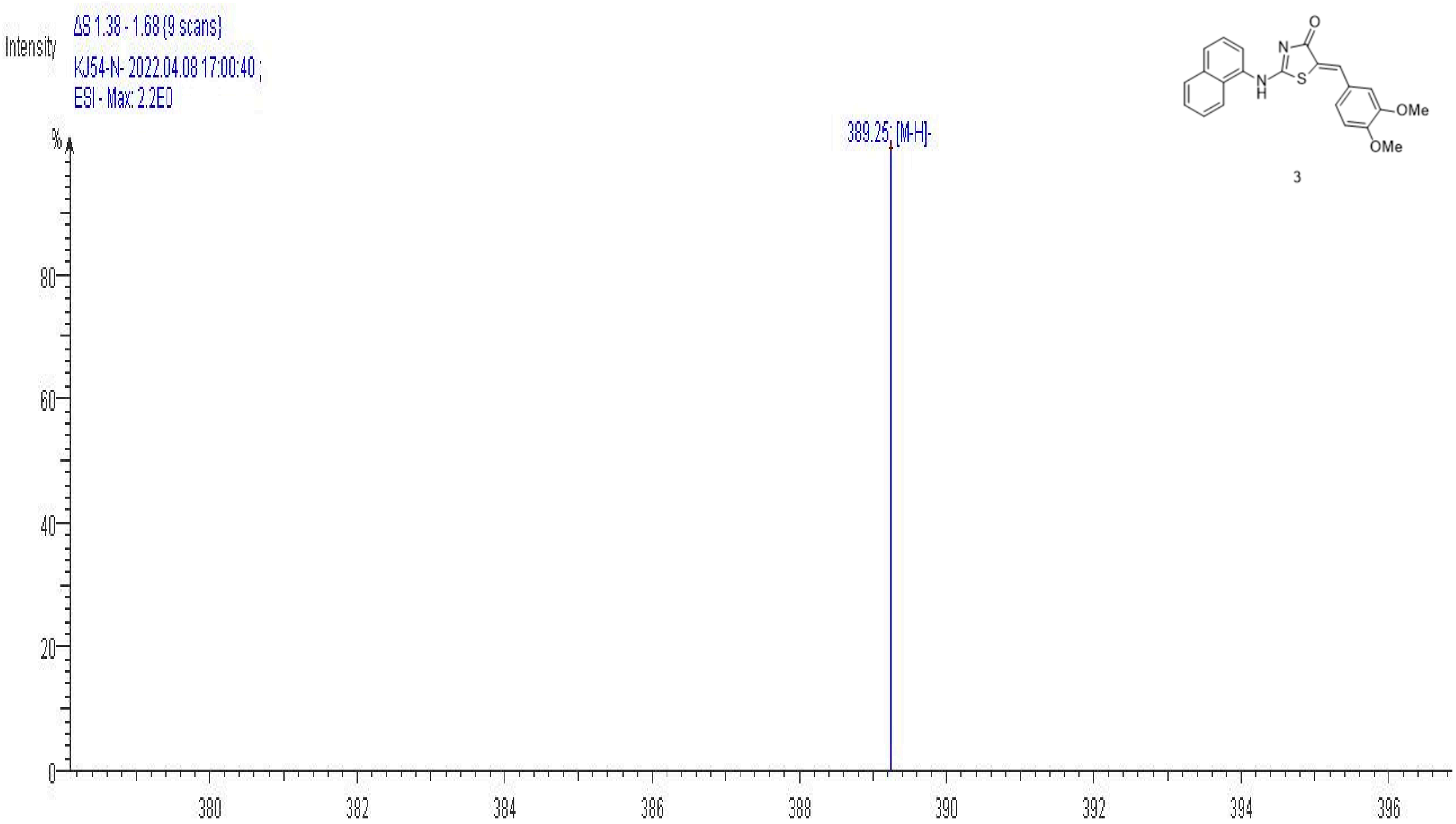

3

Figure S10. LRMS (ESI–) spectrum of analog **3**

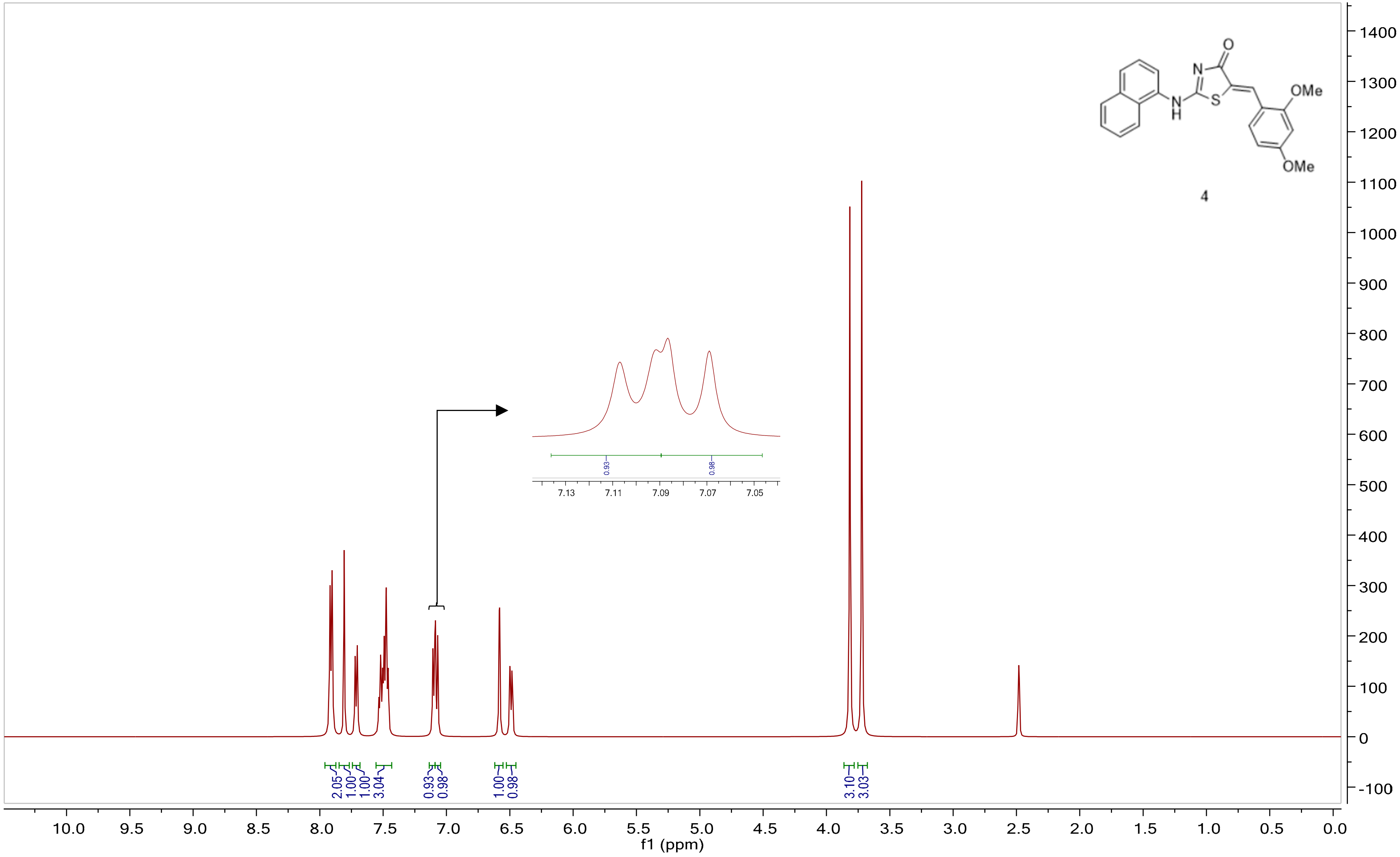

Figure S11. ^{1}H NMR spectrum of analog **4**

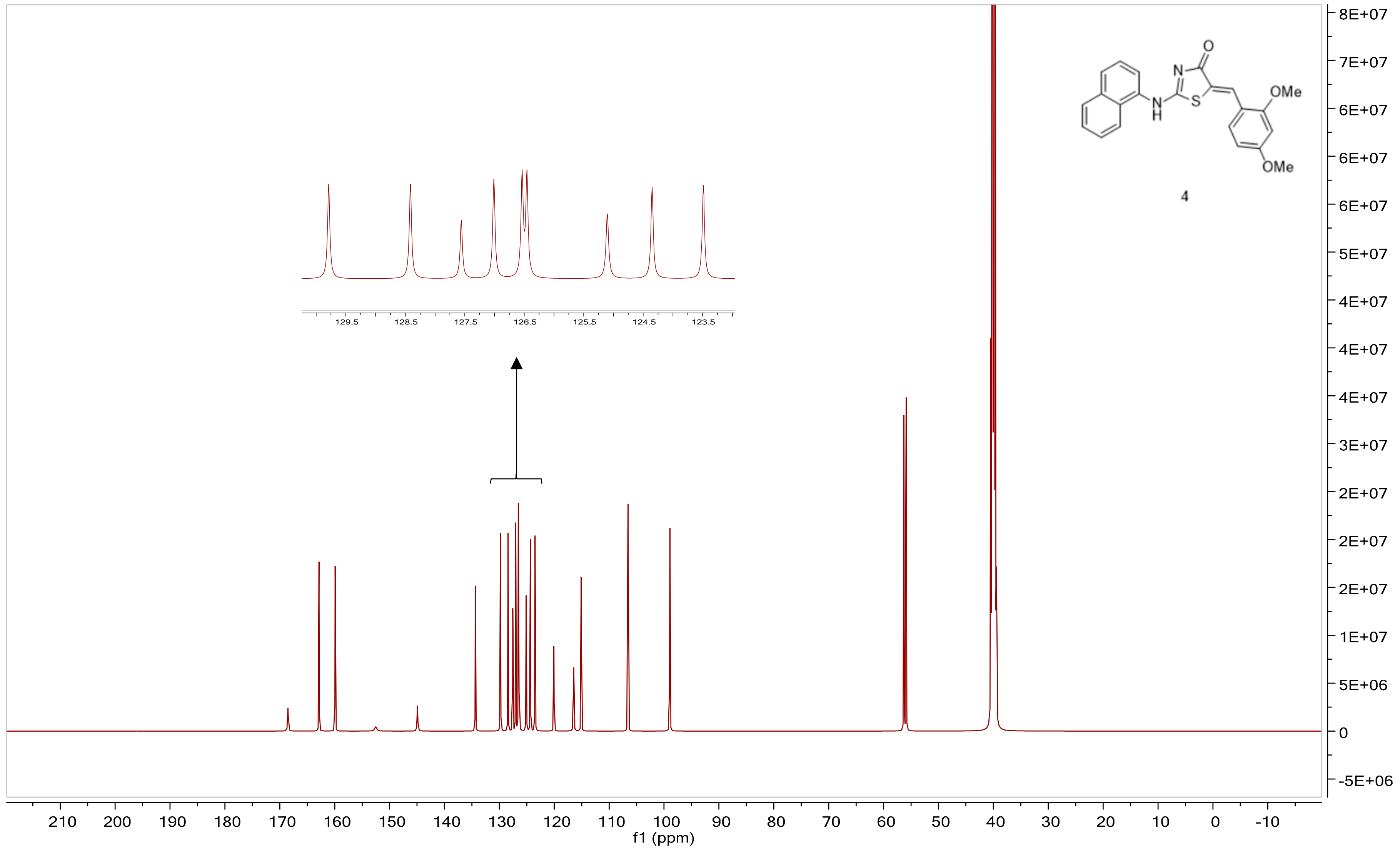

Figure S12. ^{13}C NMR spectrum of analog **4**

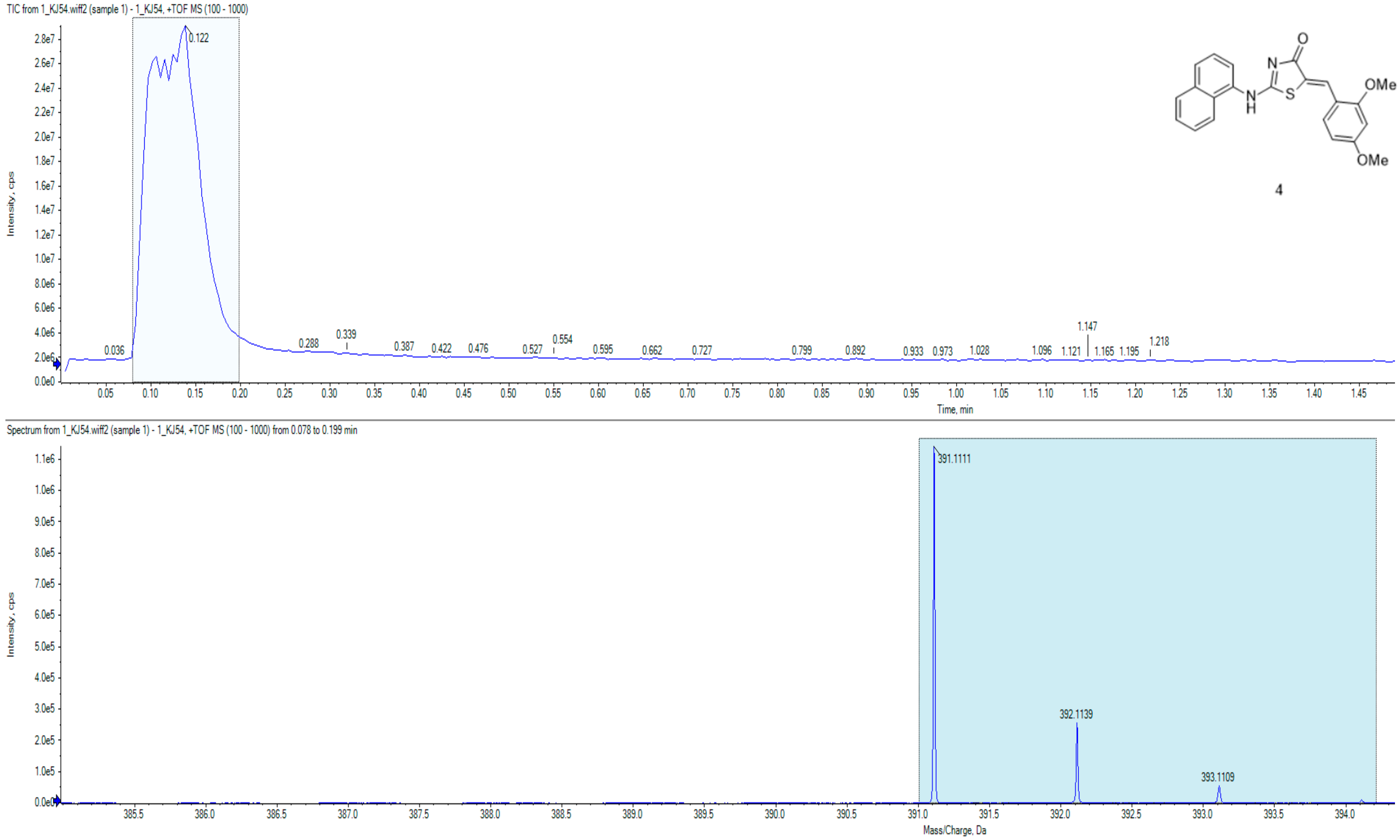

Figure S13-1. HRMS (ESI+) spectrum of analog **4**

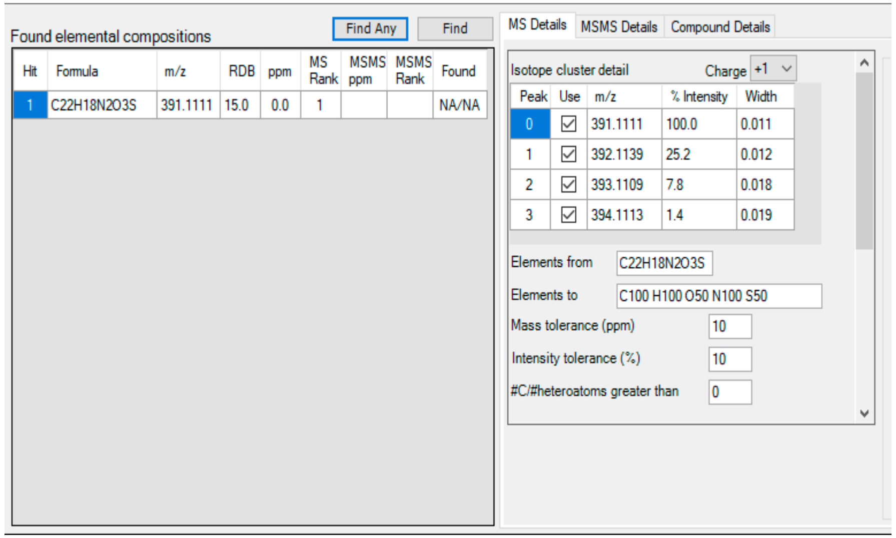

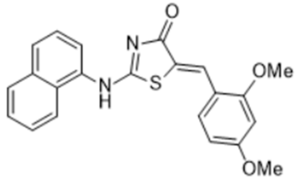

4

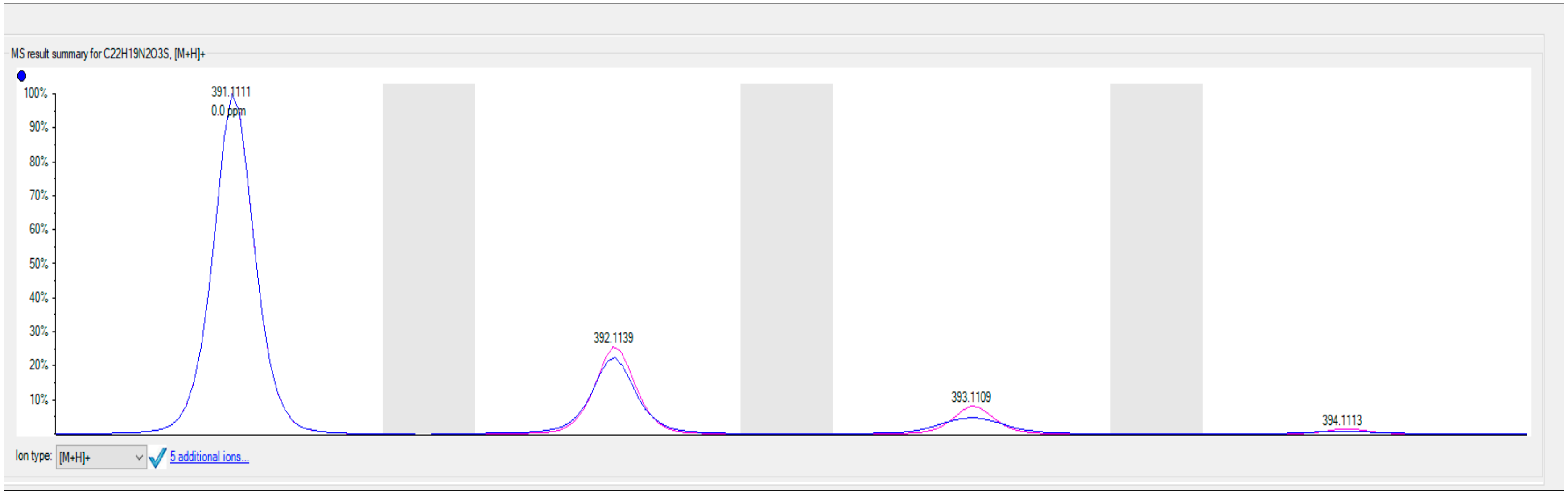

Figure S13-2. HRMS (ESI+) spectrum of analog **4**

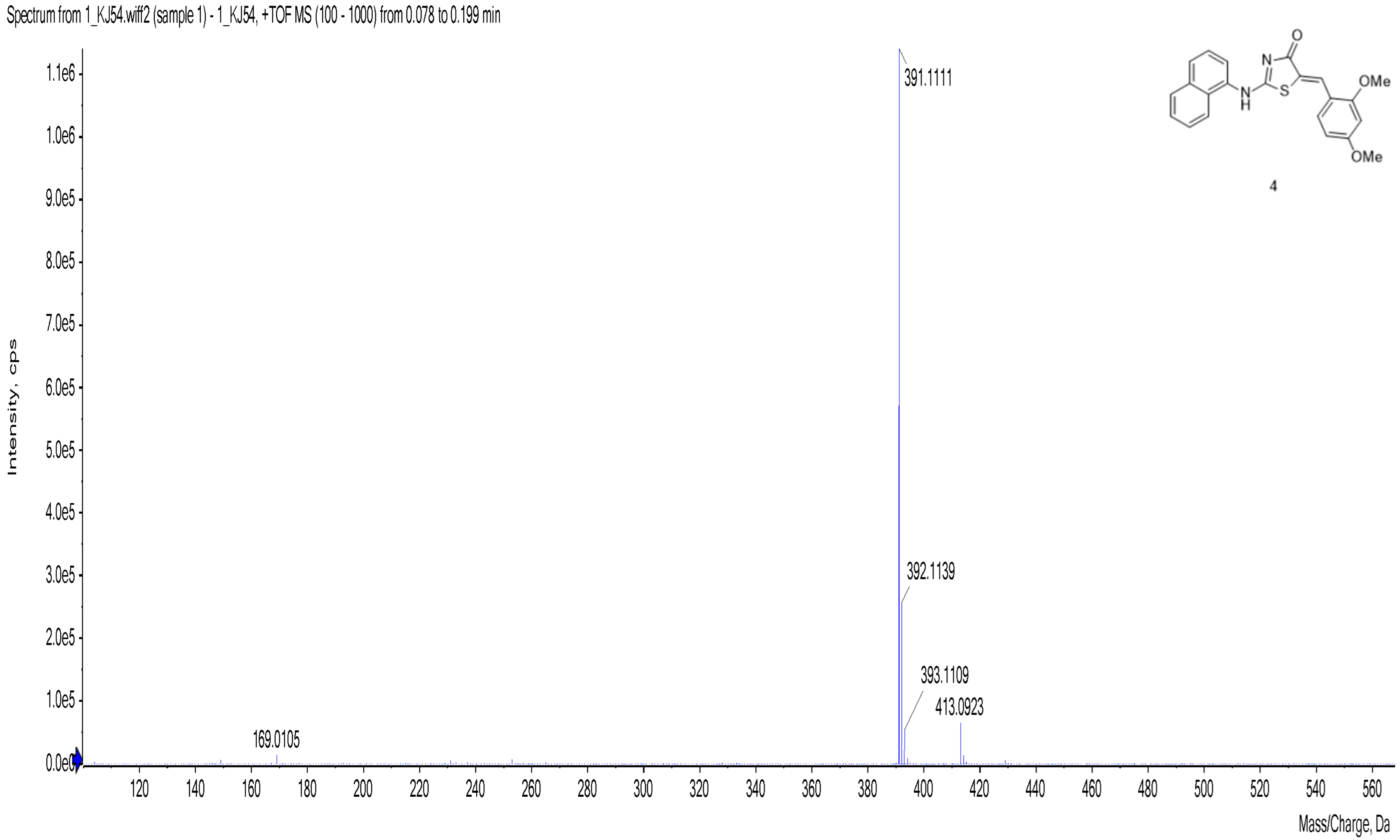

Figure S13-3. HRMS (ESI+) spectrum of analog **4**

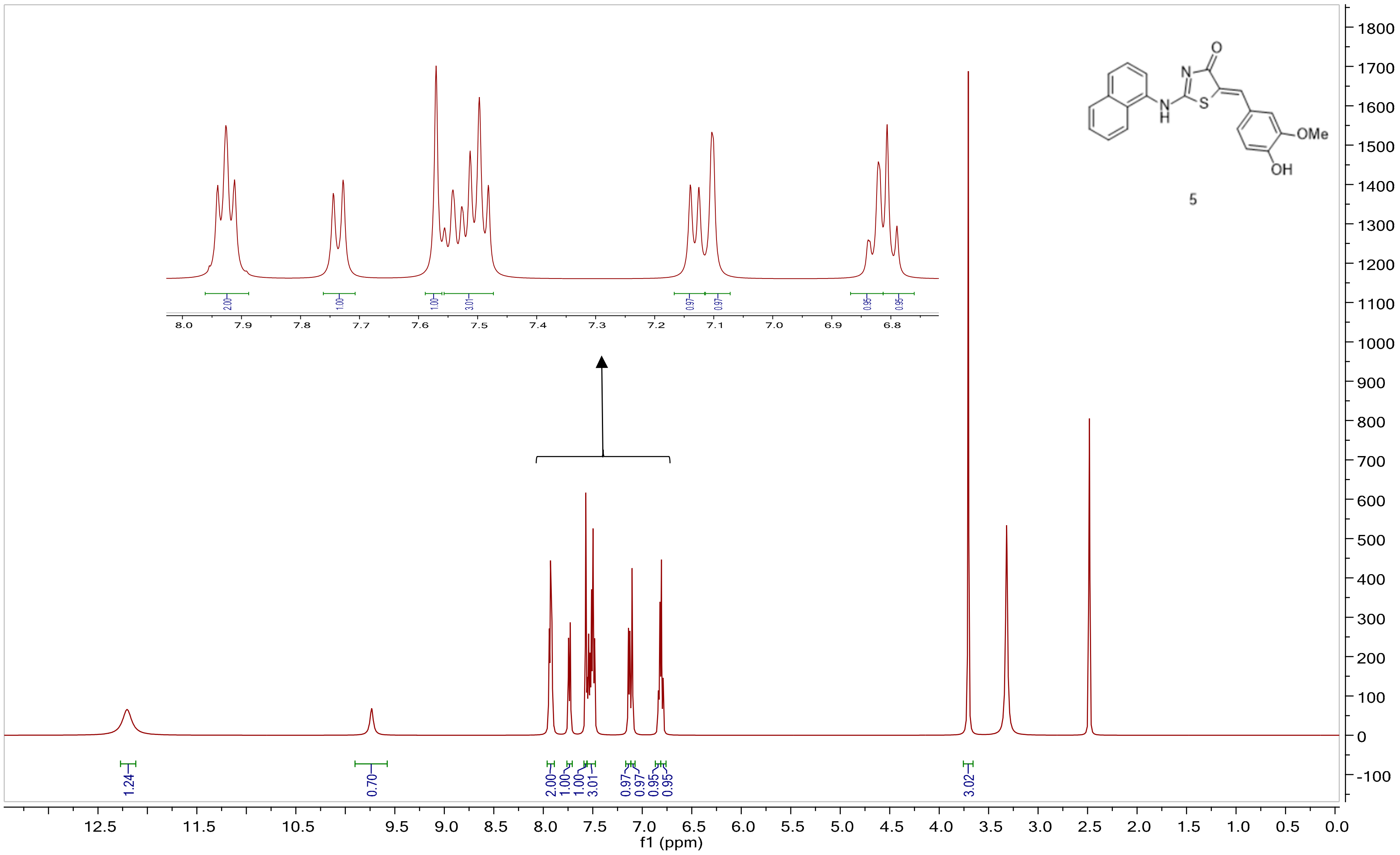

Figure S14. ¹H NMR spectrum of analog **5**

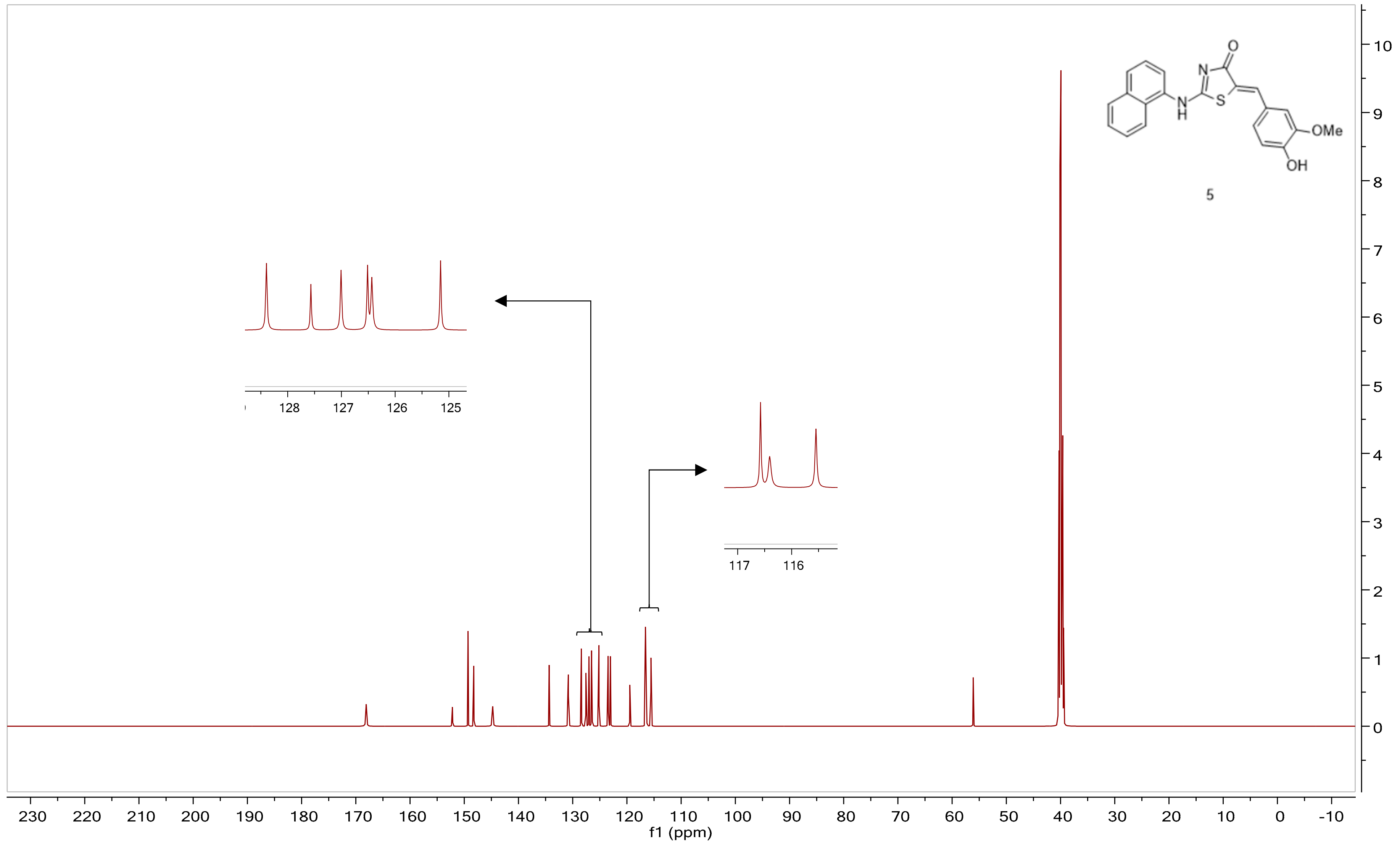

Figure S15. ^{13}C NMR spectrum of analog **5**

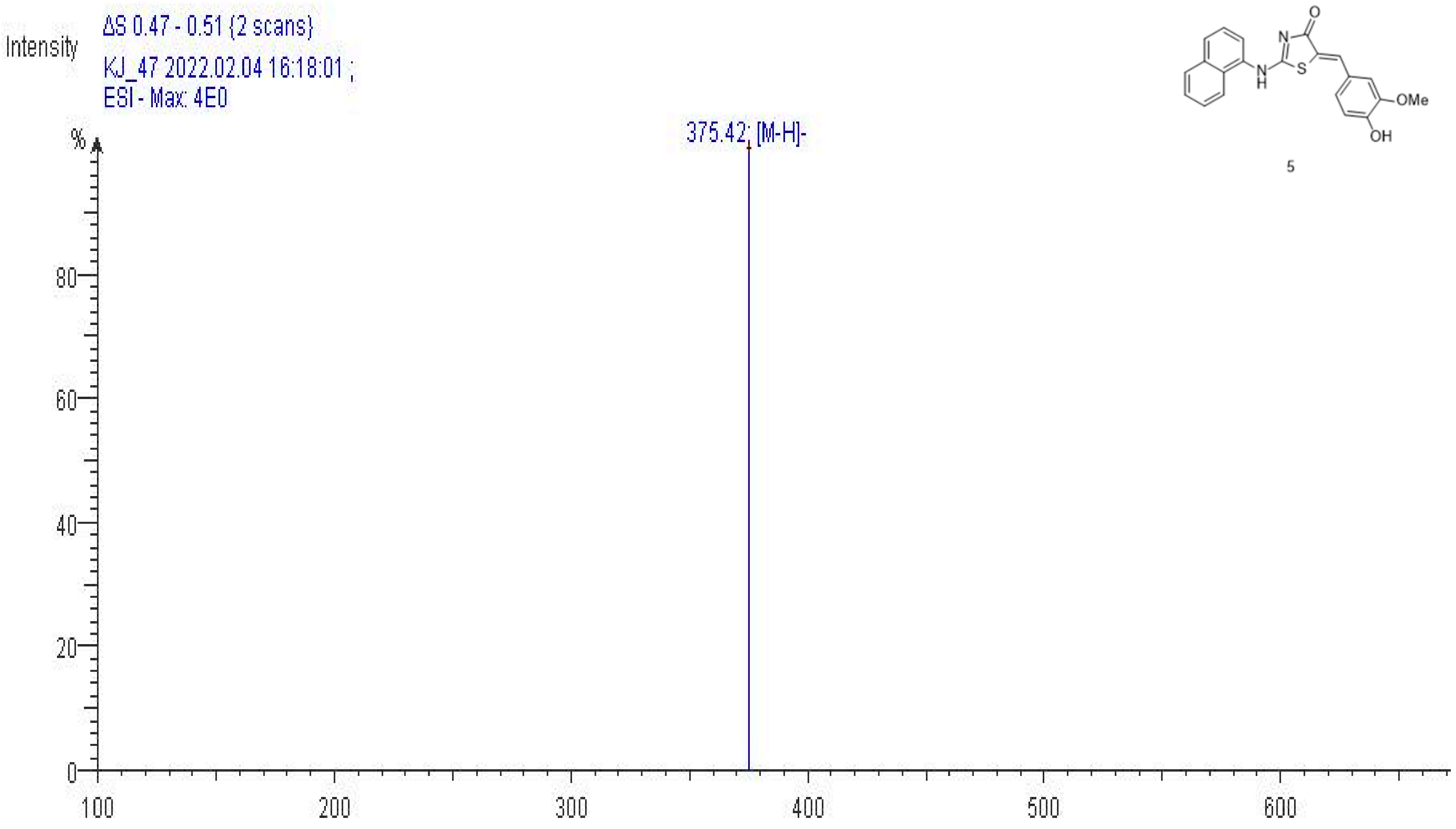

5

Figure S16. LRMS (ESI–) spectrum of analog **5**

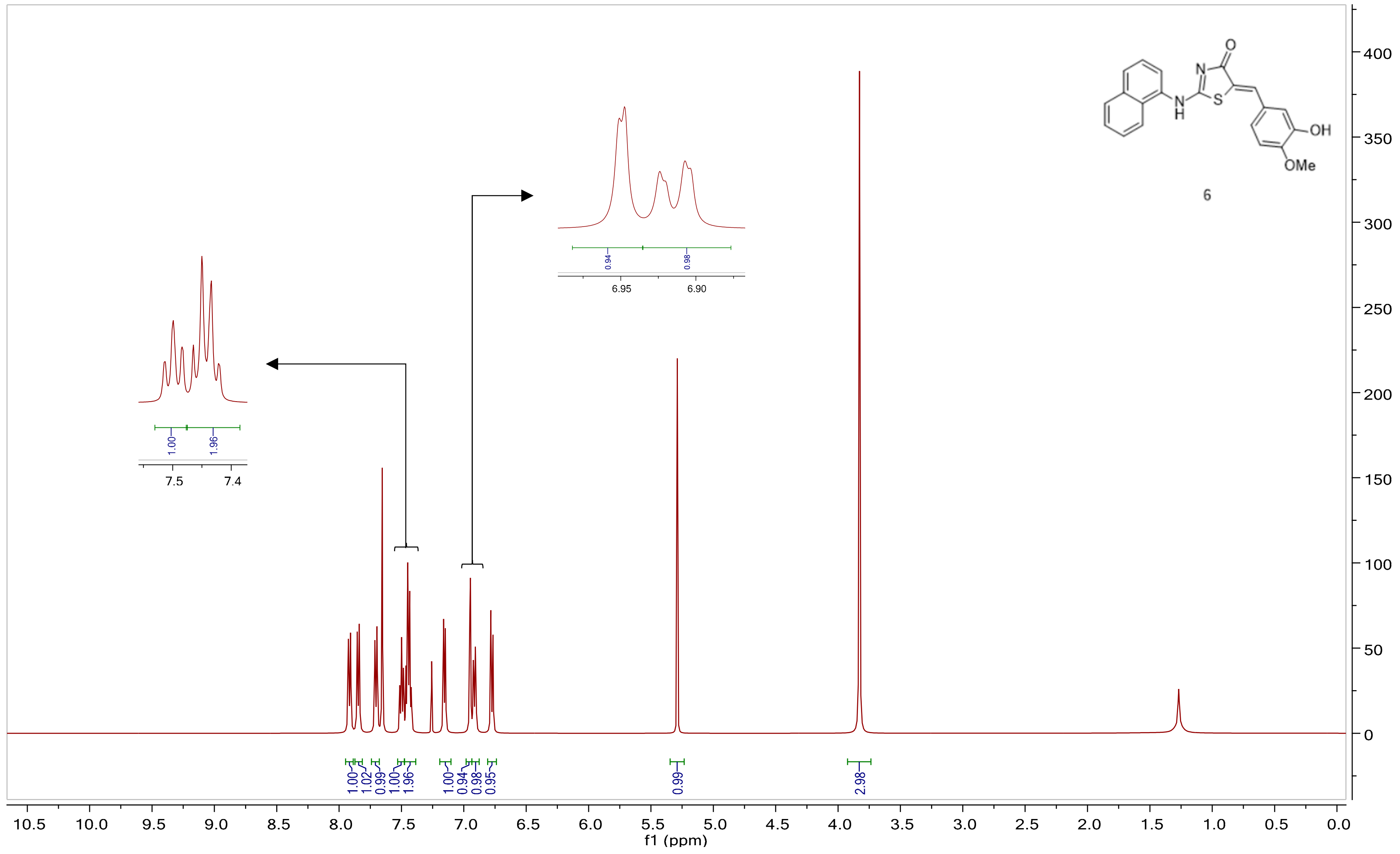

Figure S17. ^{1}H NMR spectrum of analog **6**

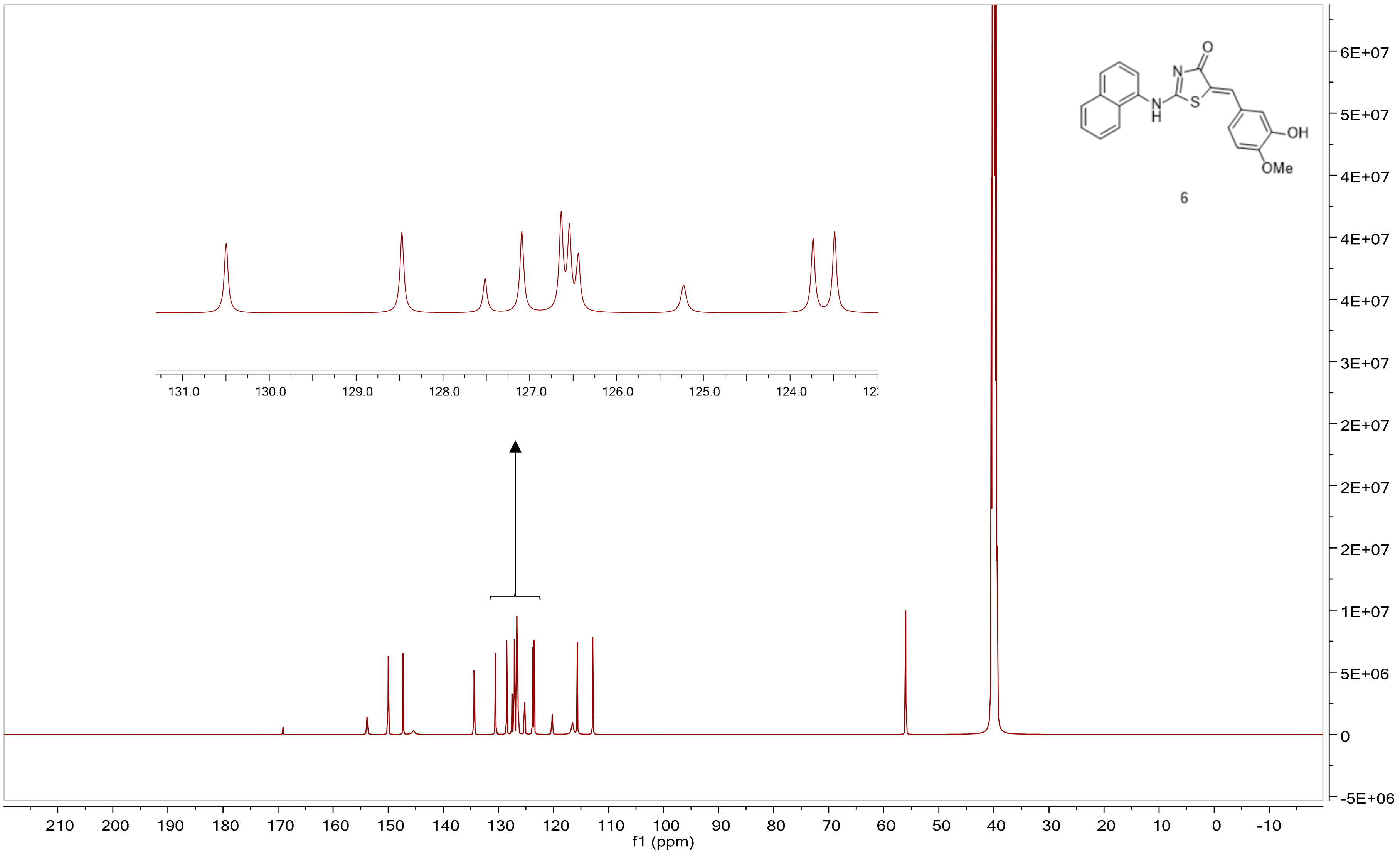

Figure S18. ^{13}C NMR spectrum of analog **6**

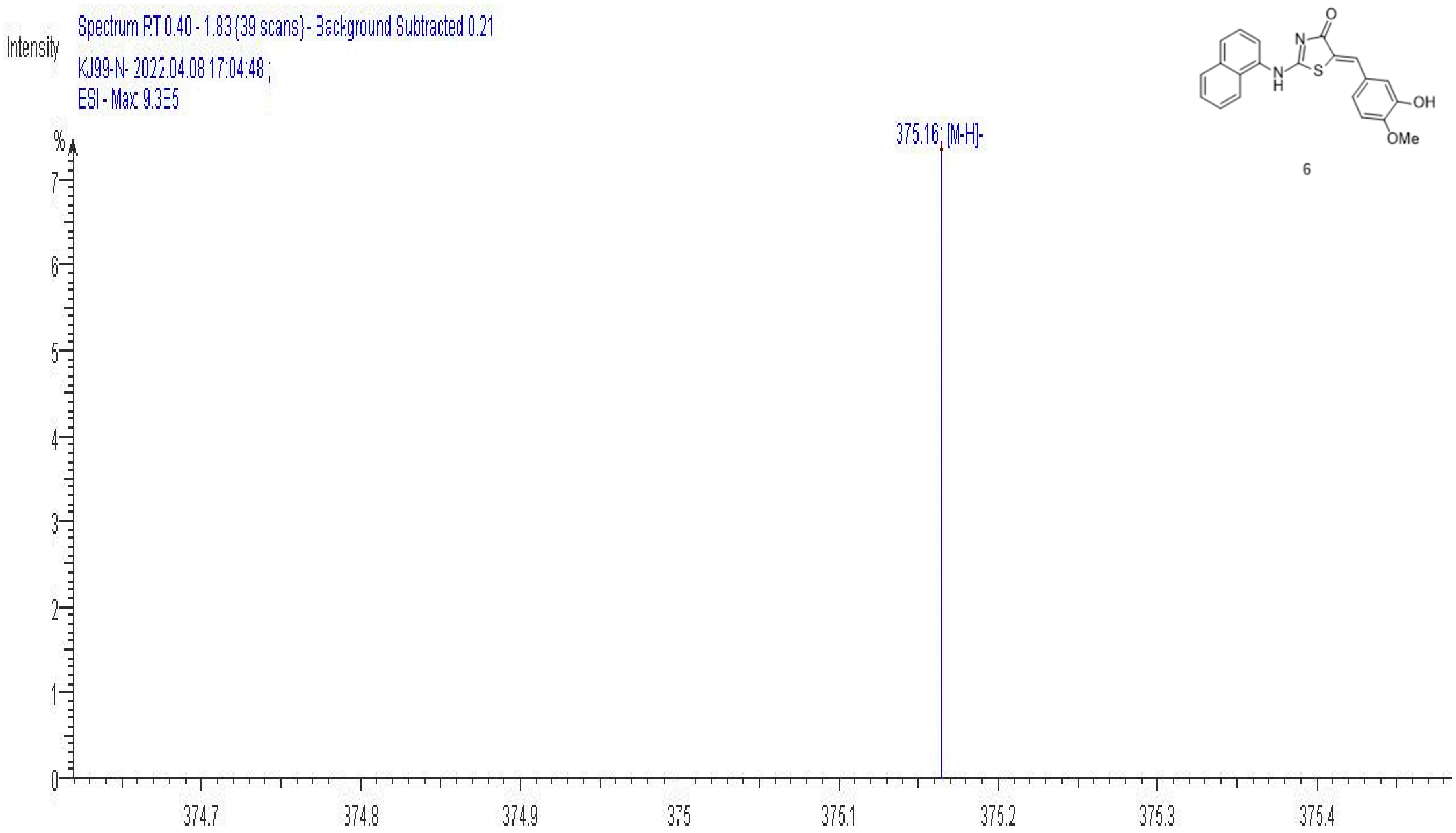

6

Figure S19. LRMS (ESI–) spectrum of analog **6**

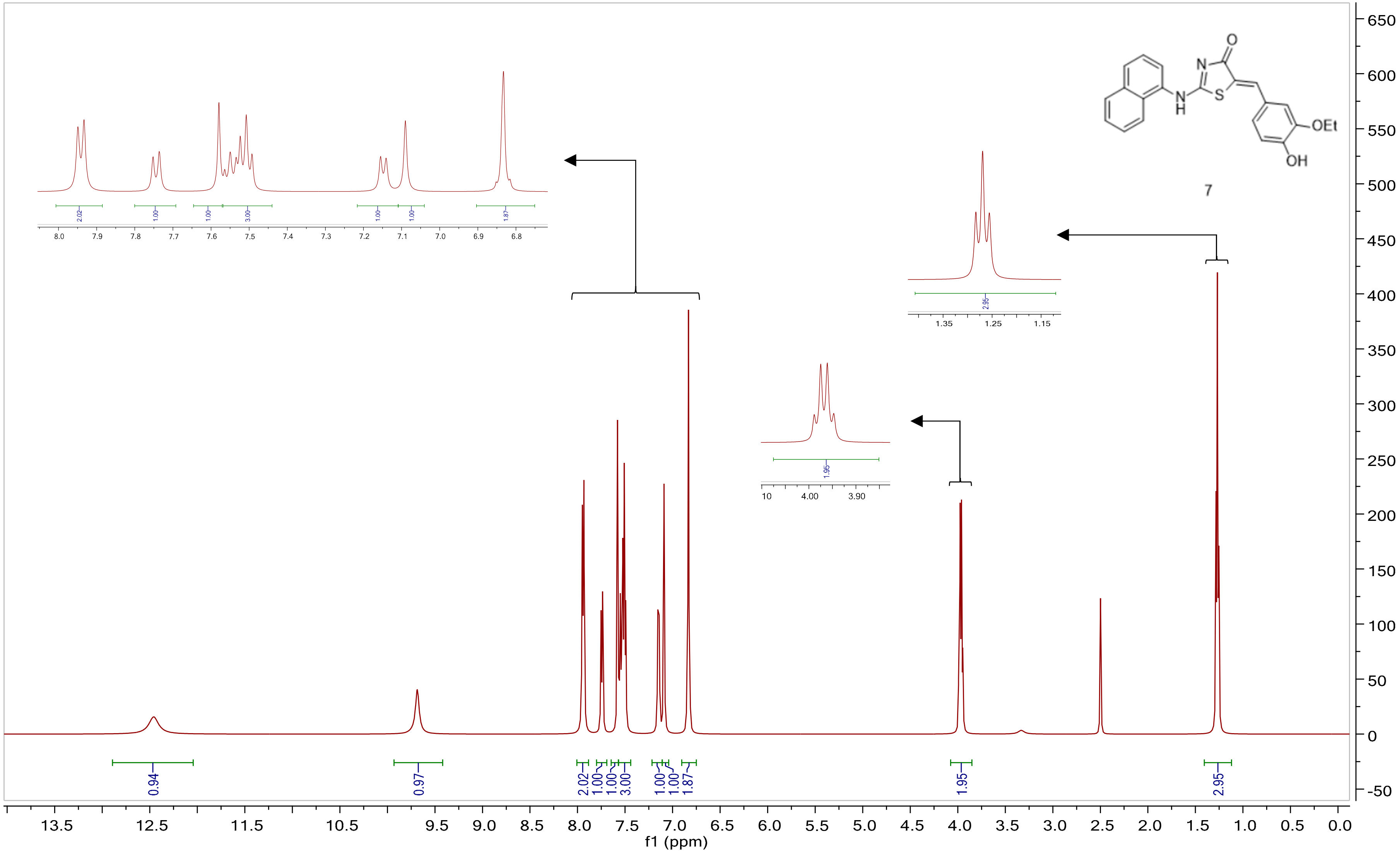

Figure S20. ^{1}H NMR spectrum of analog **7**

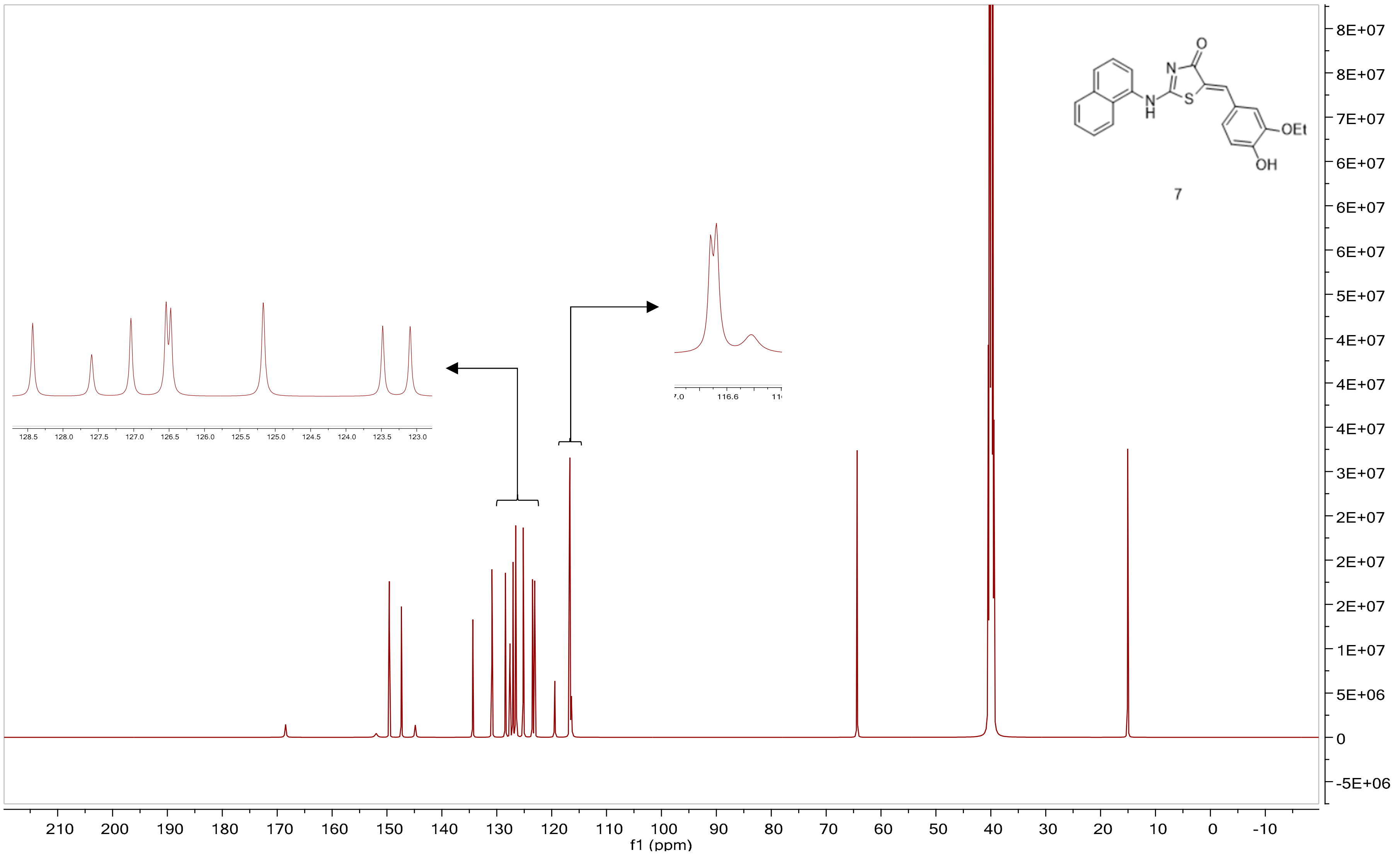

Figure S21. ^{13}C NMR spectrum of analog **7**

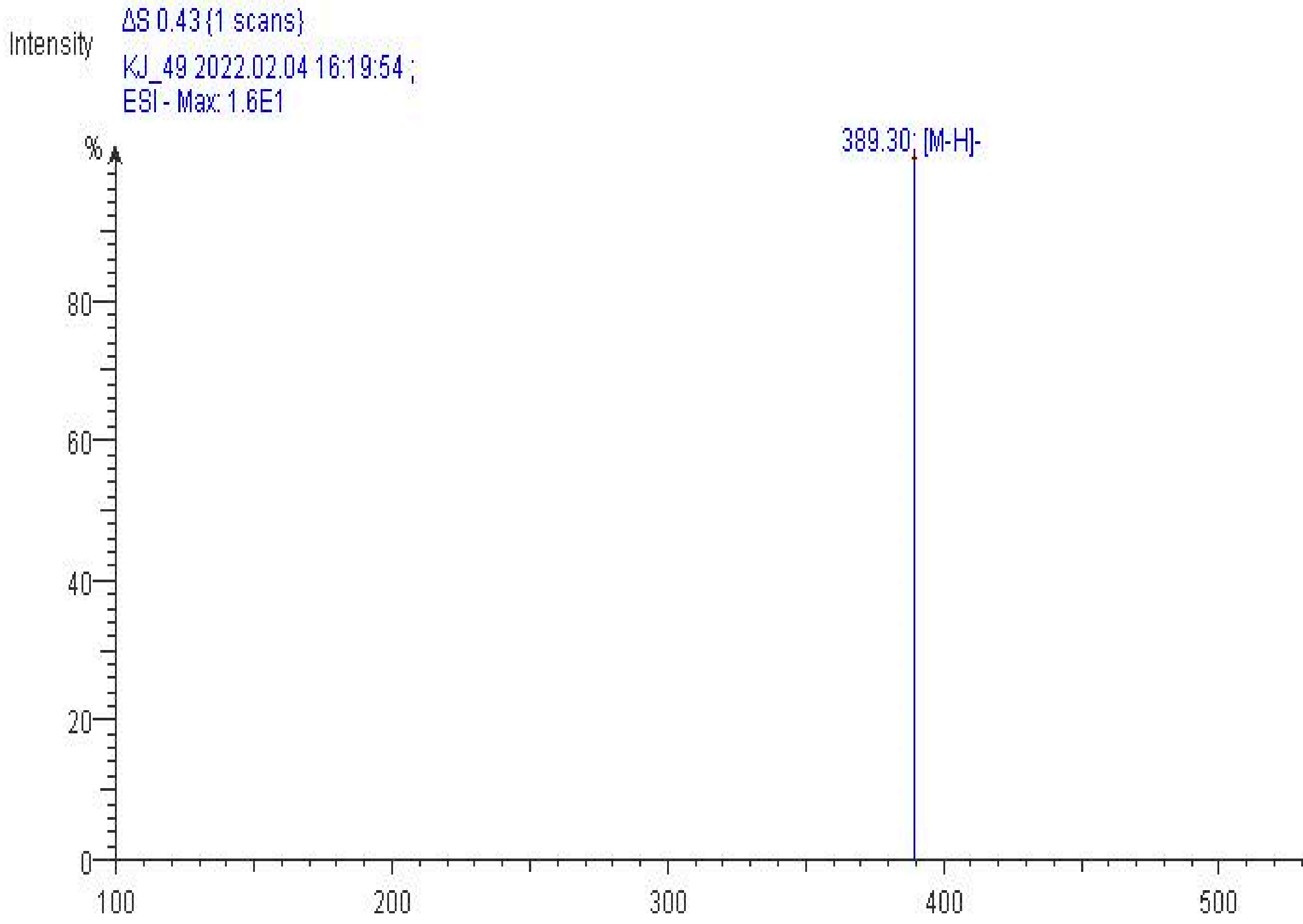

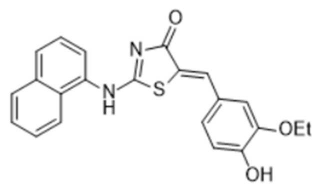

7

Figure S22. LRMS (ESI–) spectrum of analog **7**

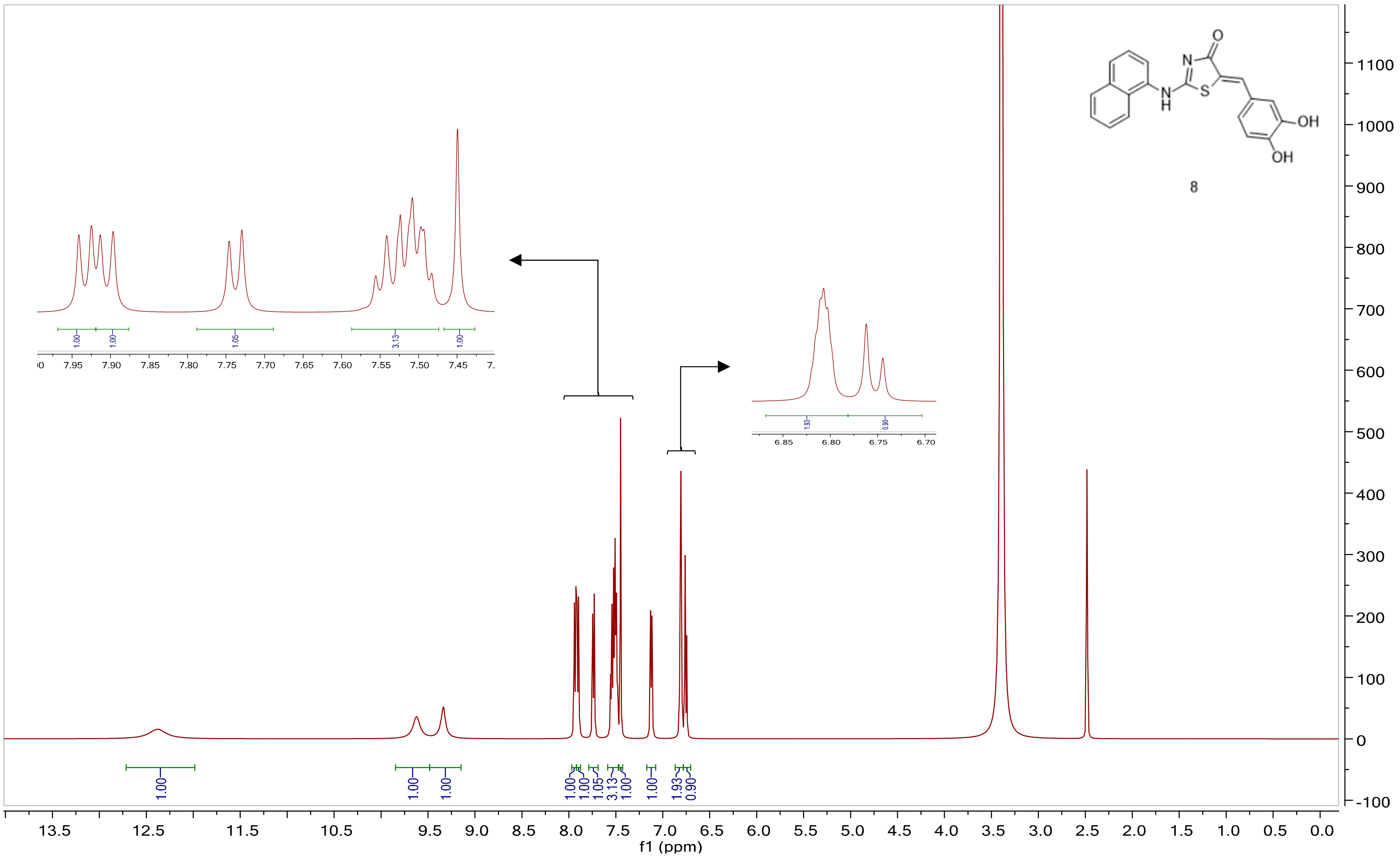

Figure S23. ^{1}H NMR spectrum of analog **8**

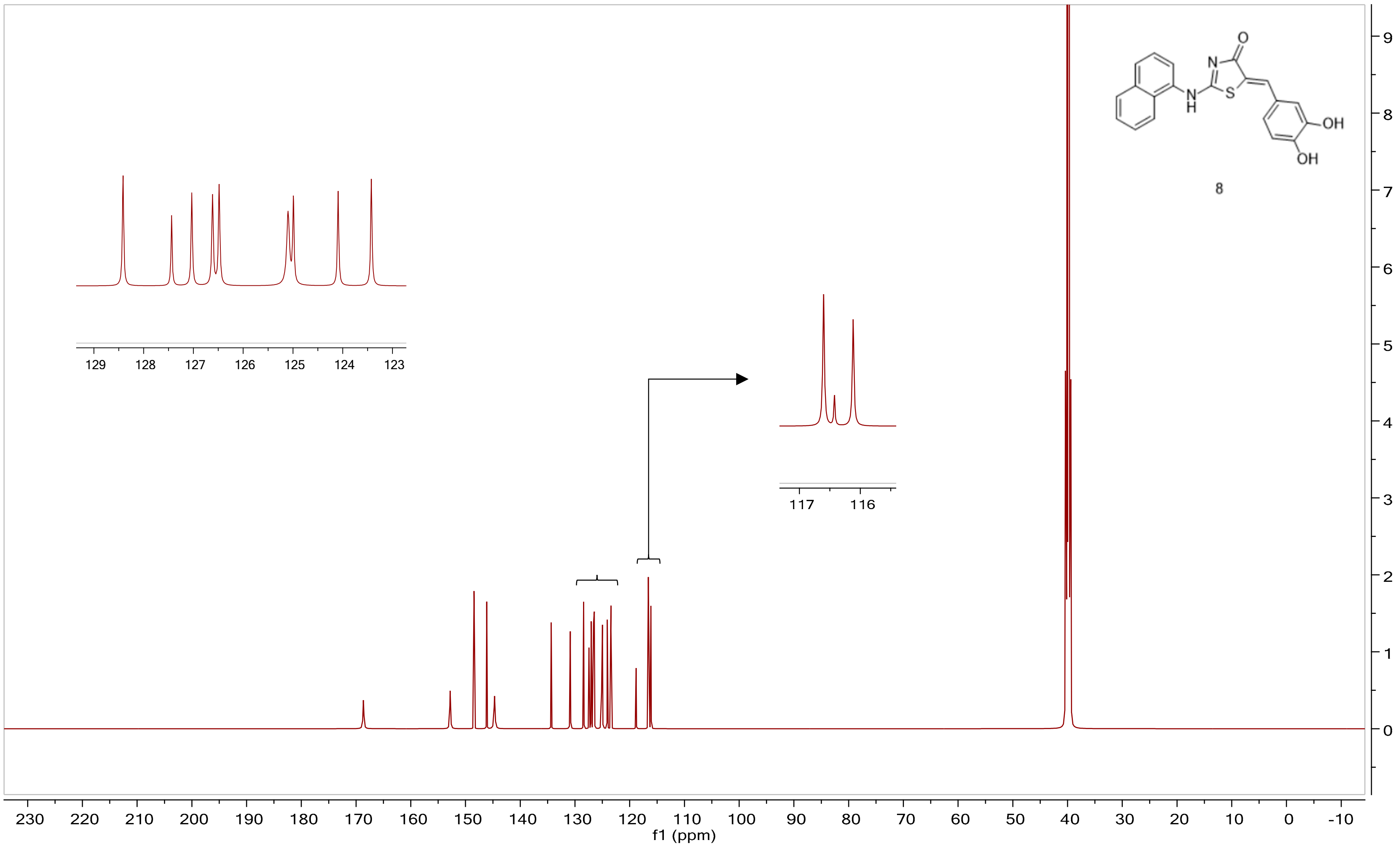

Figure S24. ^{13}C NMR spectrum of analog **8**

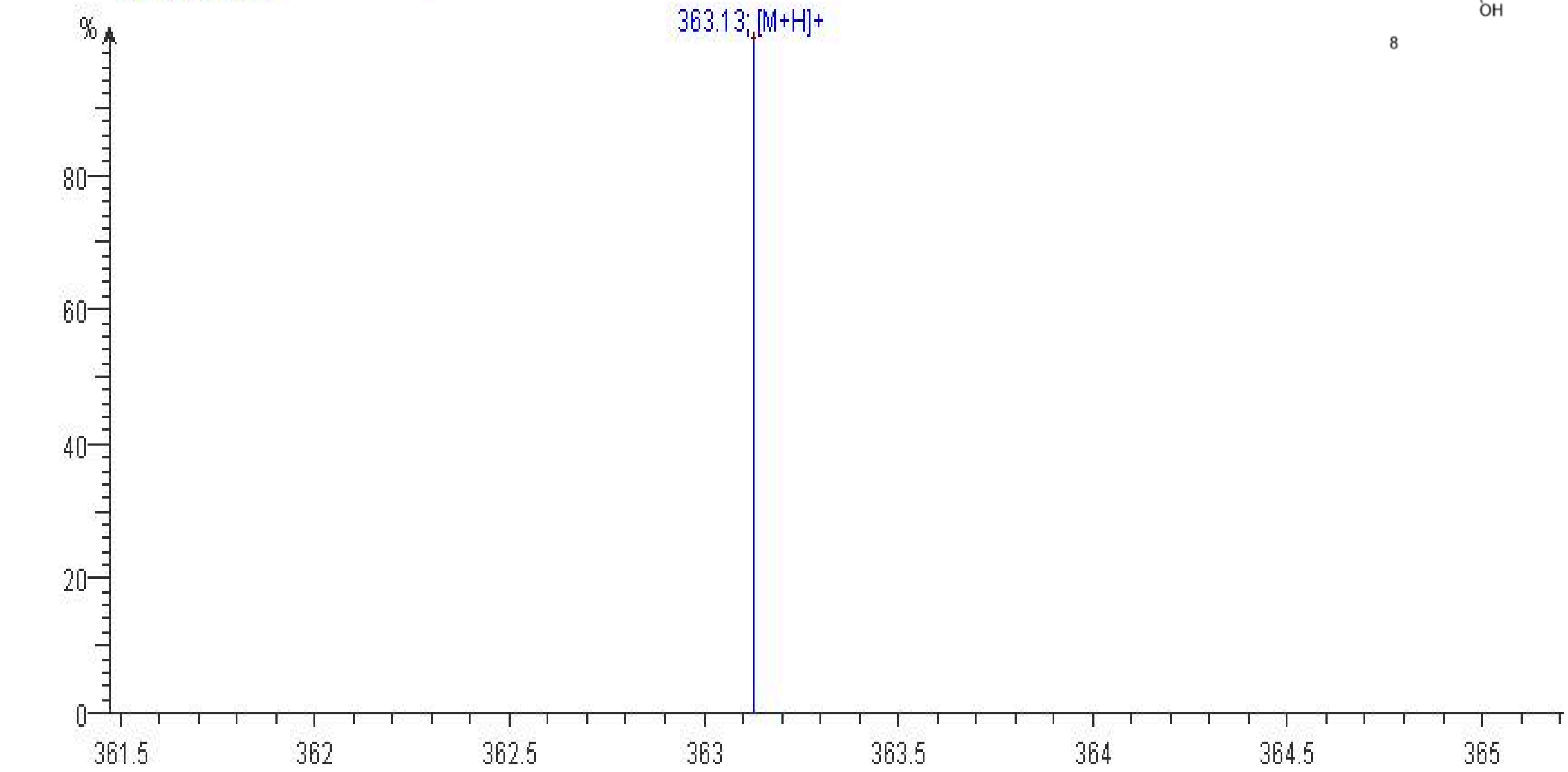

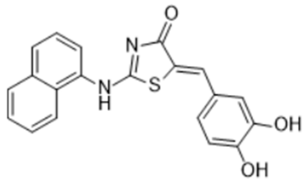

8

Figure S25. LRMS (ESI+) spectrum of analog **8**

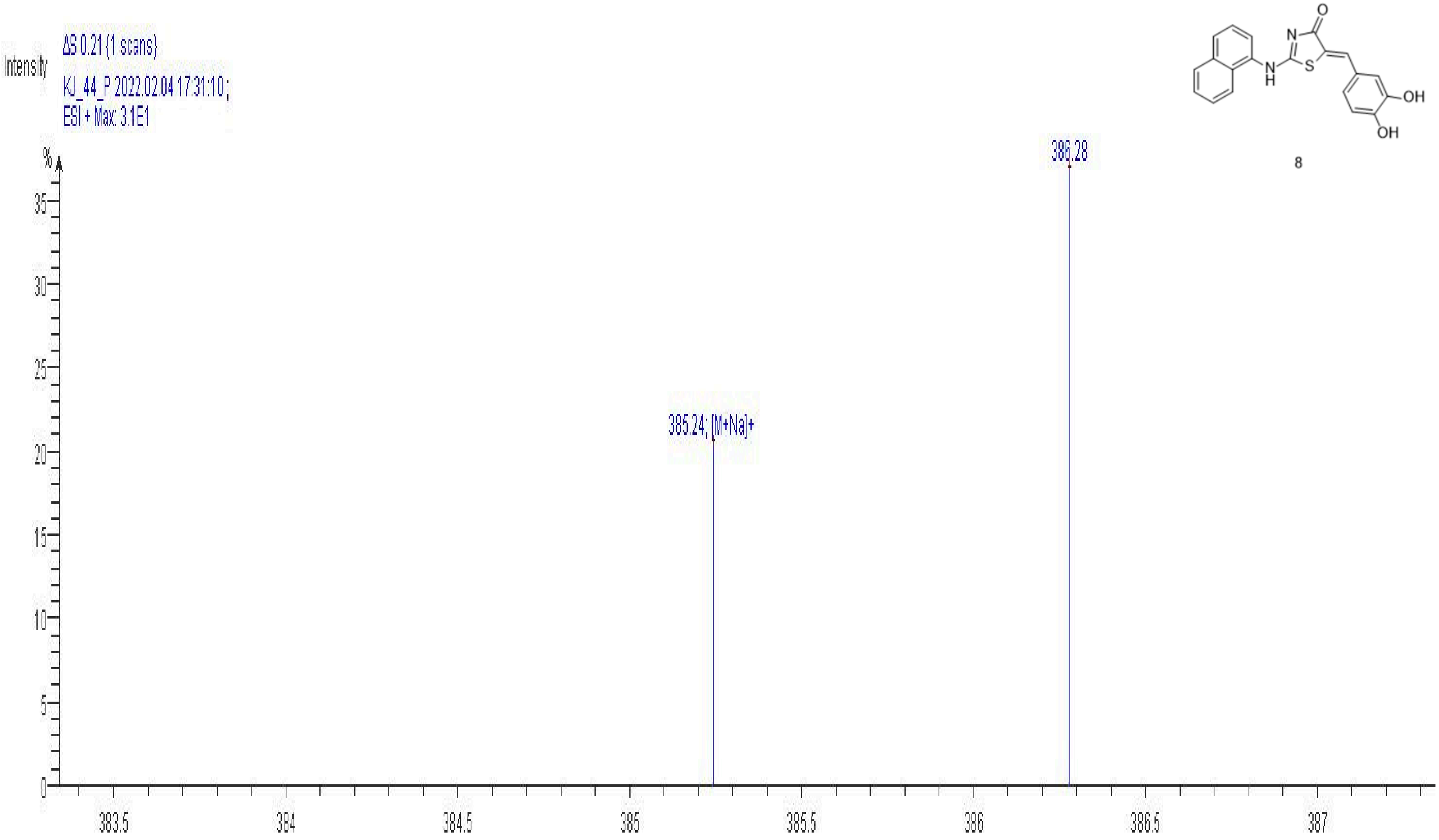

8

Figure S26. LRMS (ESI+) spectrum of analog **8**

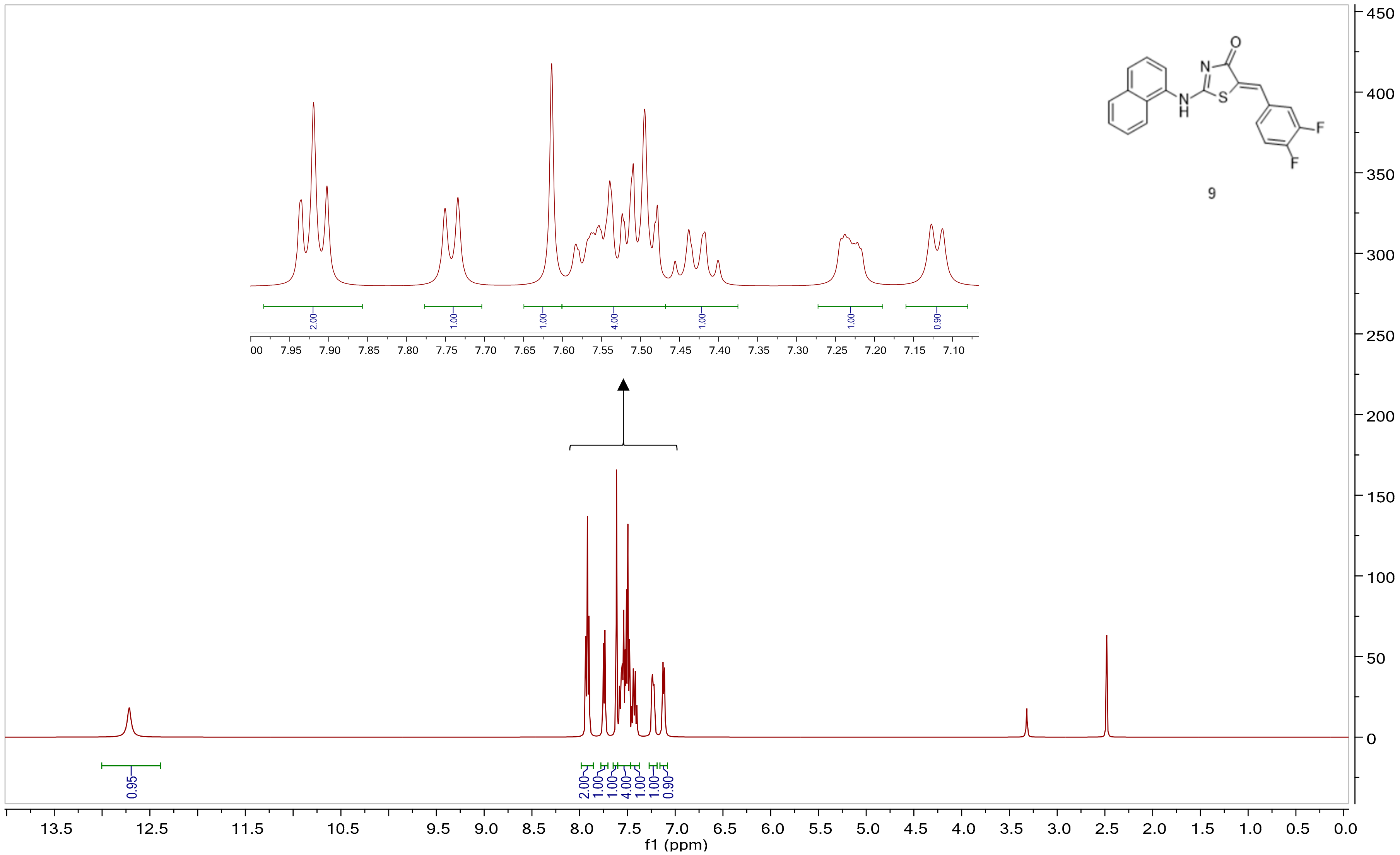

Figure S27. ^{1}H NMR spectrum of analog **9**

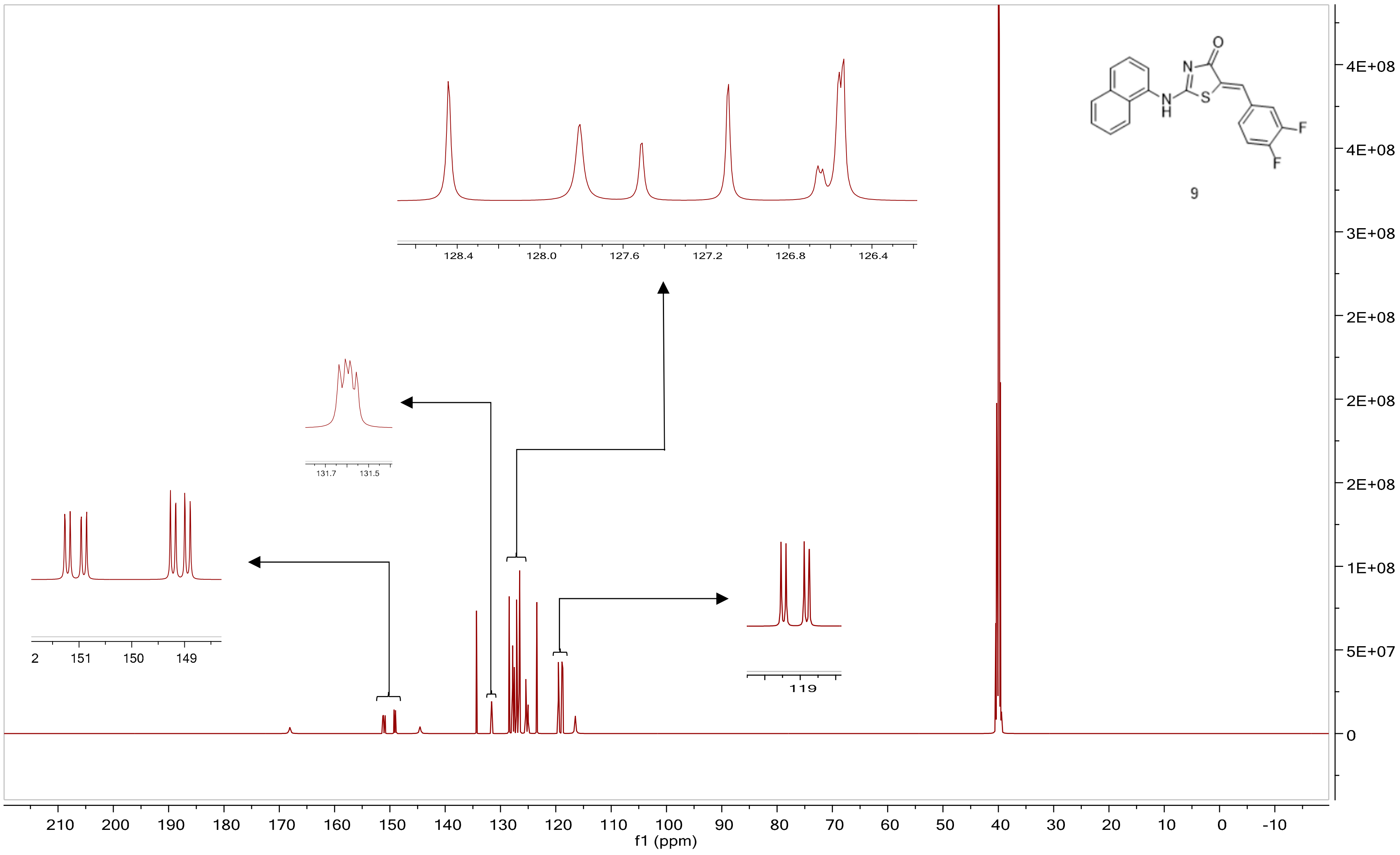

Figure S28. ^{13}C NMR spectrum of analog **9**

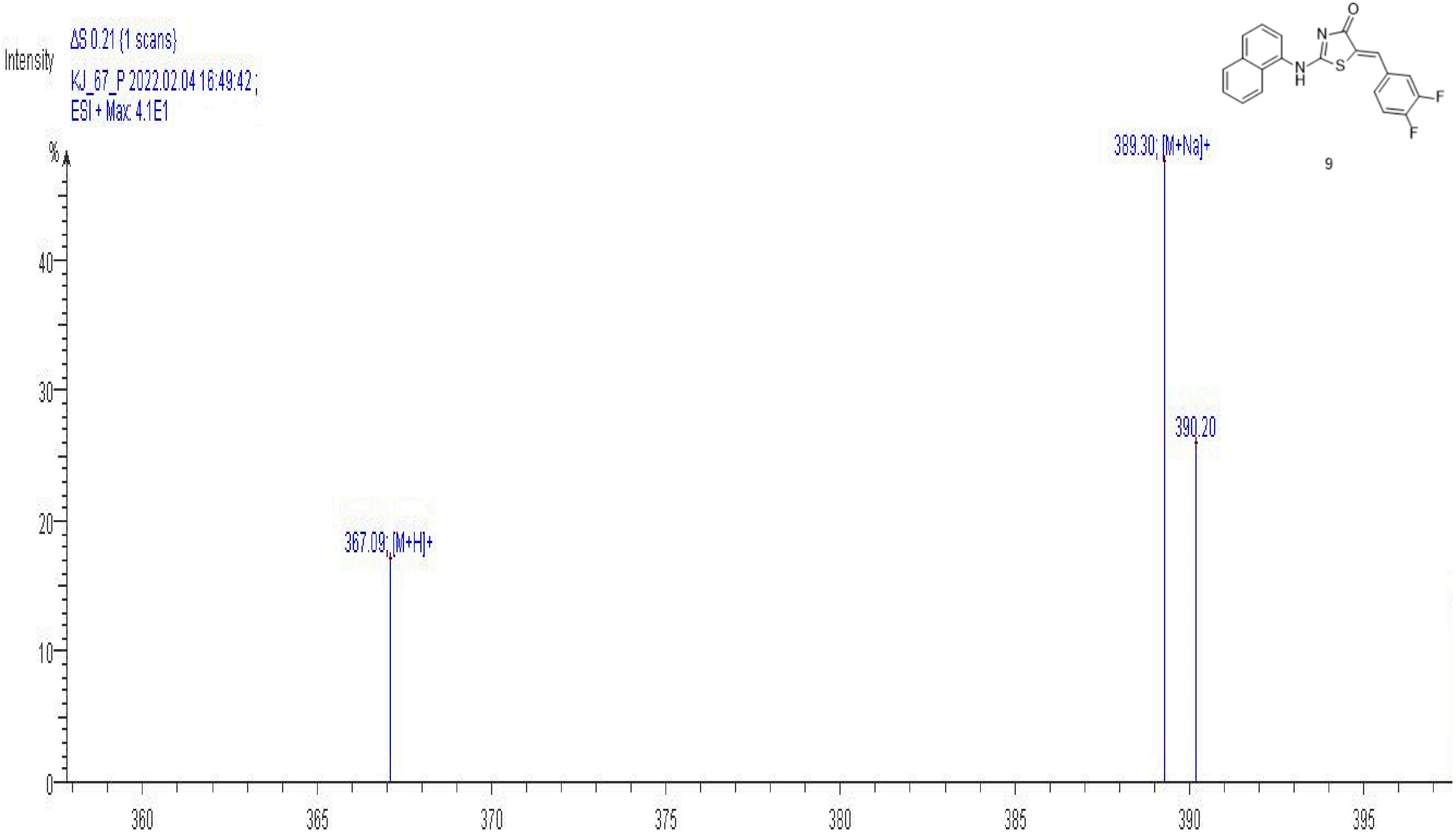

Figure S29. LRMS (ESI+) spectrum of analog **9**

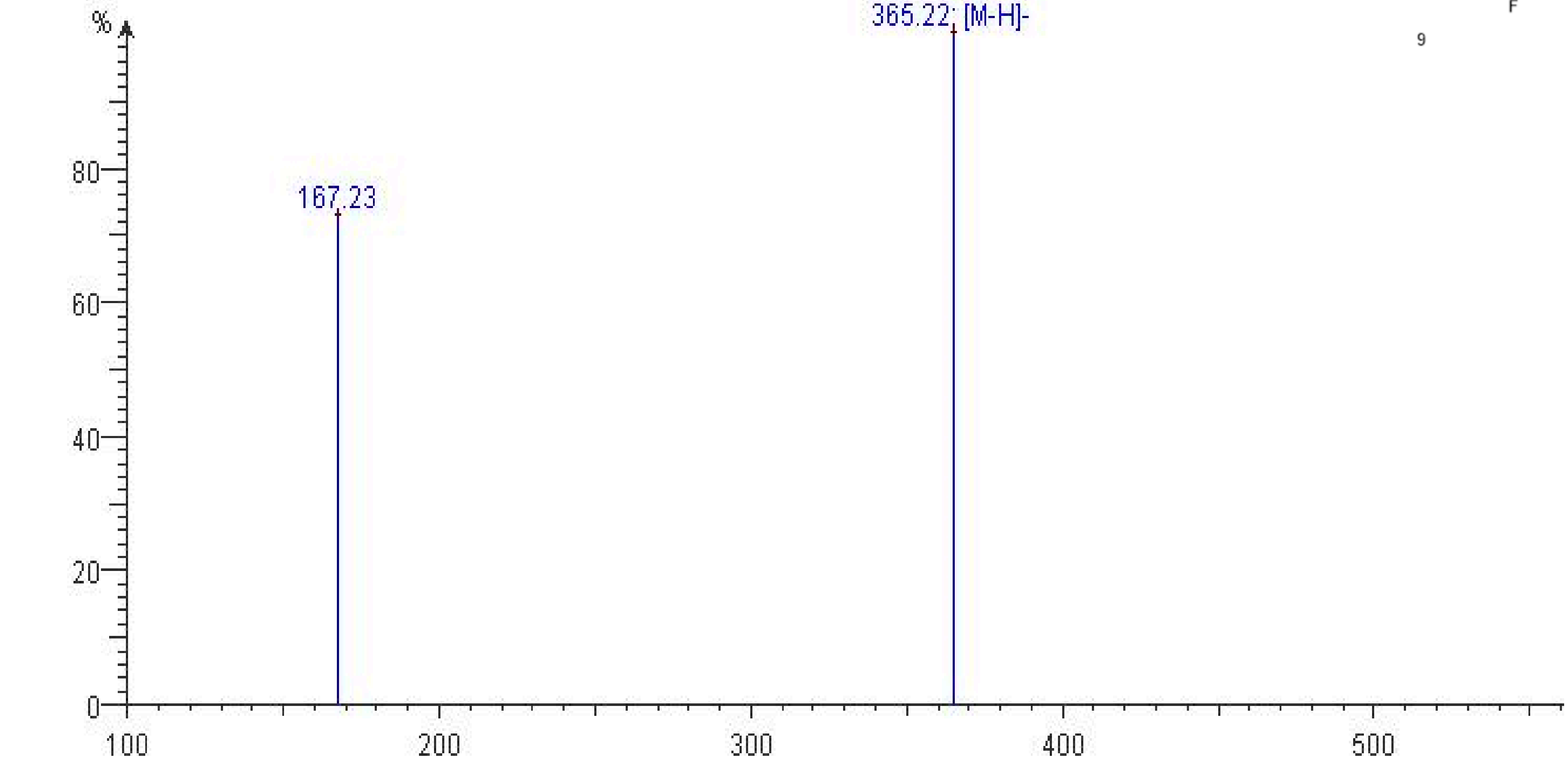

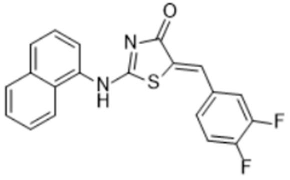

9

Figure S30. LRMS (ESI–) spectrum of analog **9**

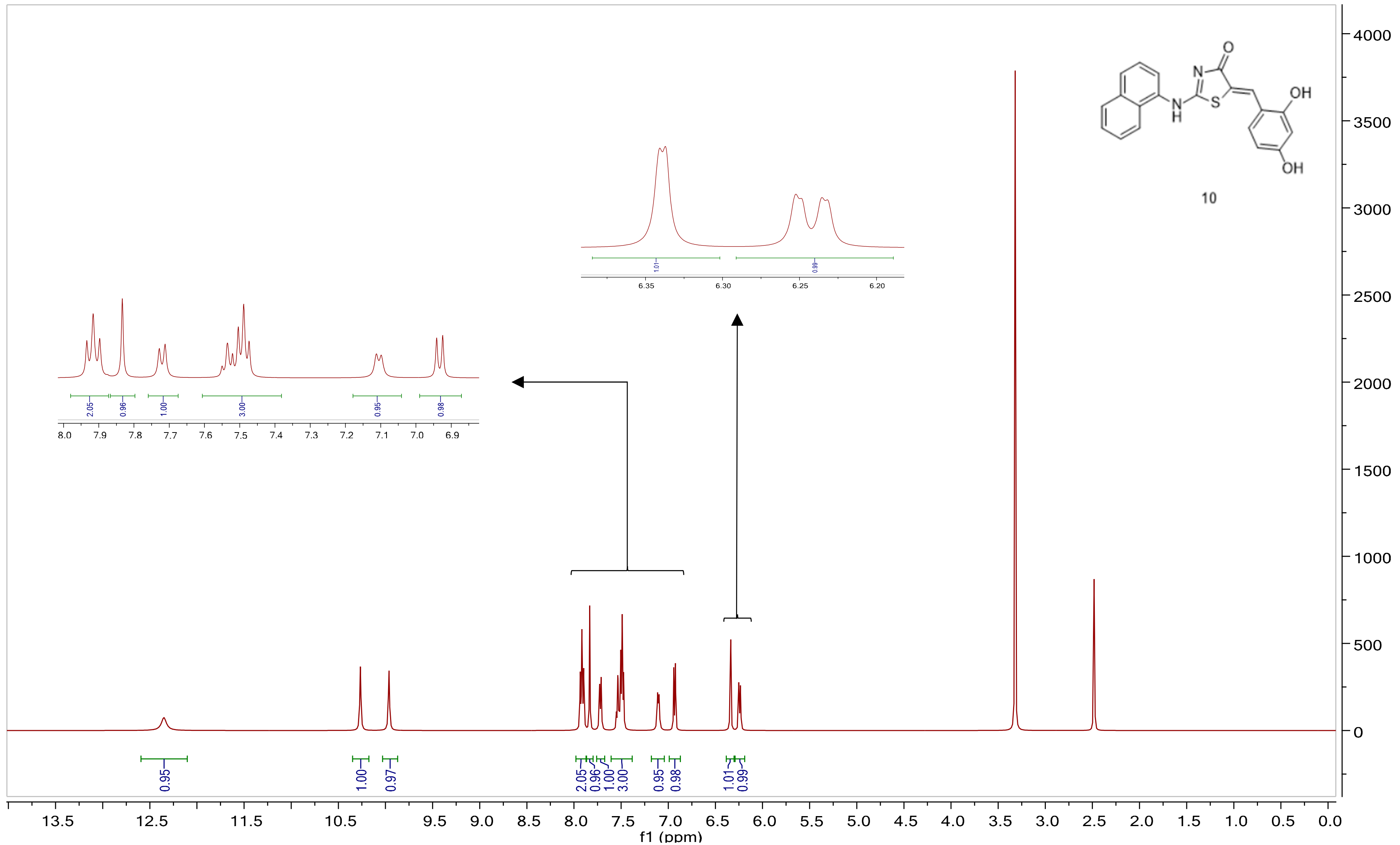

Figure S31. ^{1}H NMR spectrum of analog **10**

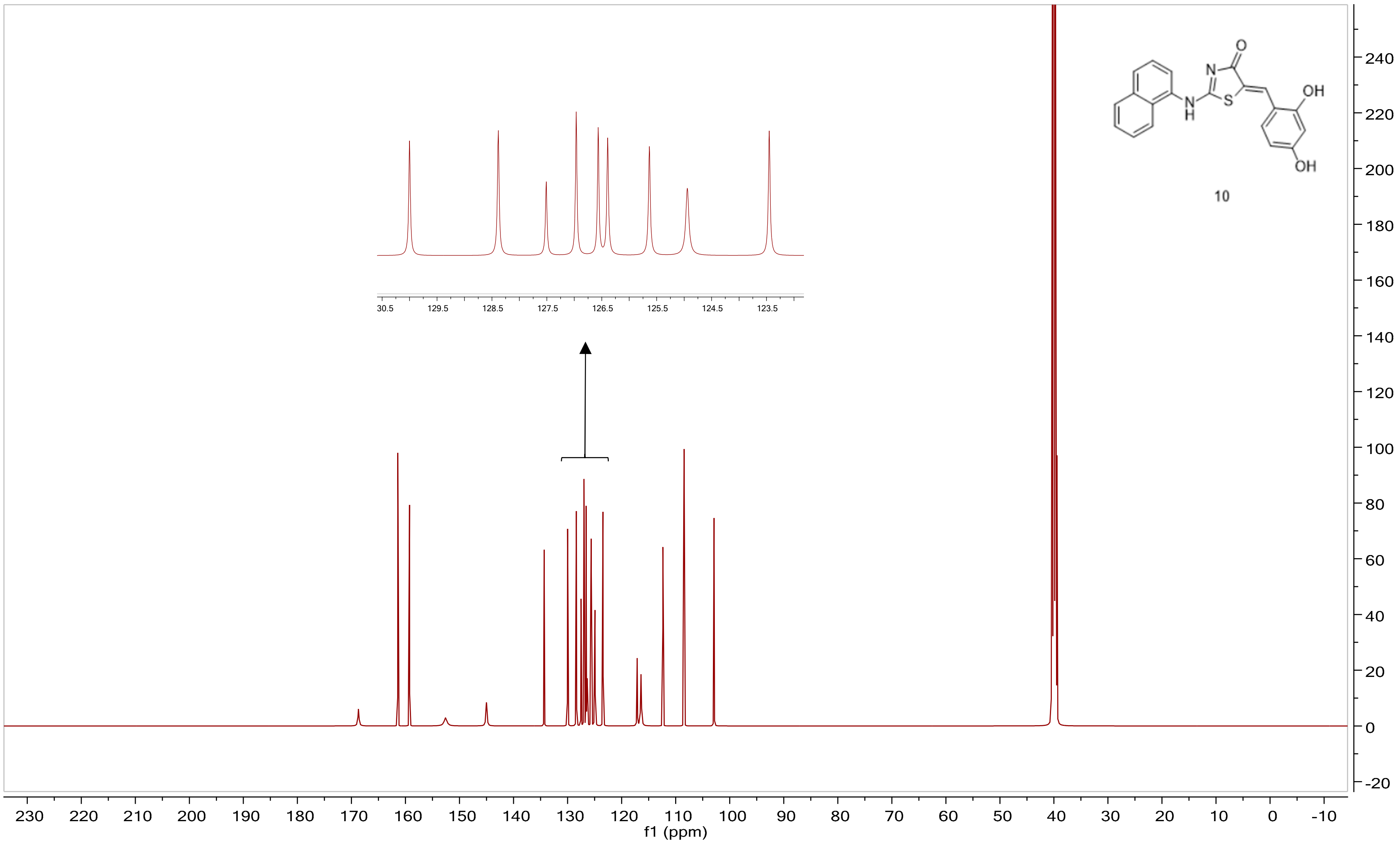

Figure S32. ^{13}C NMR spectrum of analog **10**

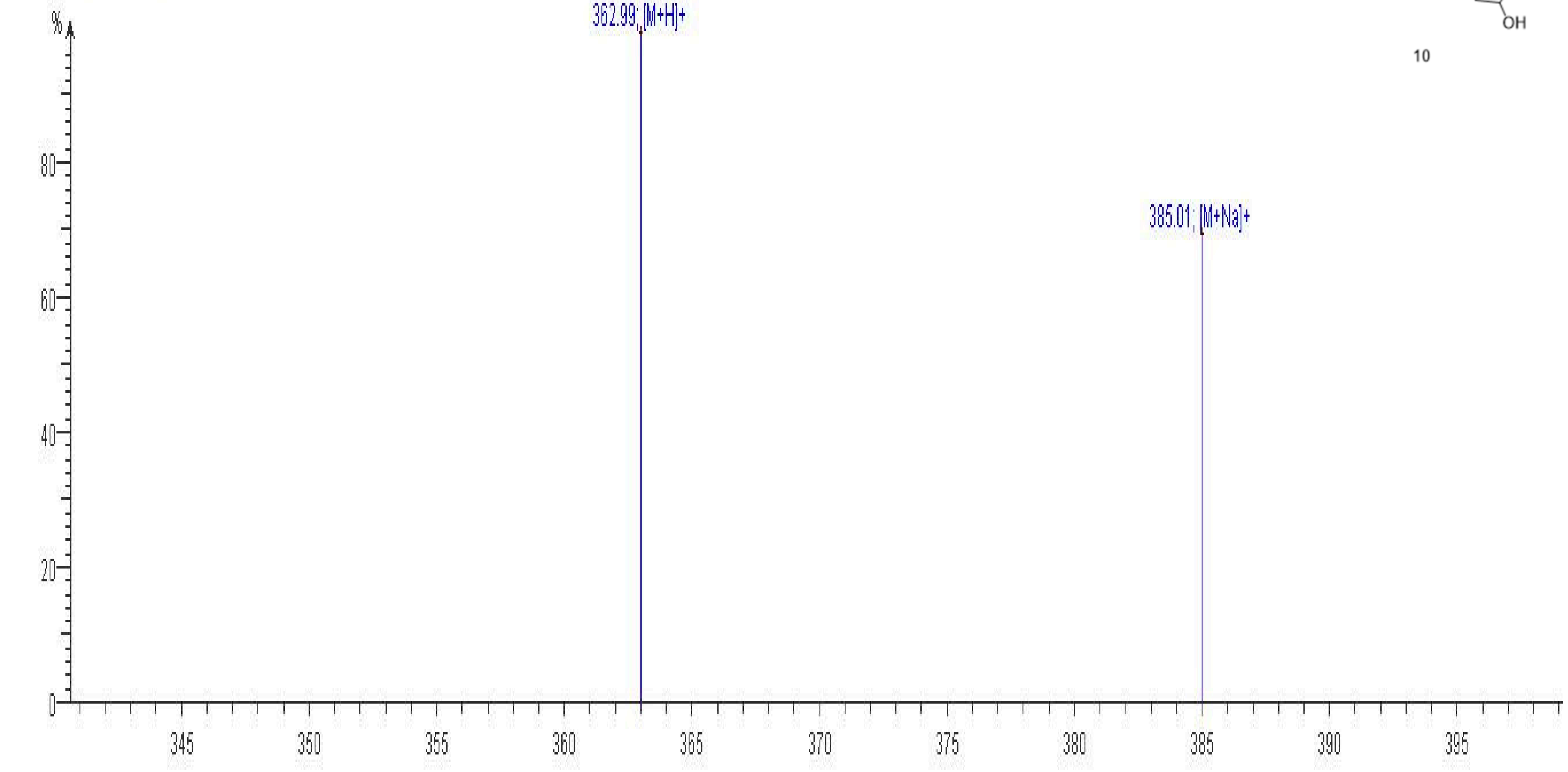

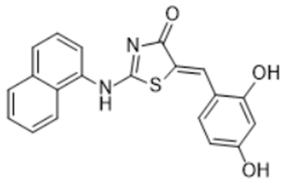

10

Figure S33. LRMS (ESI+) spectrum of analog **10**

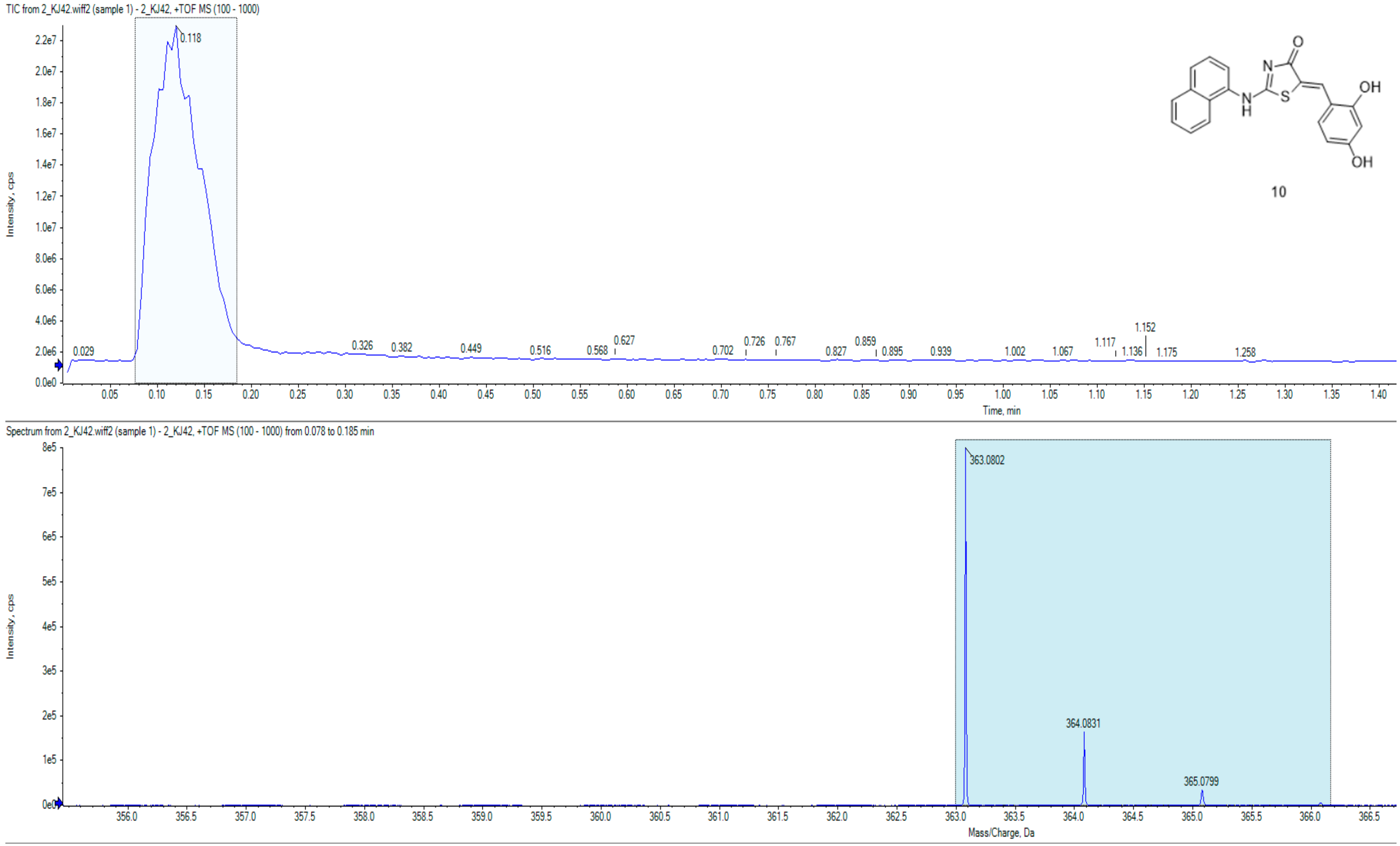

Figure S34-1. HRMS (ESI+) spectrum of analog **10**

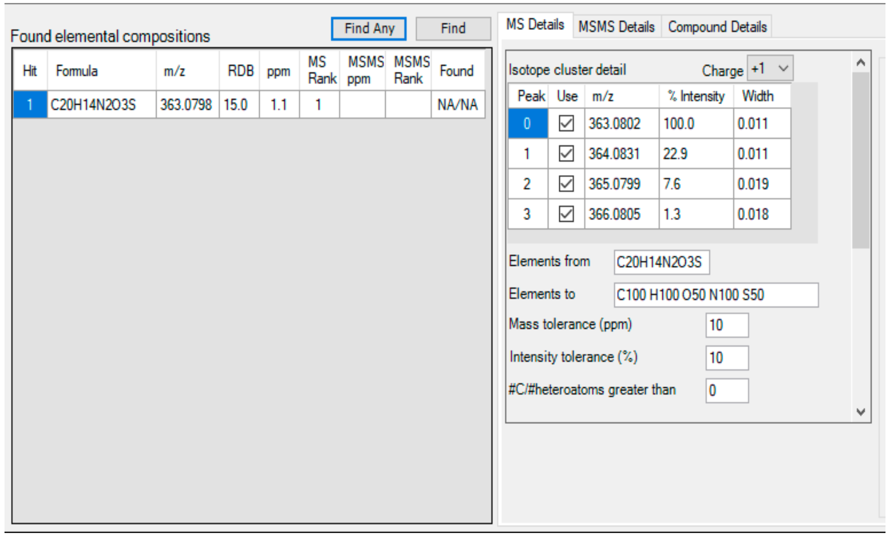

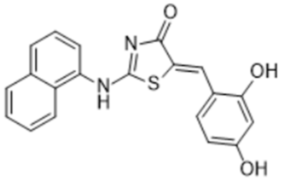

10

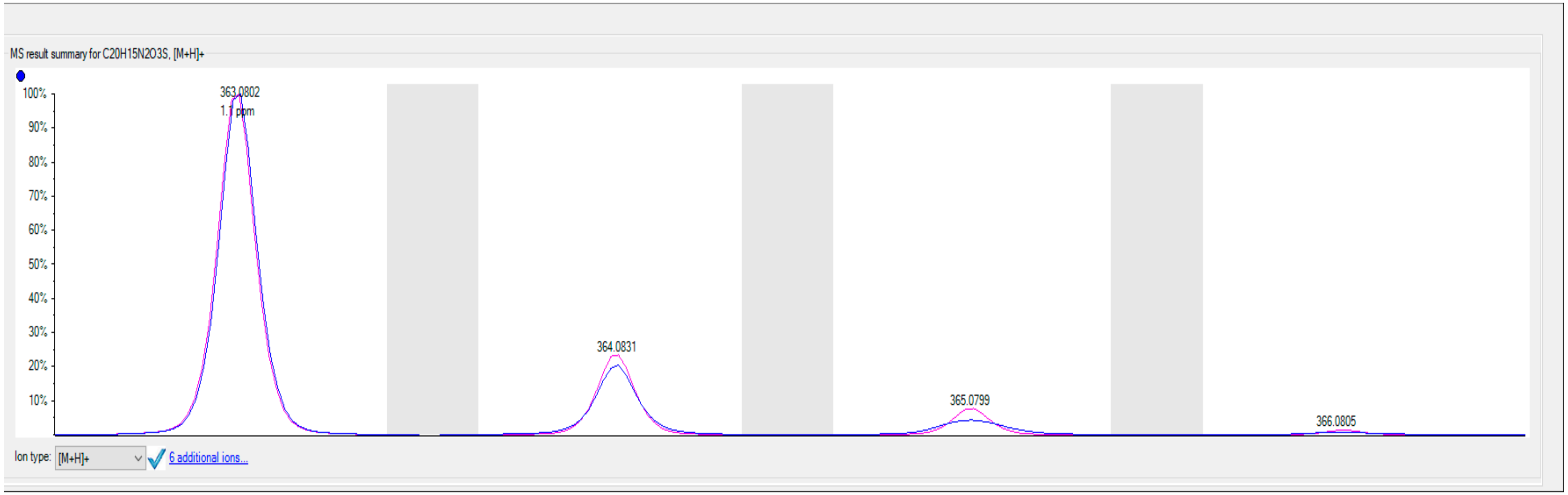

Figure S34-2. HRMS (ESI+) spectrum of analog **10**

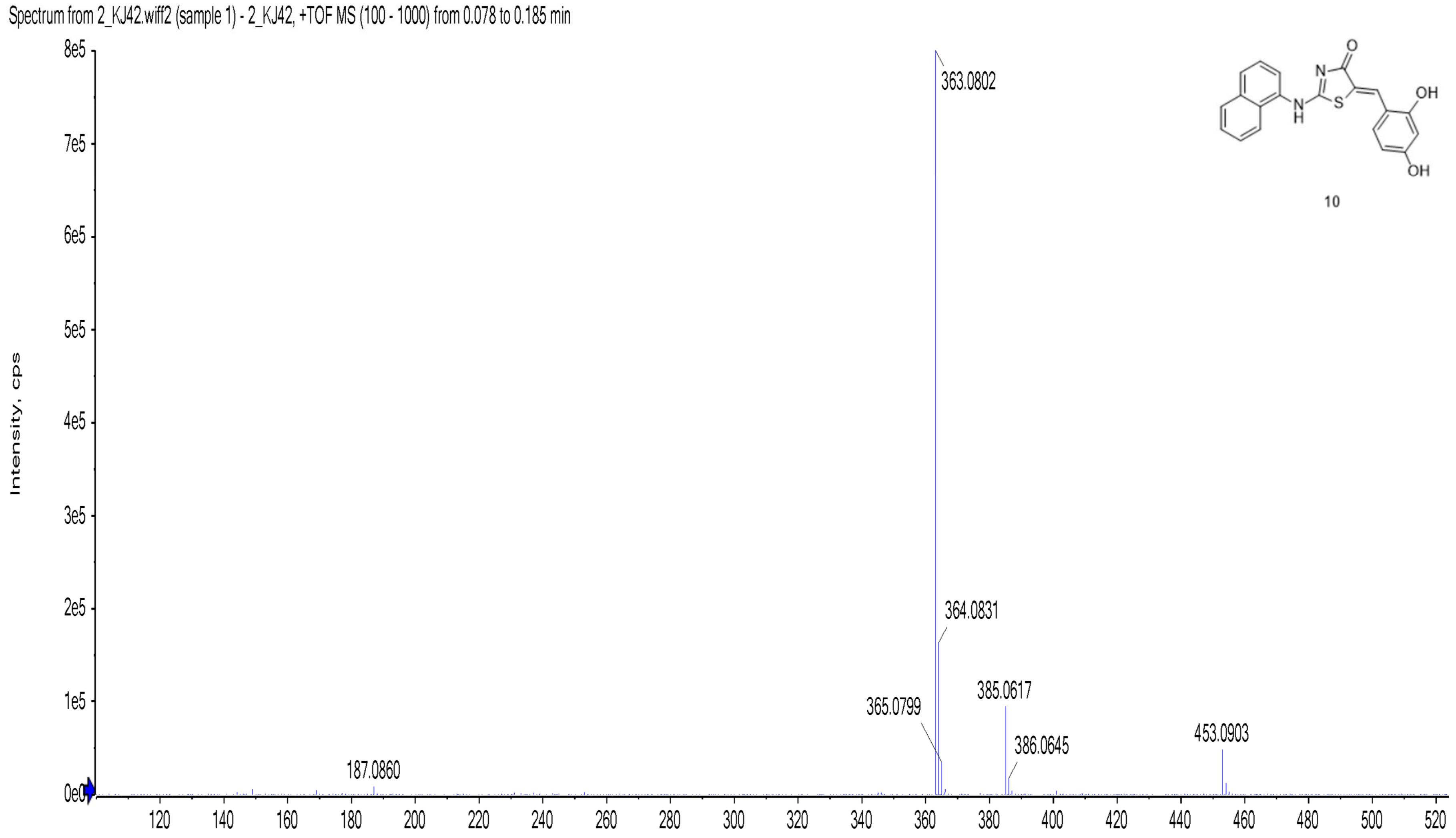

Figure S34-3. HRMS (ESI+) spectrum of analog **10**

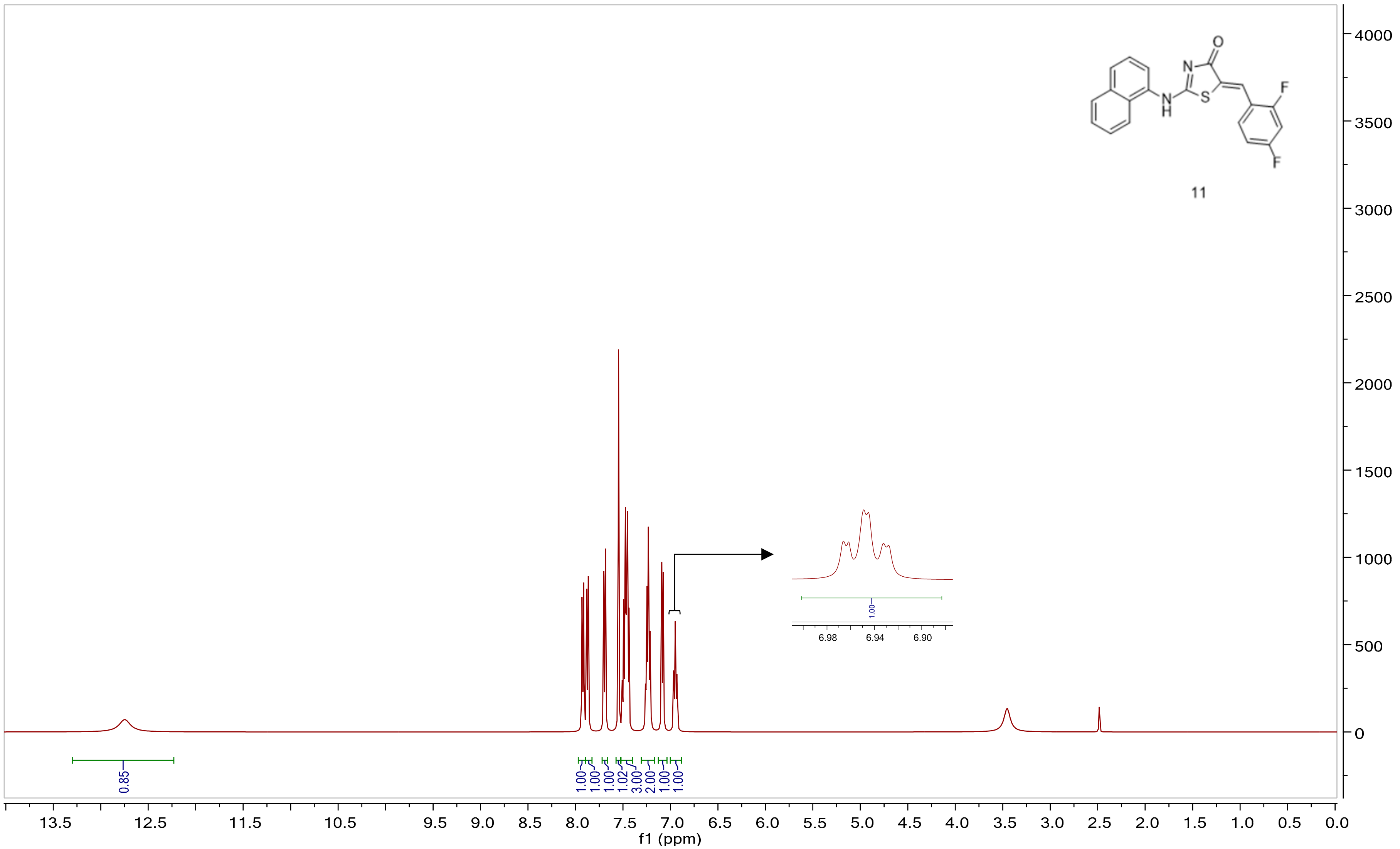

Figure S35. ^{1}H NMR spectrum of analog **11**

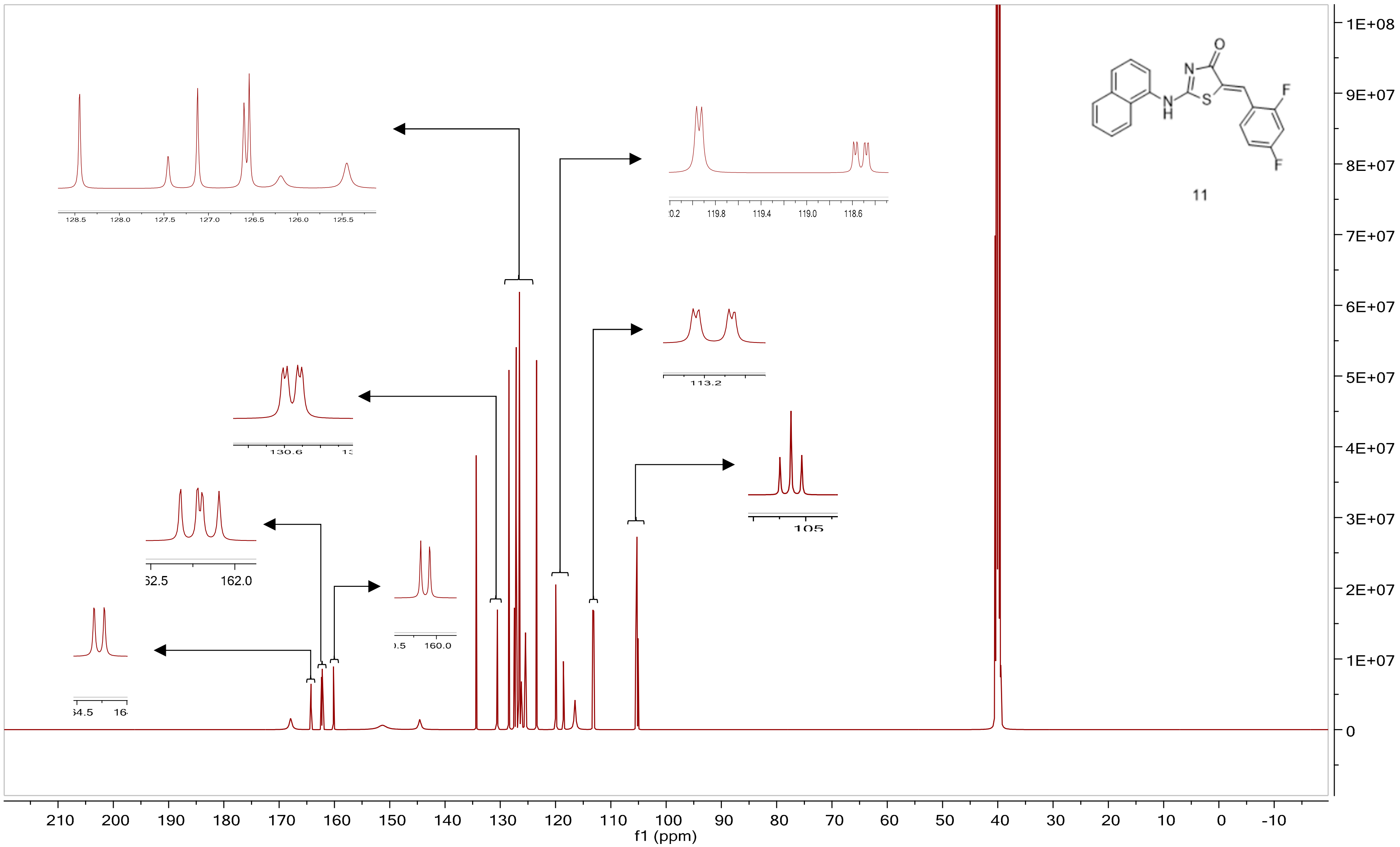

Figure S36. ^{13}C NMR spectrum of analog **11**

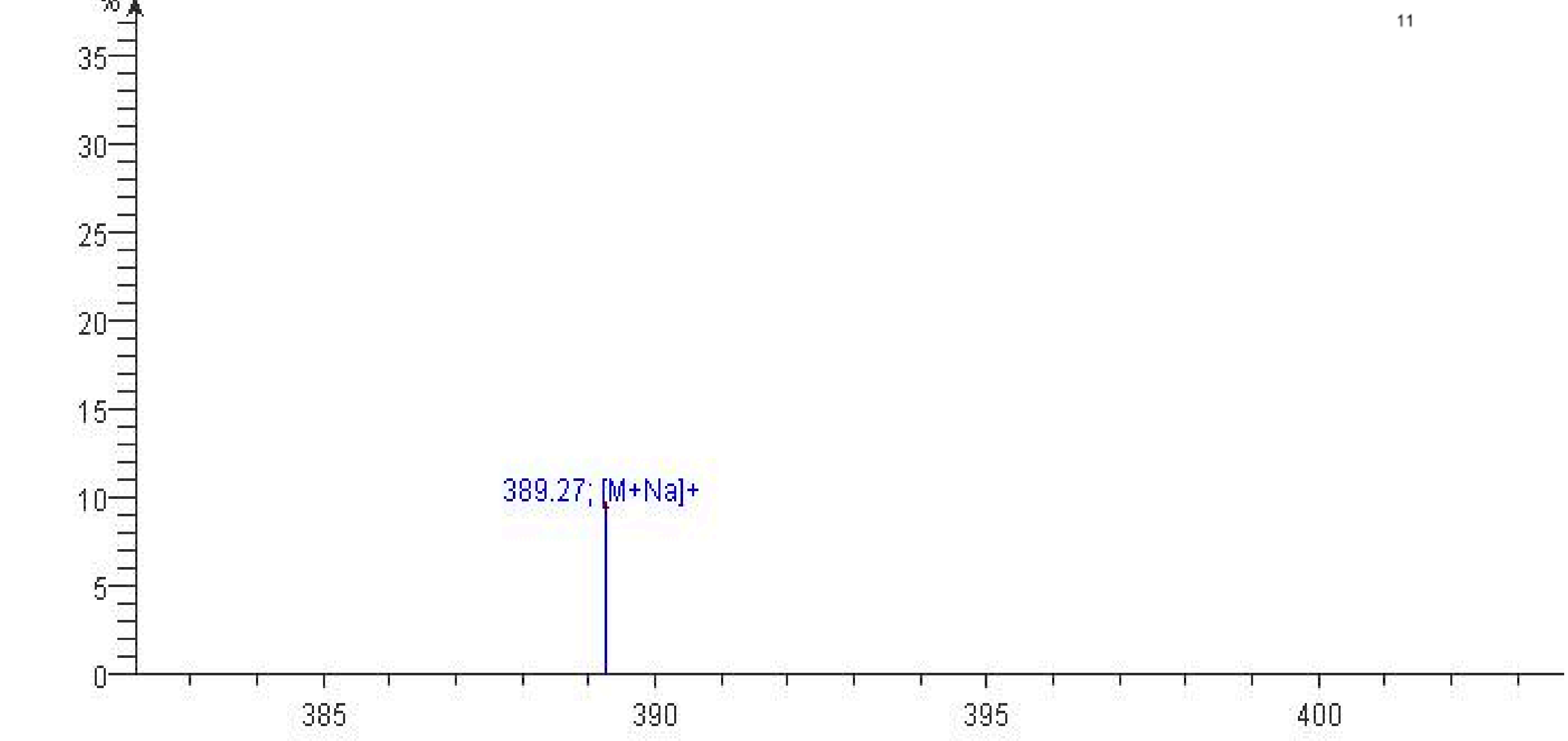

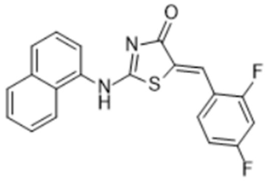

11

Figure S37. LRMS (ESI+) spectrum of analog **11**

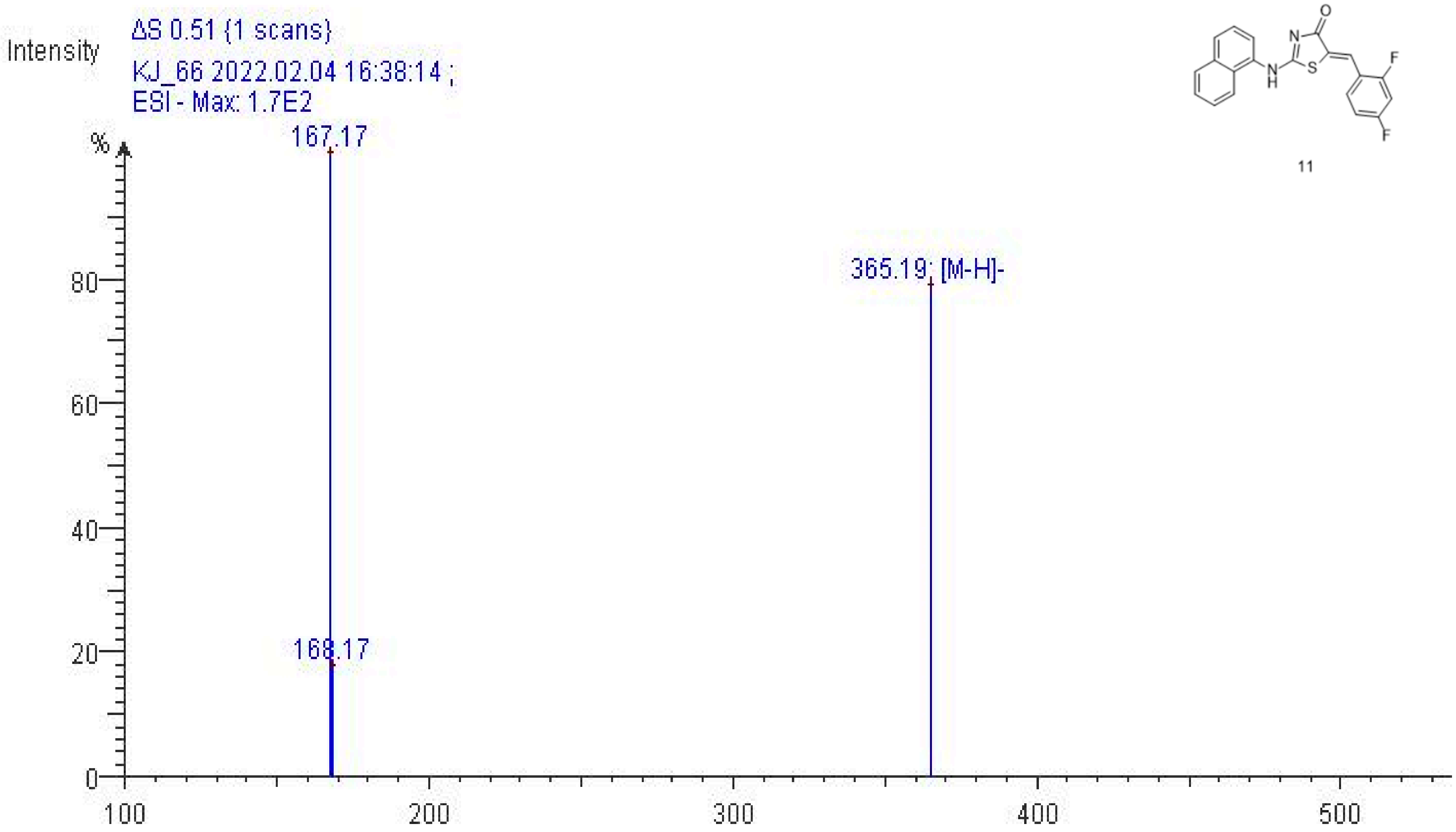

Figure S38. LRMS (ESI–) spectrum of analog **11**

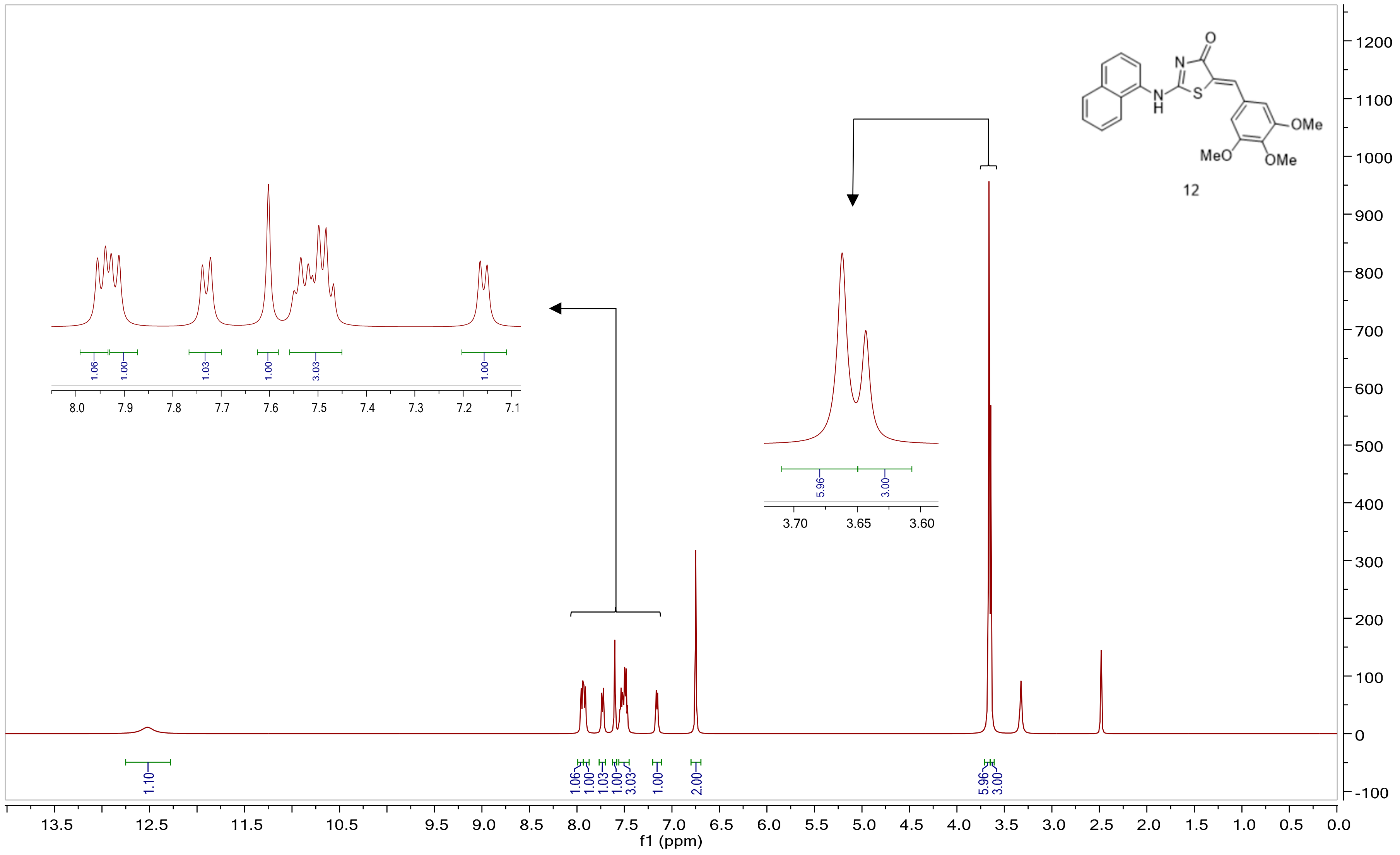

Figure S39. ^{1}H NMR spectrum of analog **12**

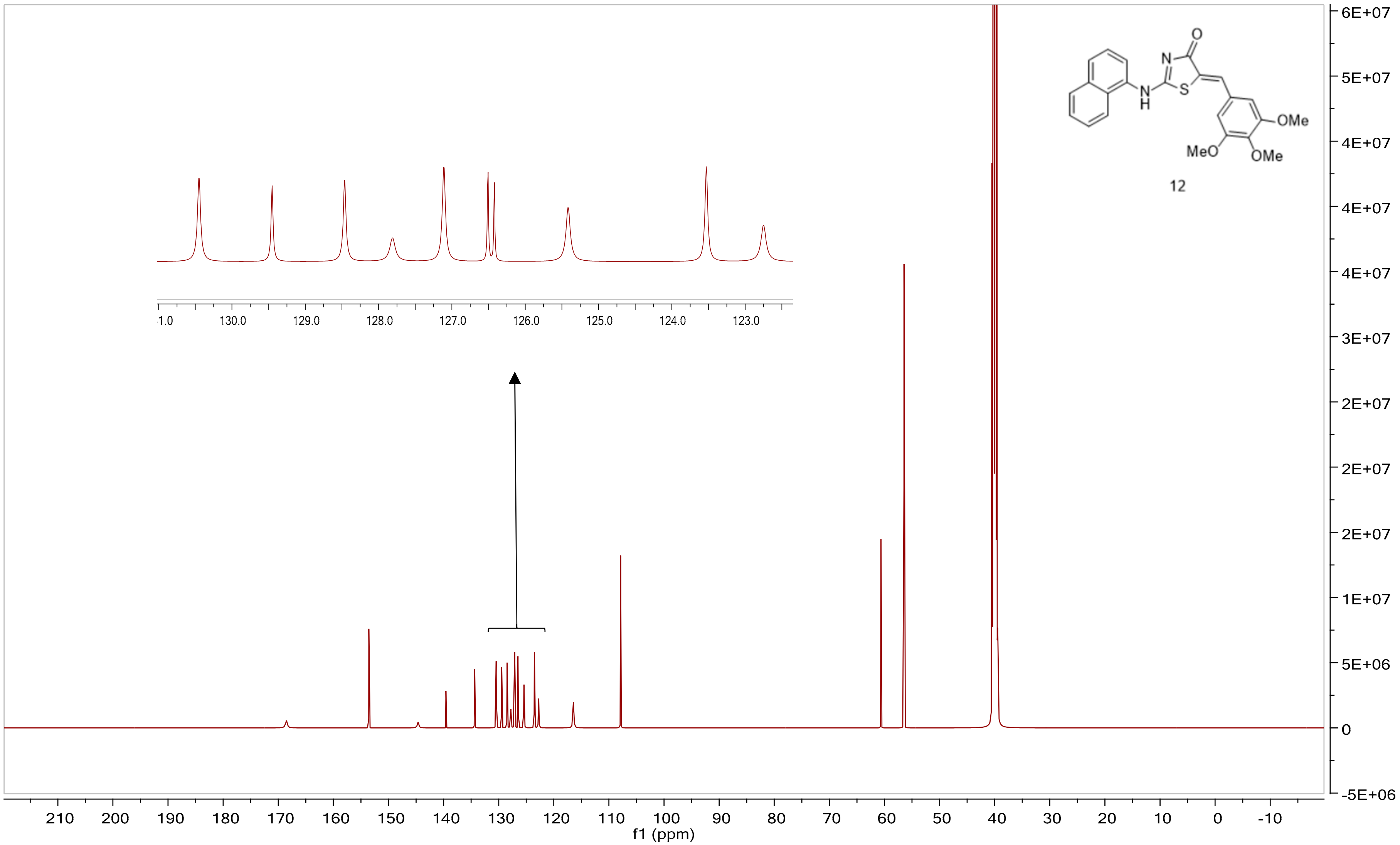

Figure S40. ^{13}C NMR spectrum of analog **12**

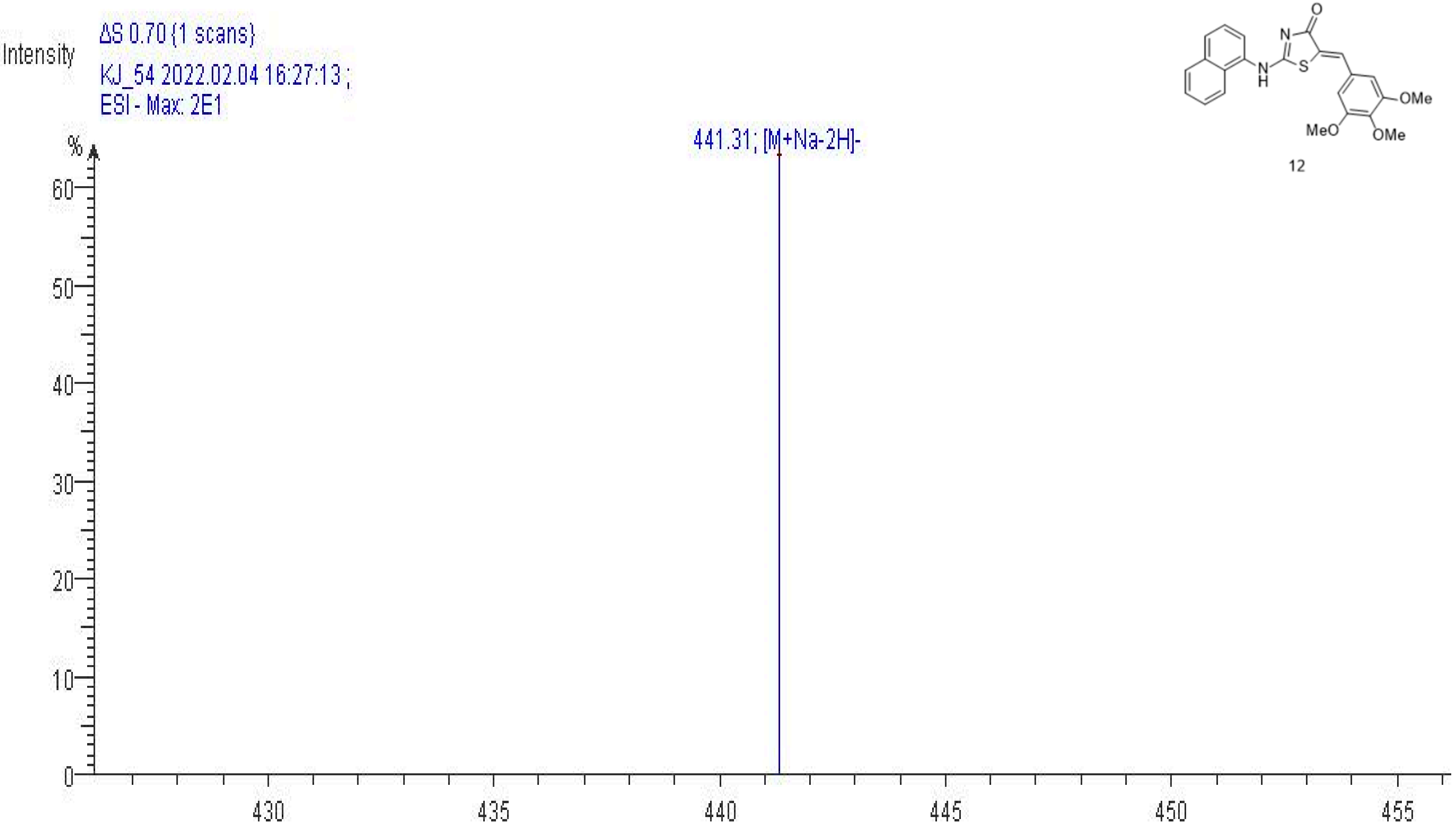

Figure S41. LRMS (ESI–) spectrum of analog **12**

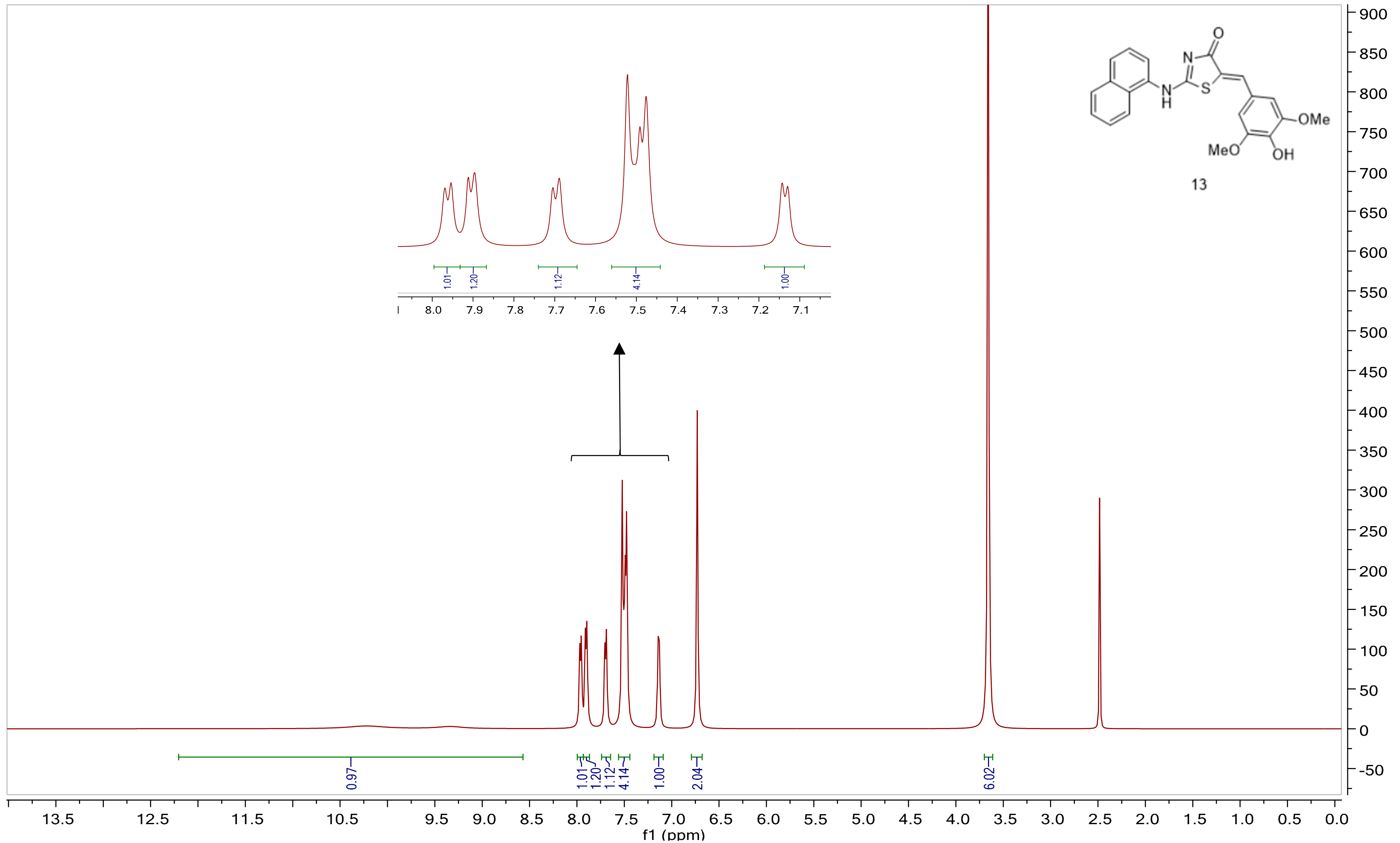

Figure S42. ^{1}H NMR spectrum of analog **13**

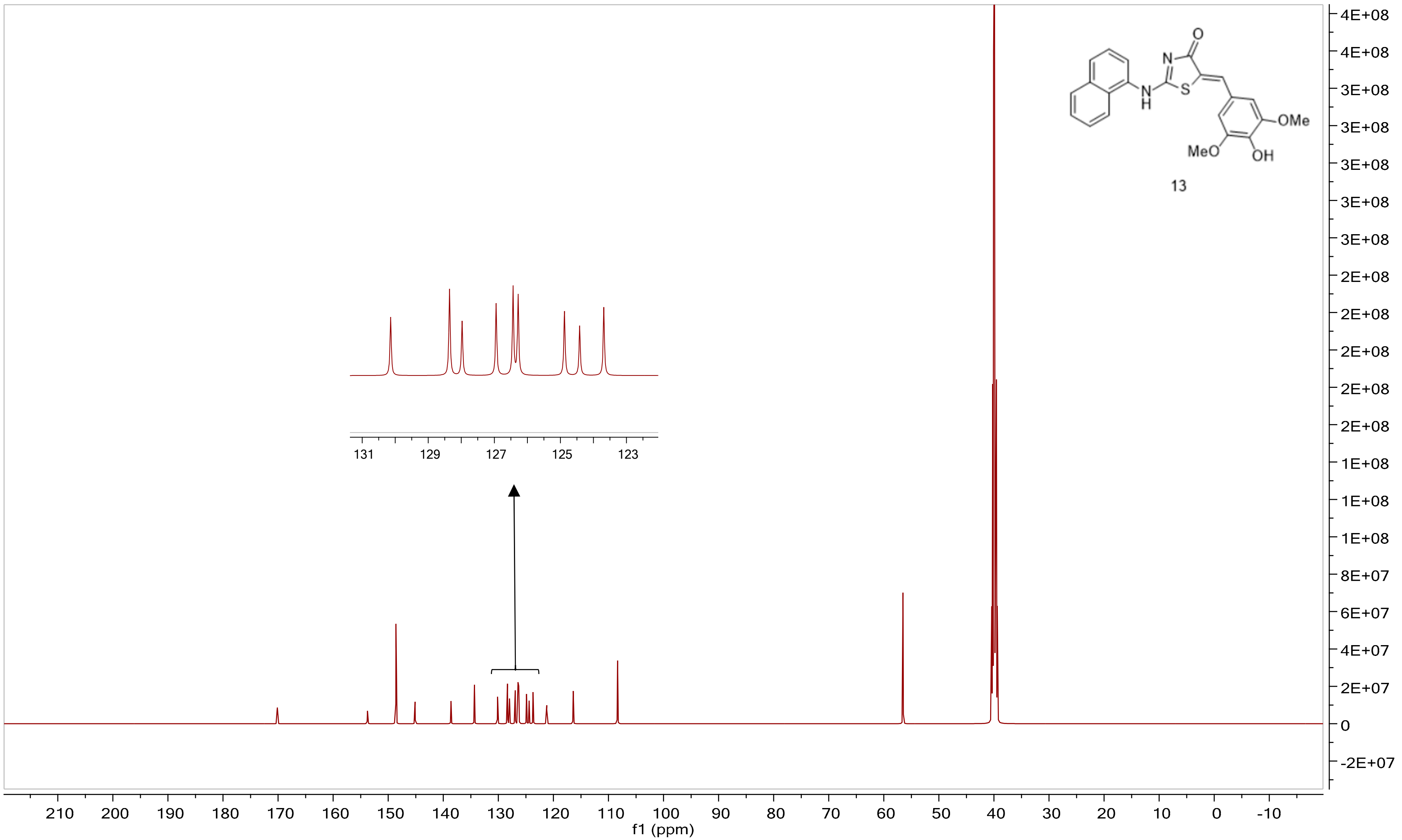

Figure S43. ^{13}C NMR spectrum of analog **13**

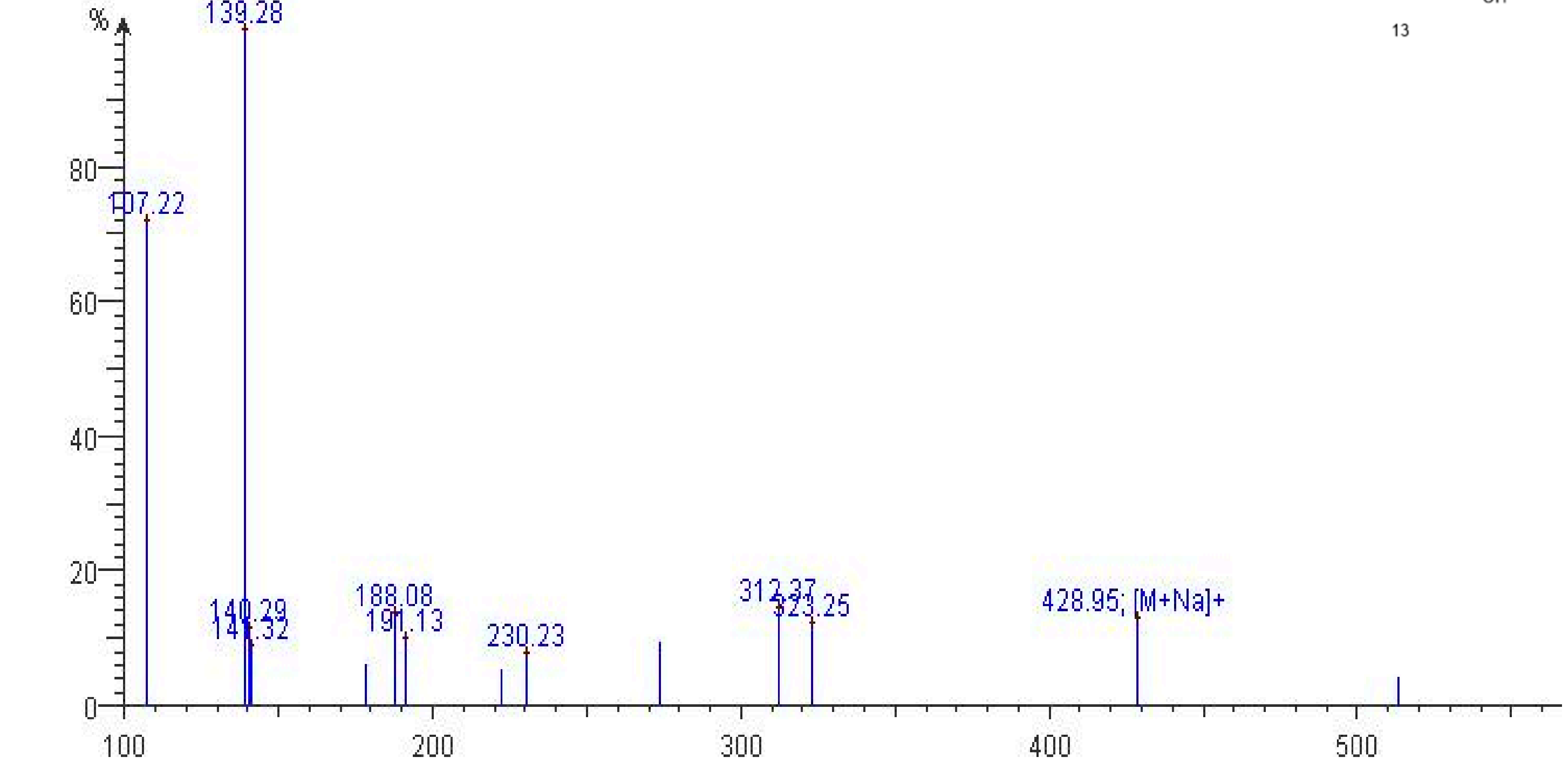

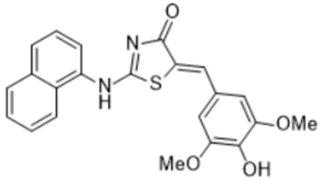

13

Figure S44. LRMS (ESI+) spectrum of analog **13**

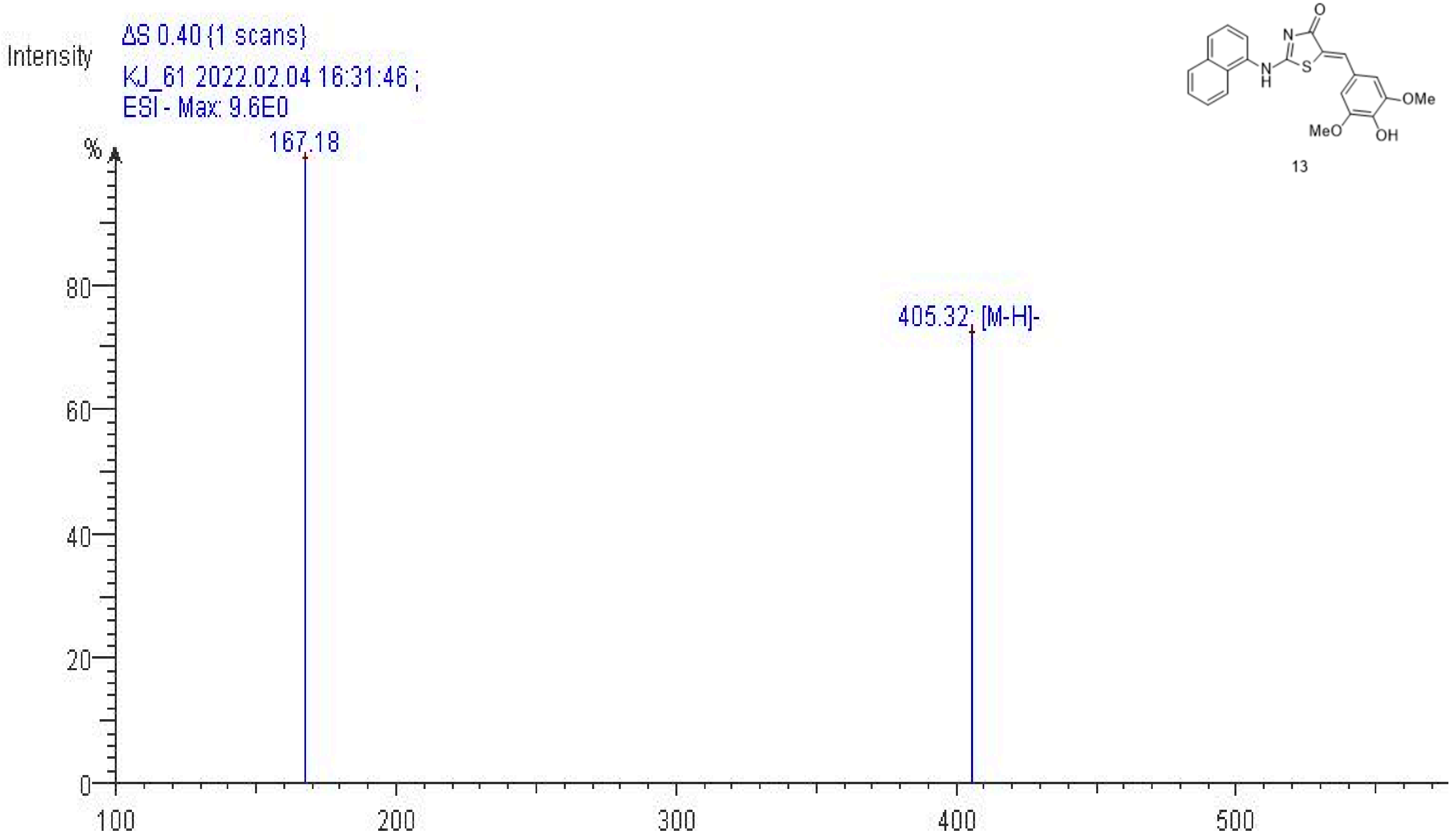

13

Figure S45. LRMS (ESI–) spectrum of analog **13**

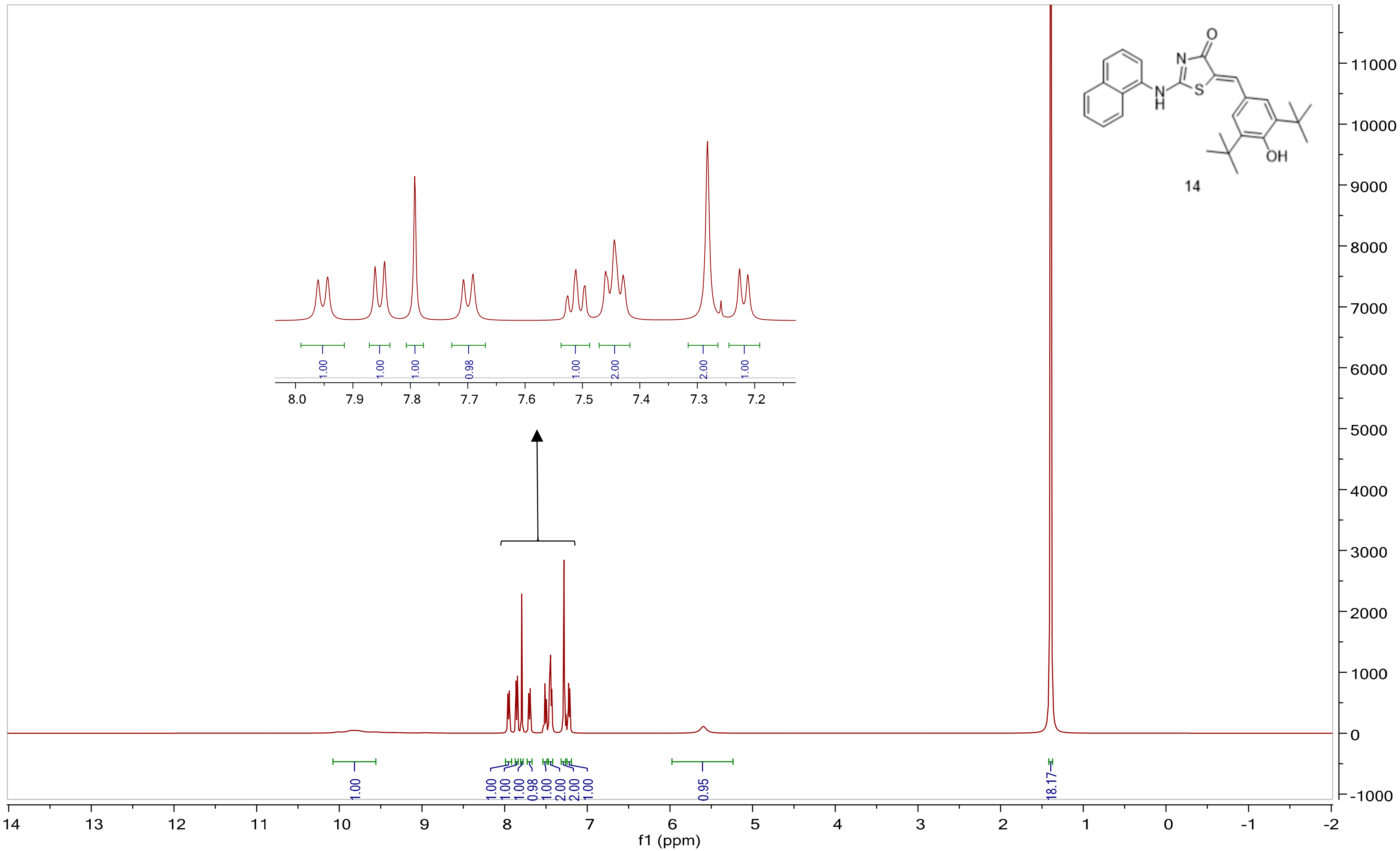

Figure S46. ^{1}H NMR spectrum of analog **14**

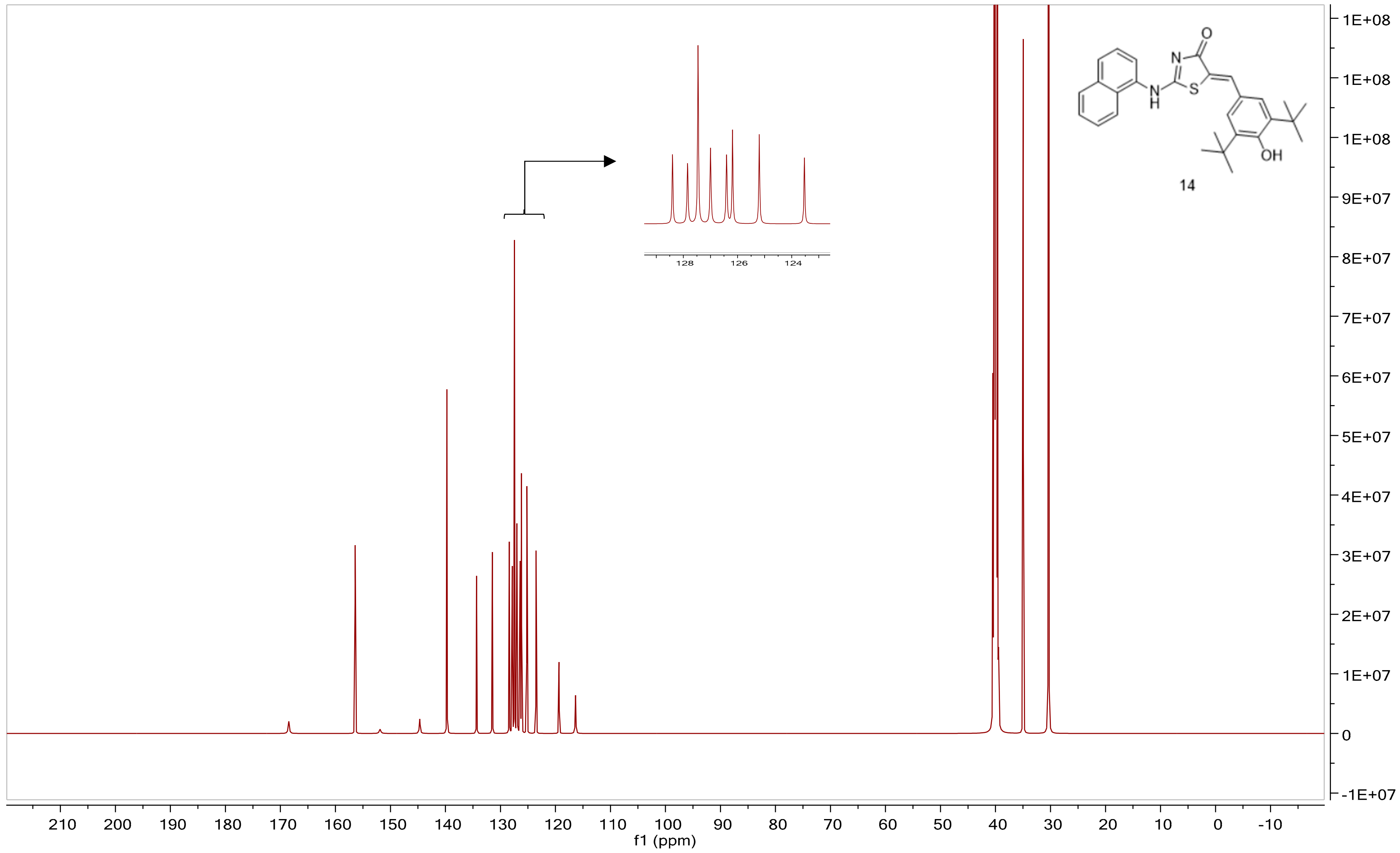

Figure S47. ^{13}C NMR spectrum of analog **14**

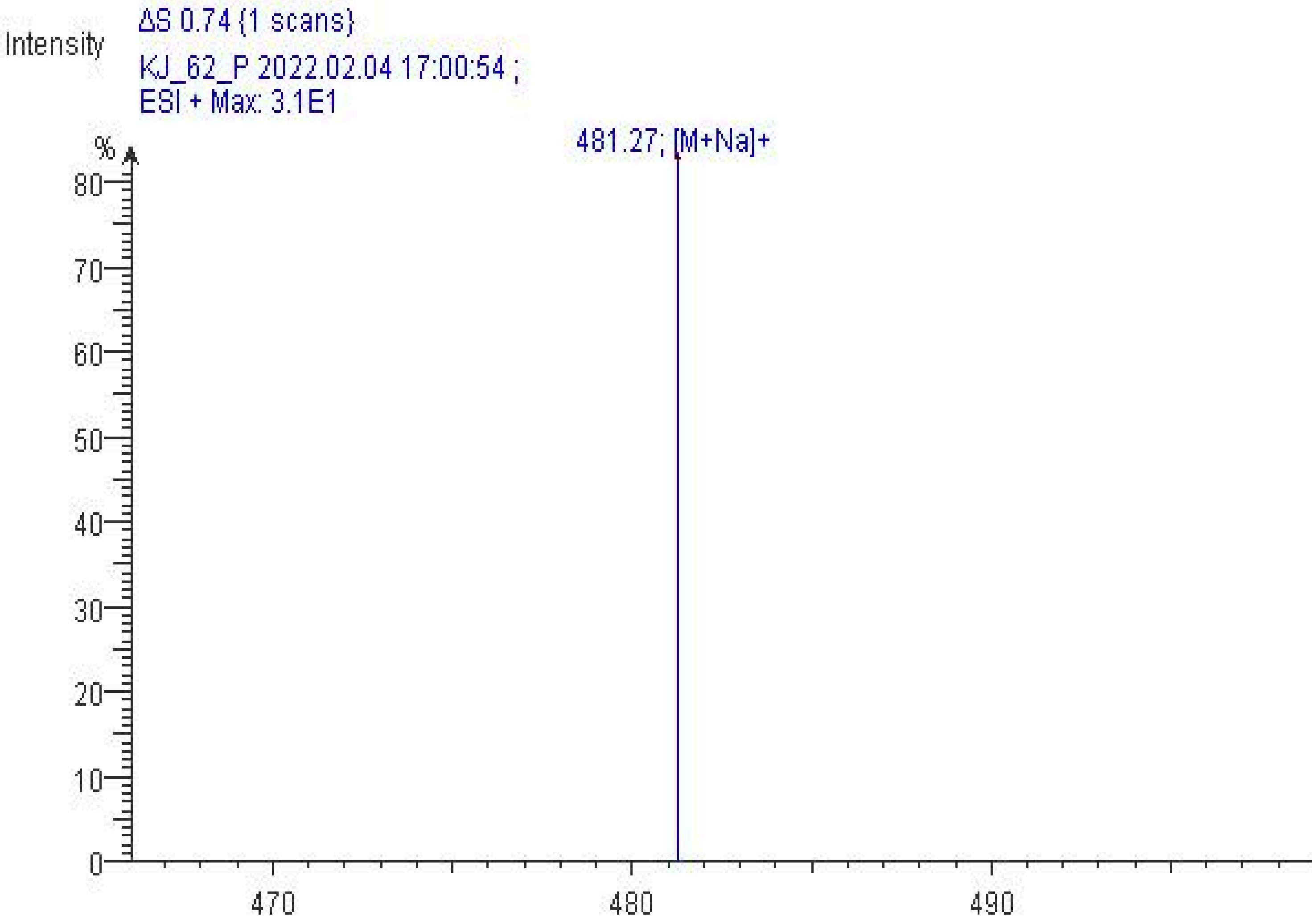

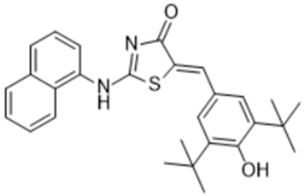

14

Figure S48. LRMS (ESI+) spectrum of analog **14**

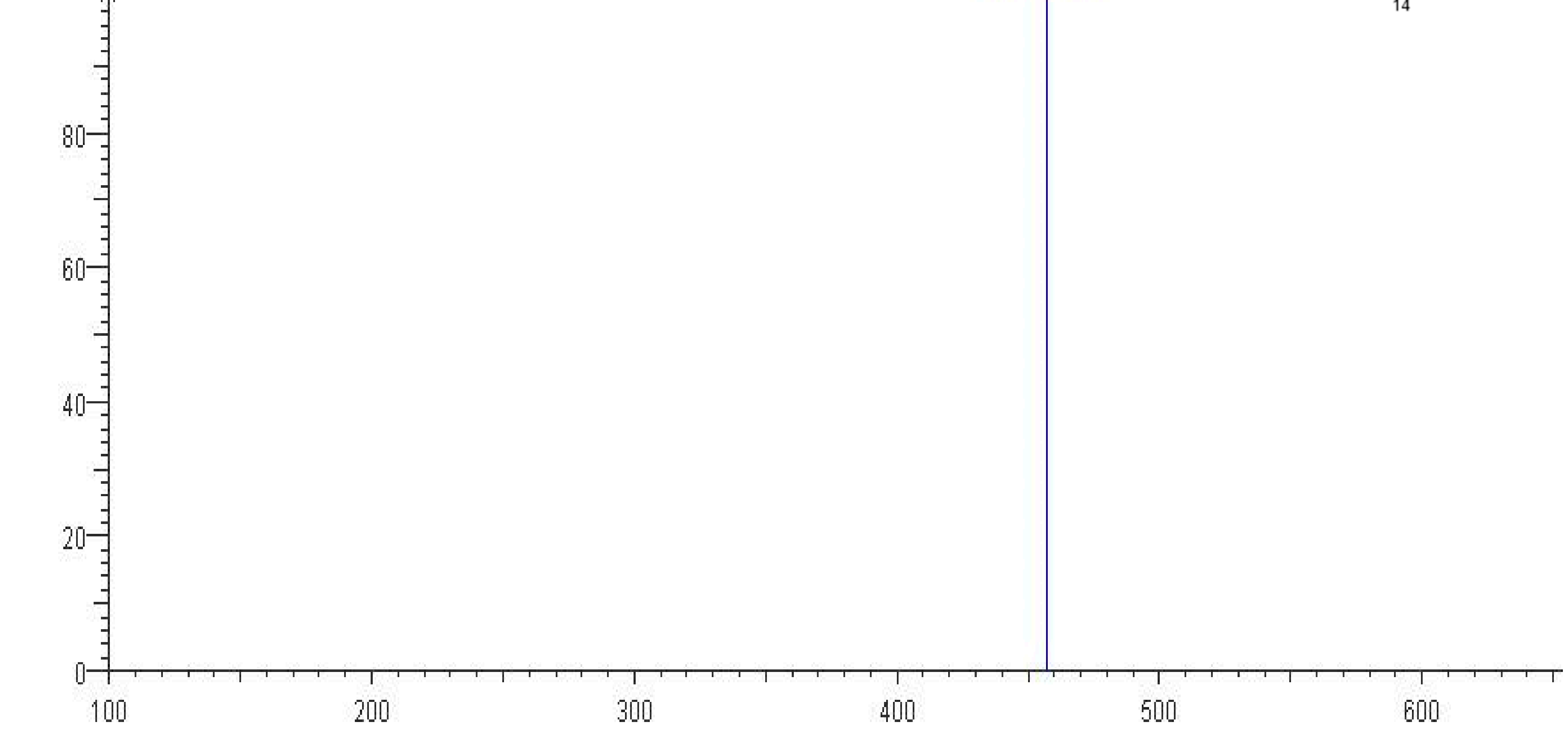

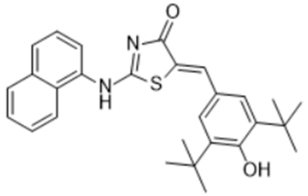

14

Figure S49. LRMS (ESI–) spectrum of analog **14**

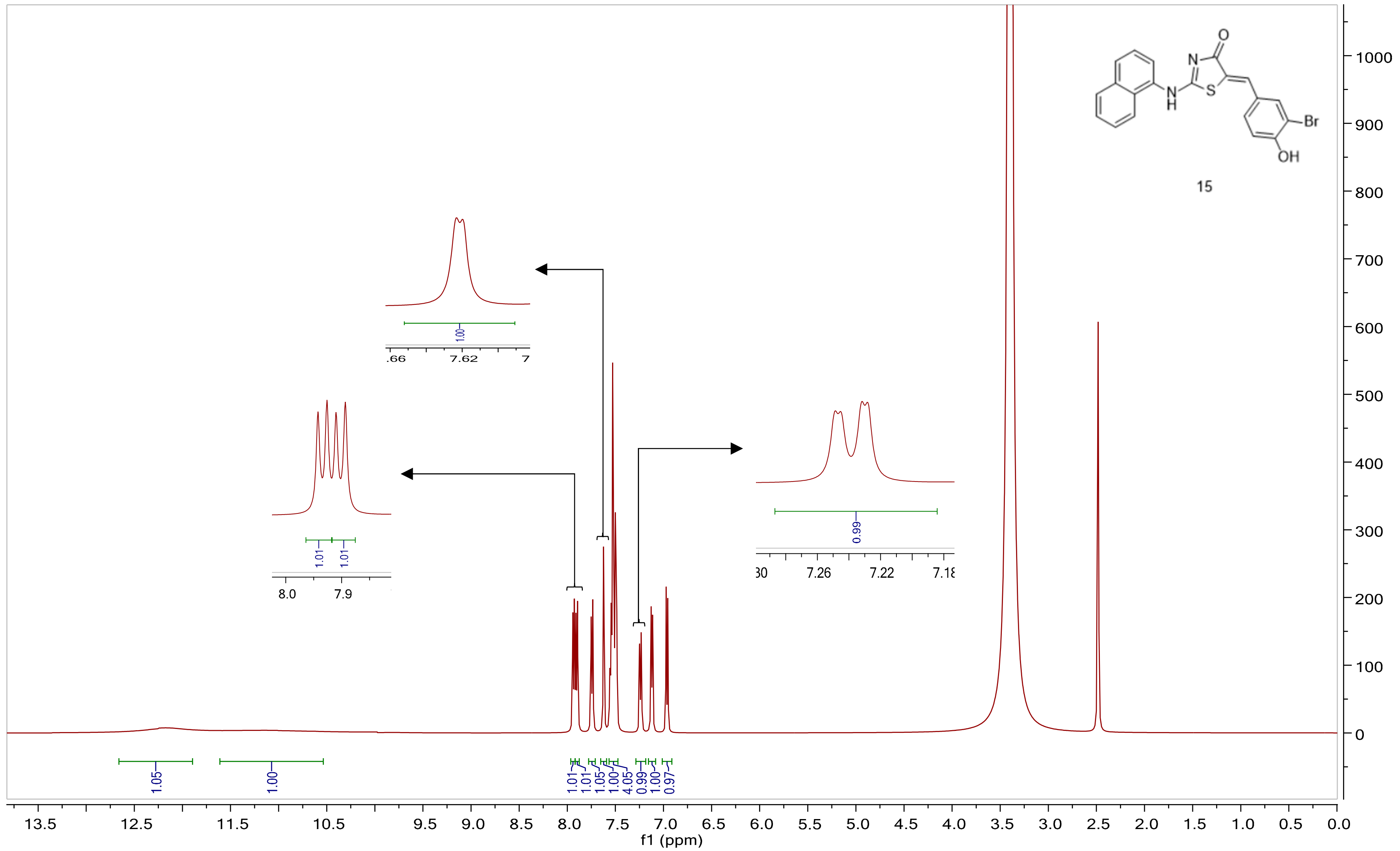

Figure S50. ^{1}H NMR spectrum of analog **15**

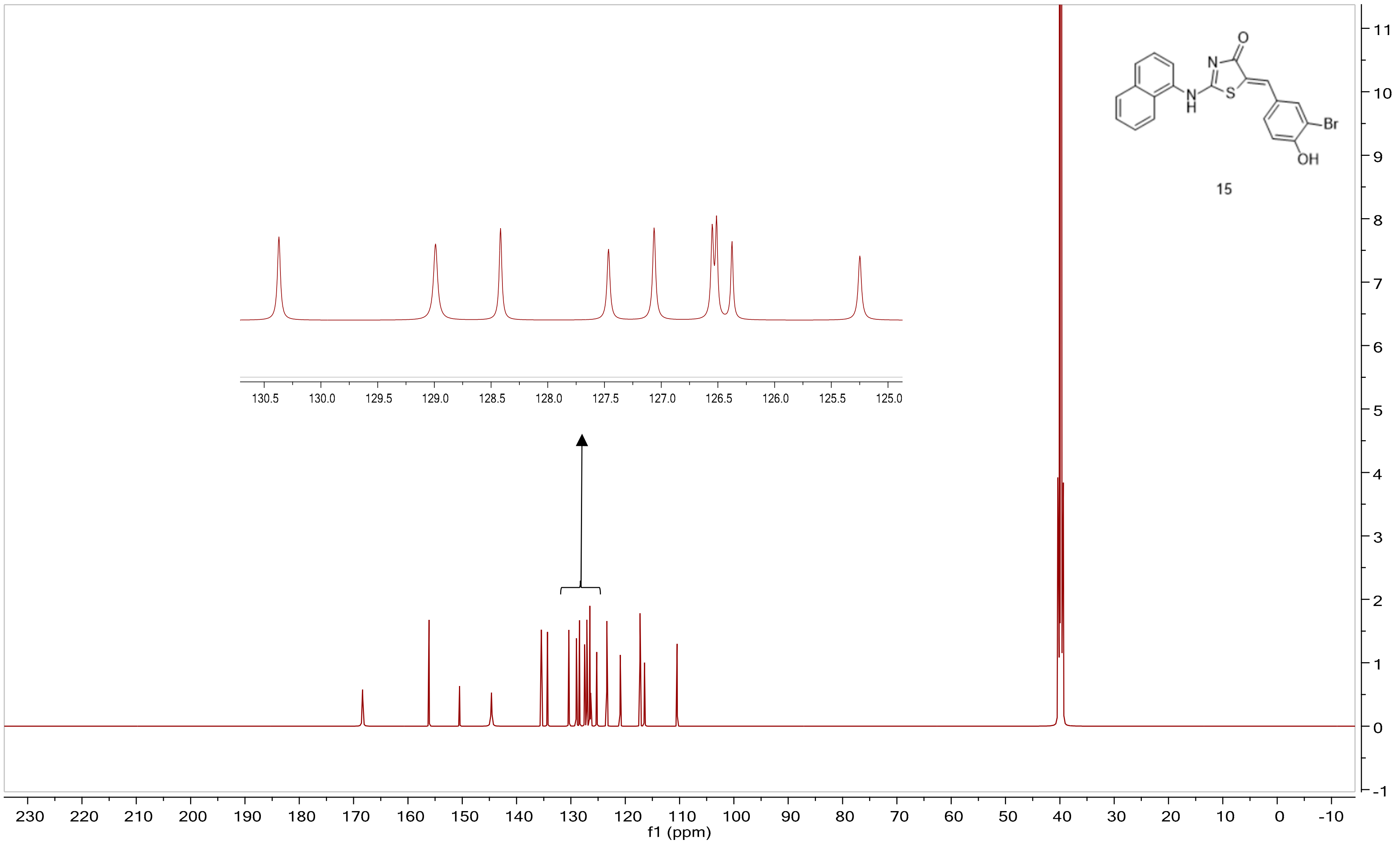

Figure S51. ^{13}C NMR spectrum of analog **15**

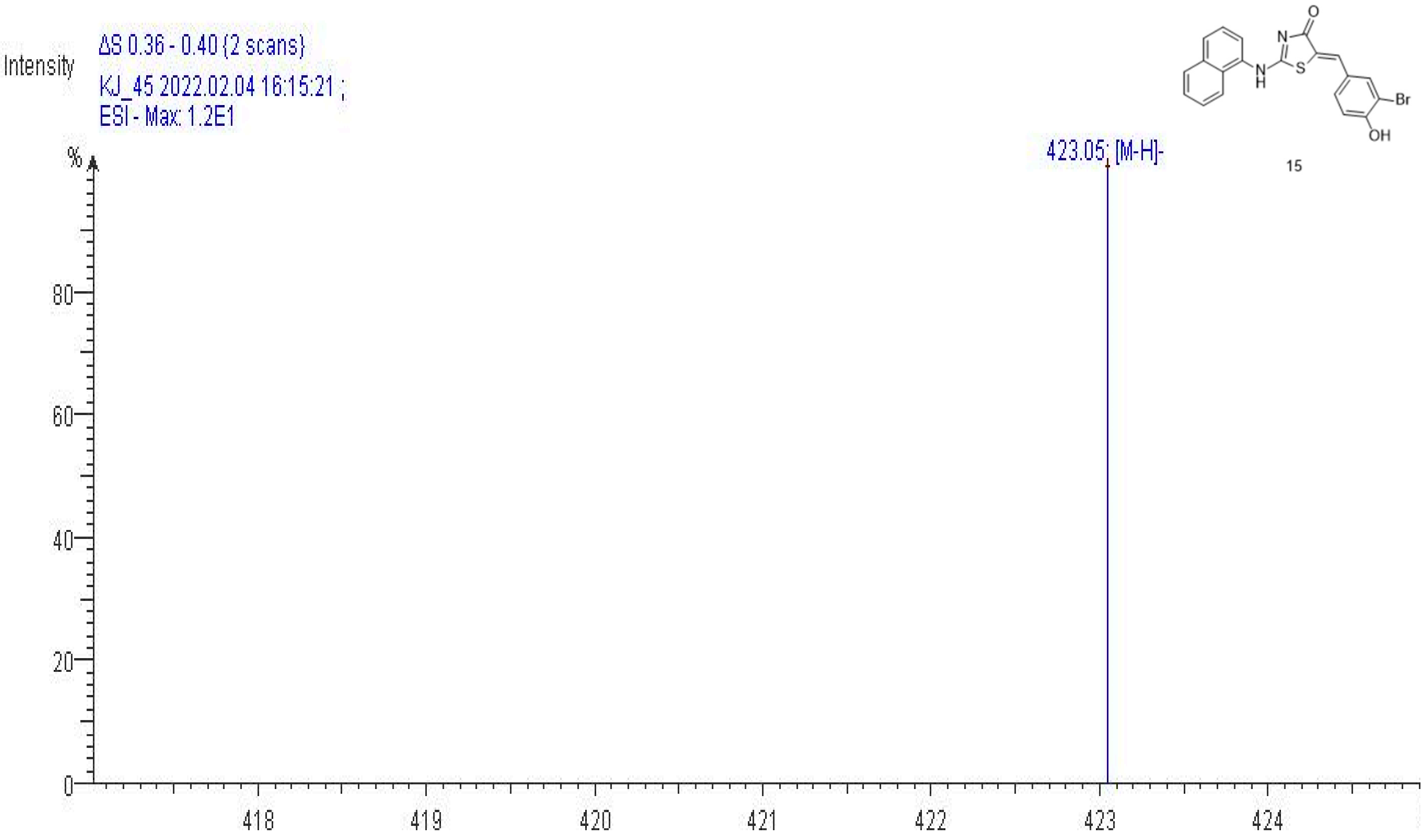

Figure S52. LRMS (ESI–) spectrum of analog **15**

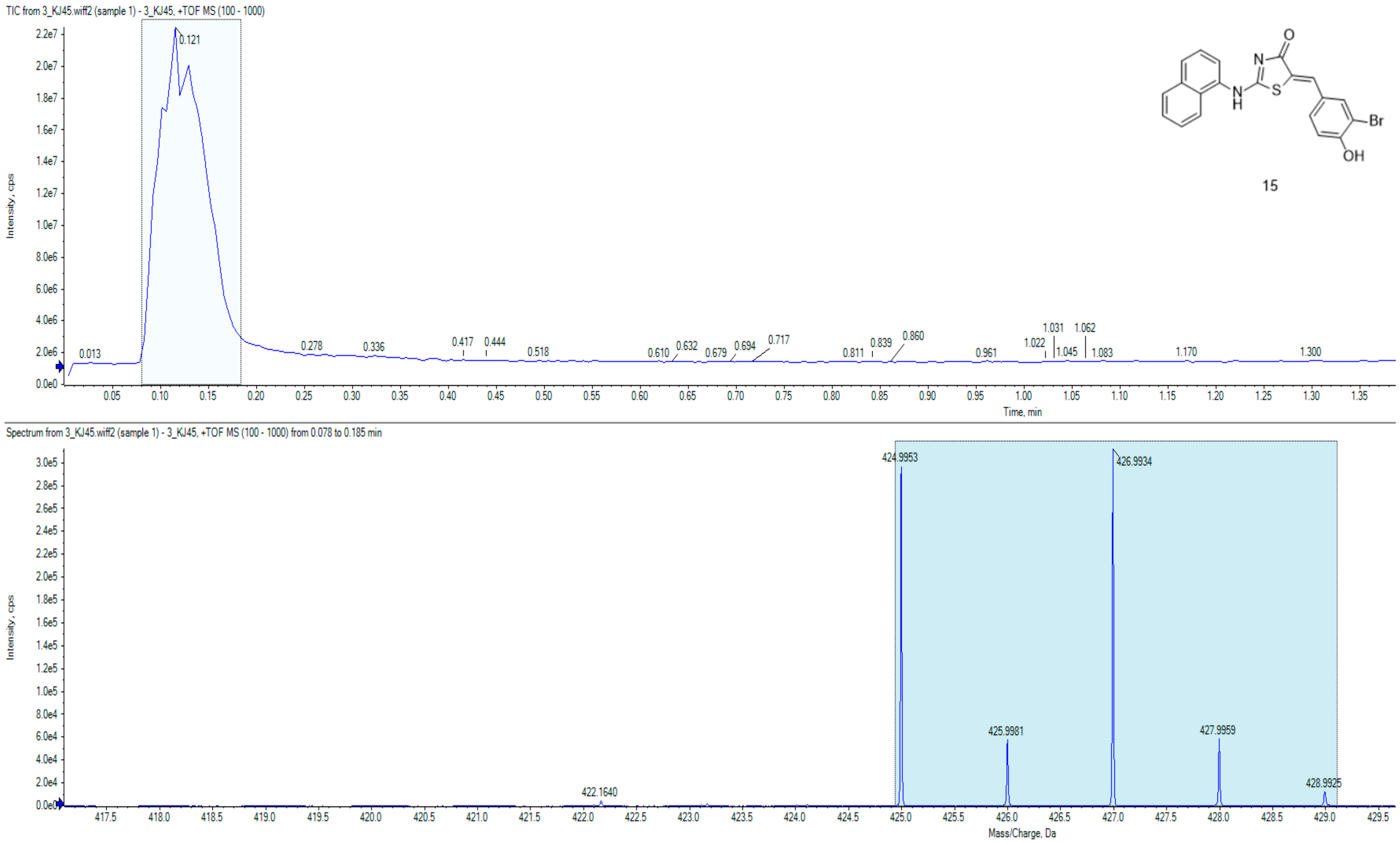

Figure S53-1. HRMS (ESI+) spectrum of analog **15**

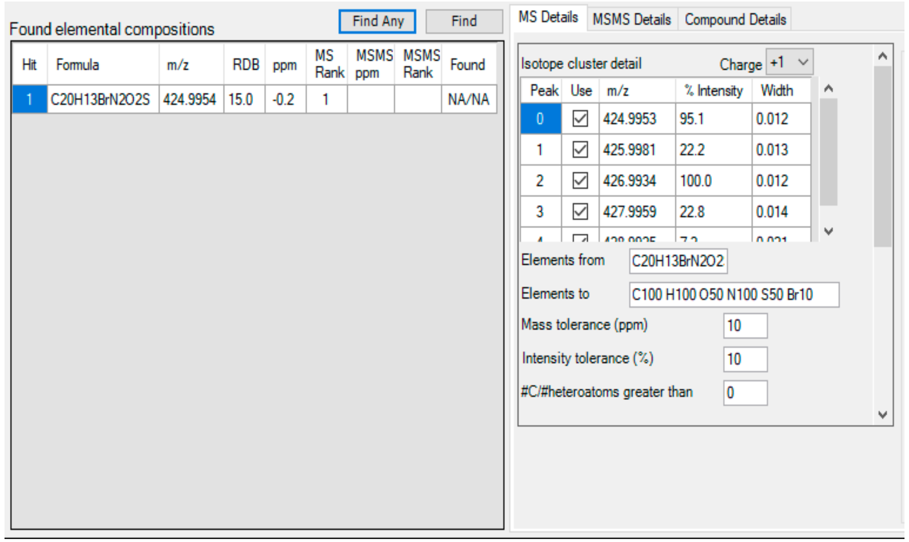

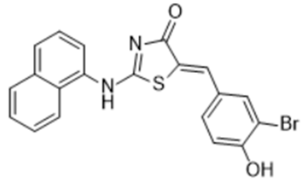

15

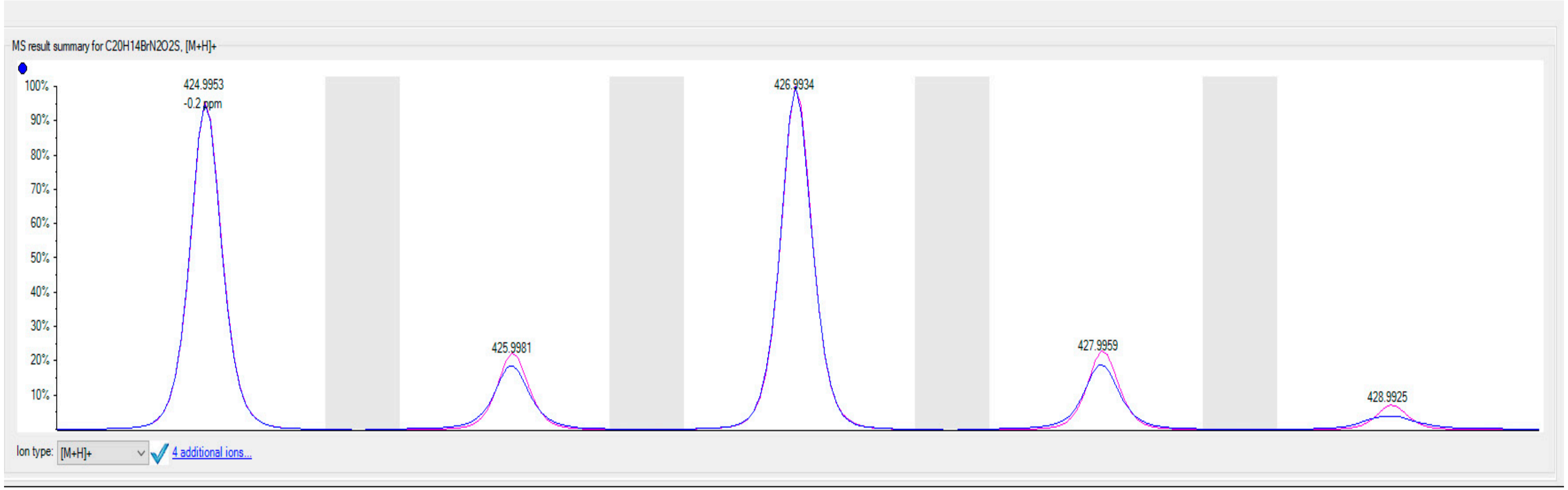

Figure S53-2. HRMS (ESI+) spectrum of analog **15**

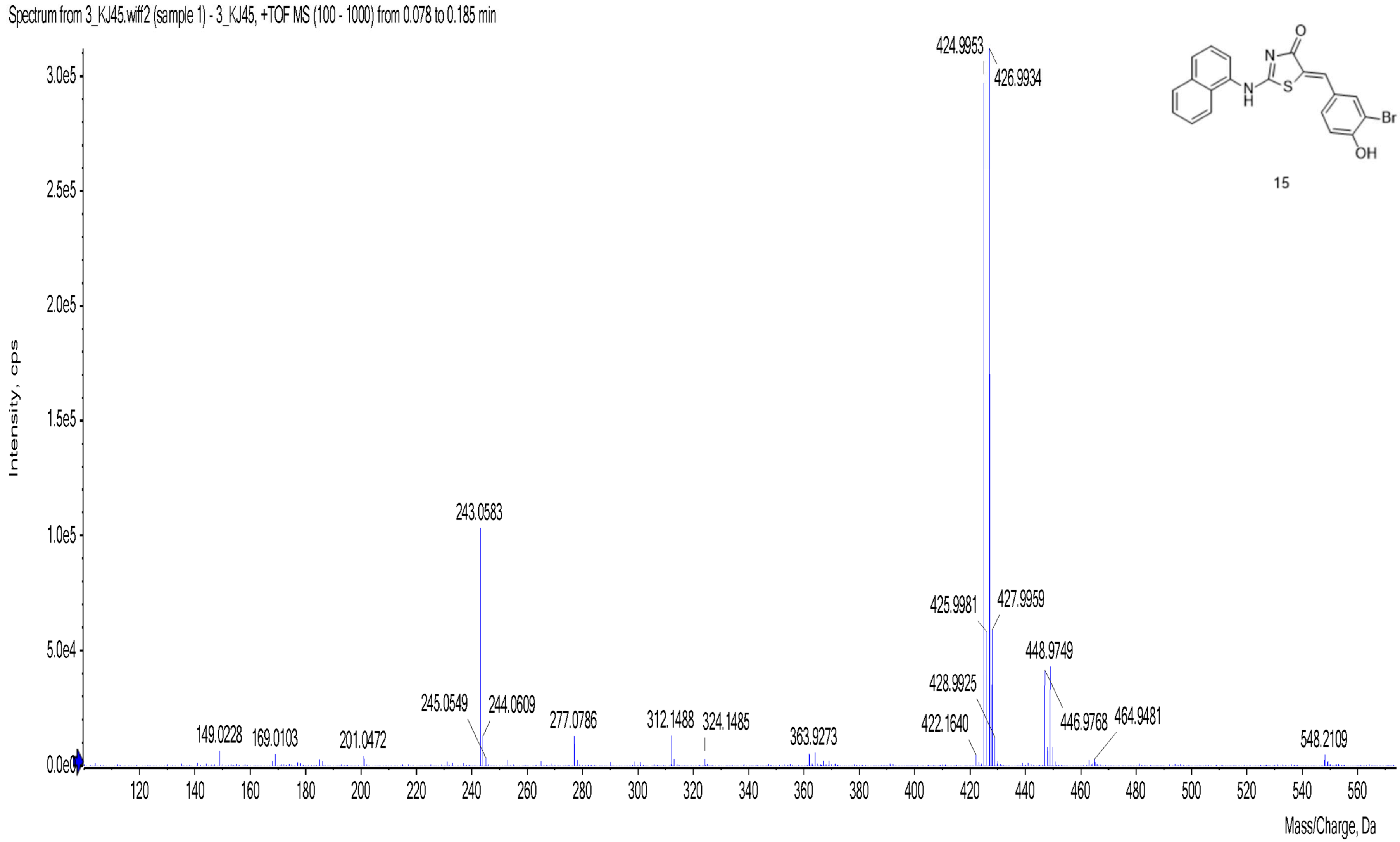

Figure S53-3. HRMS (ESI+) spectrum of analog **15**

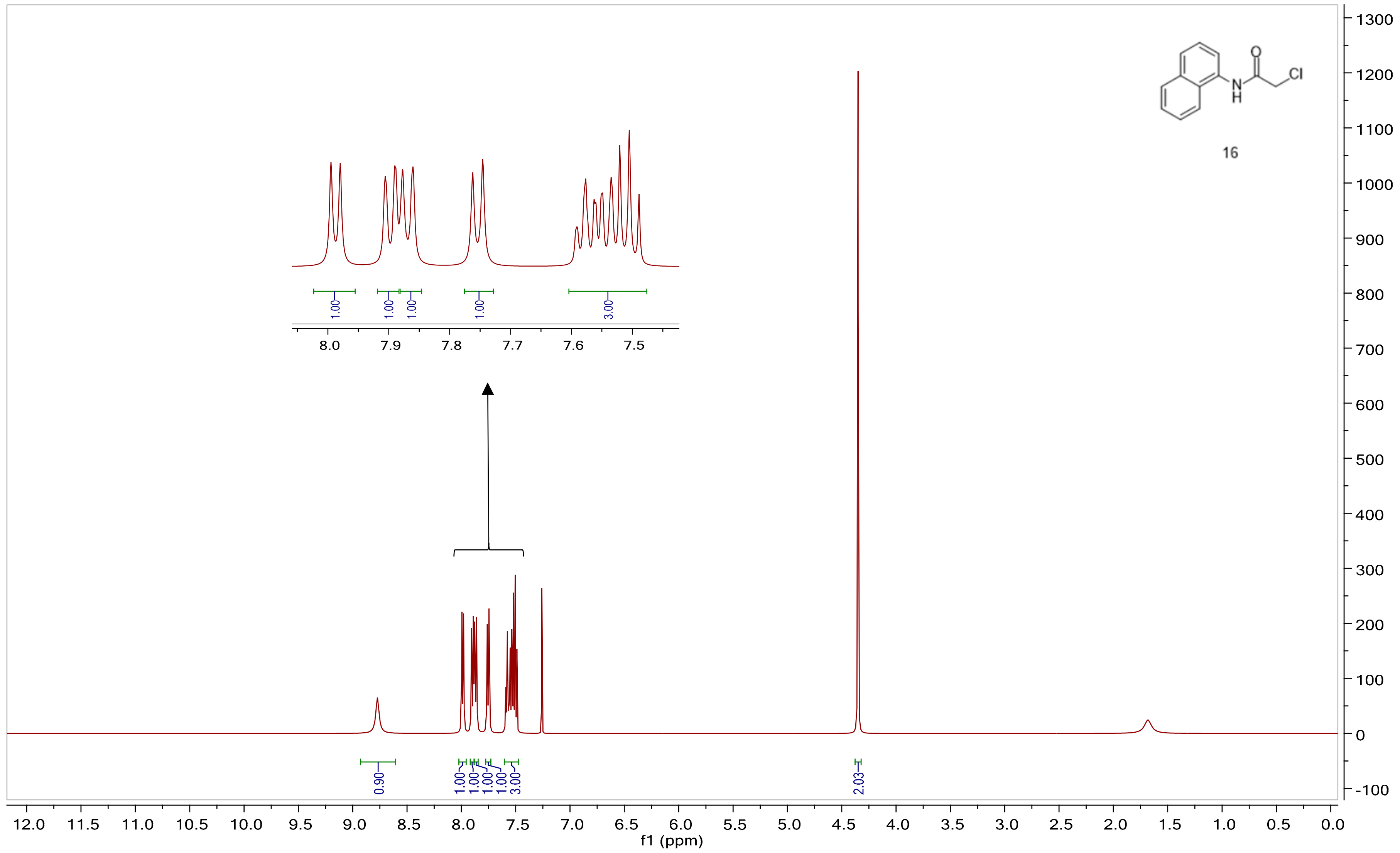

Figure S54. ^{1}H NMR spectrum of analog **16**

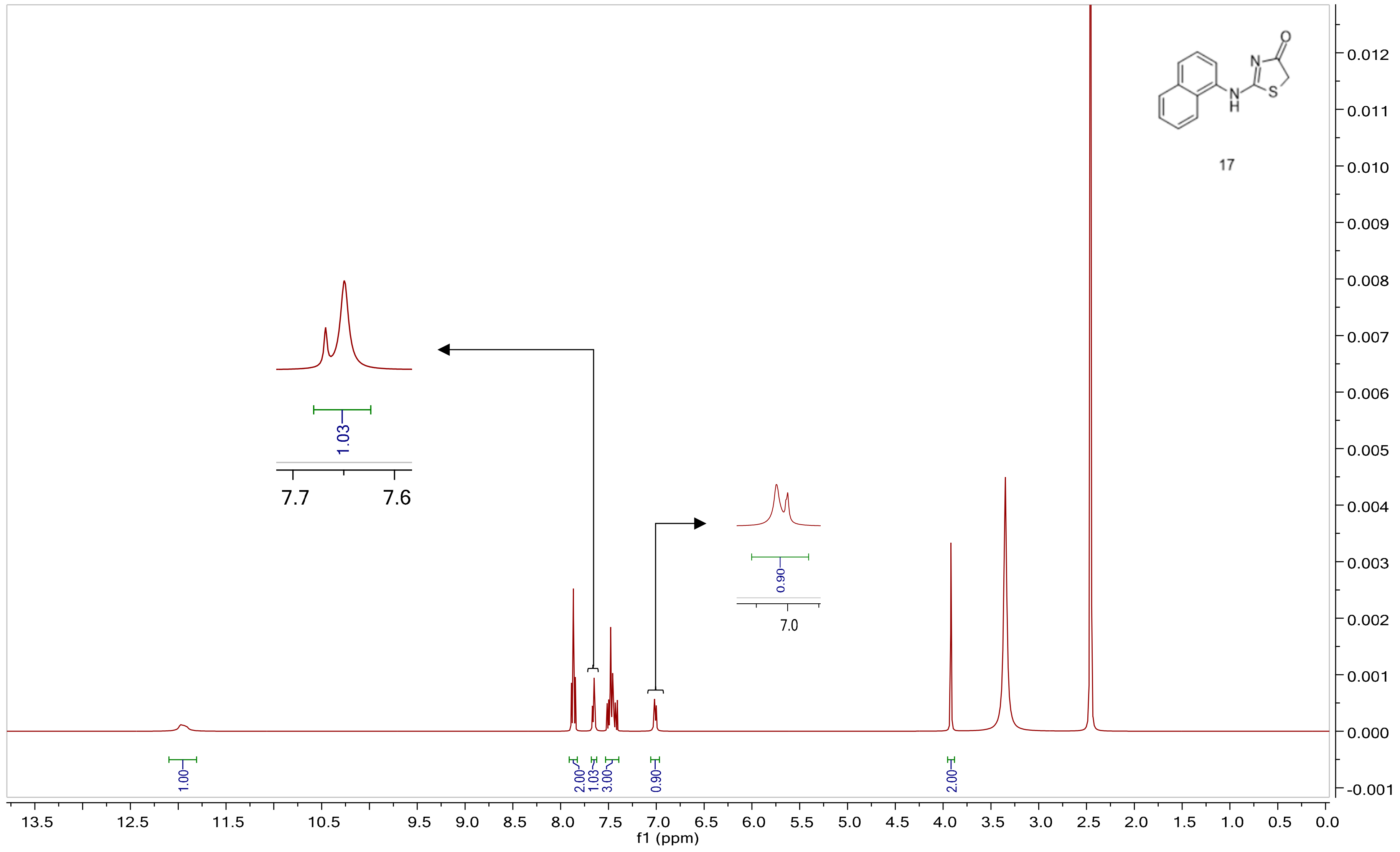

Figure S55. ¹H NMR spectrum of analog **17**

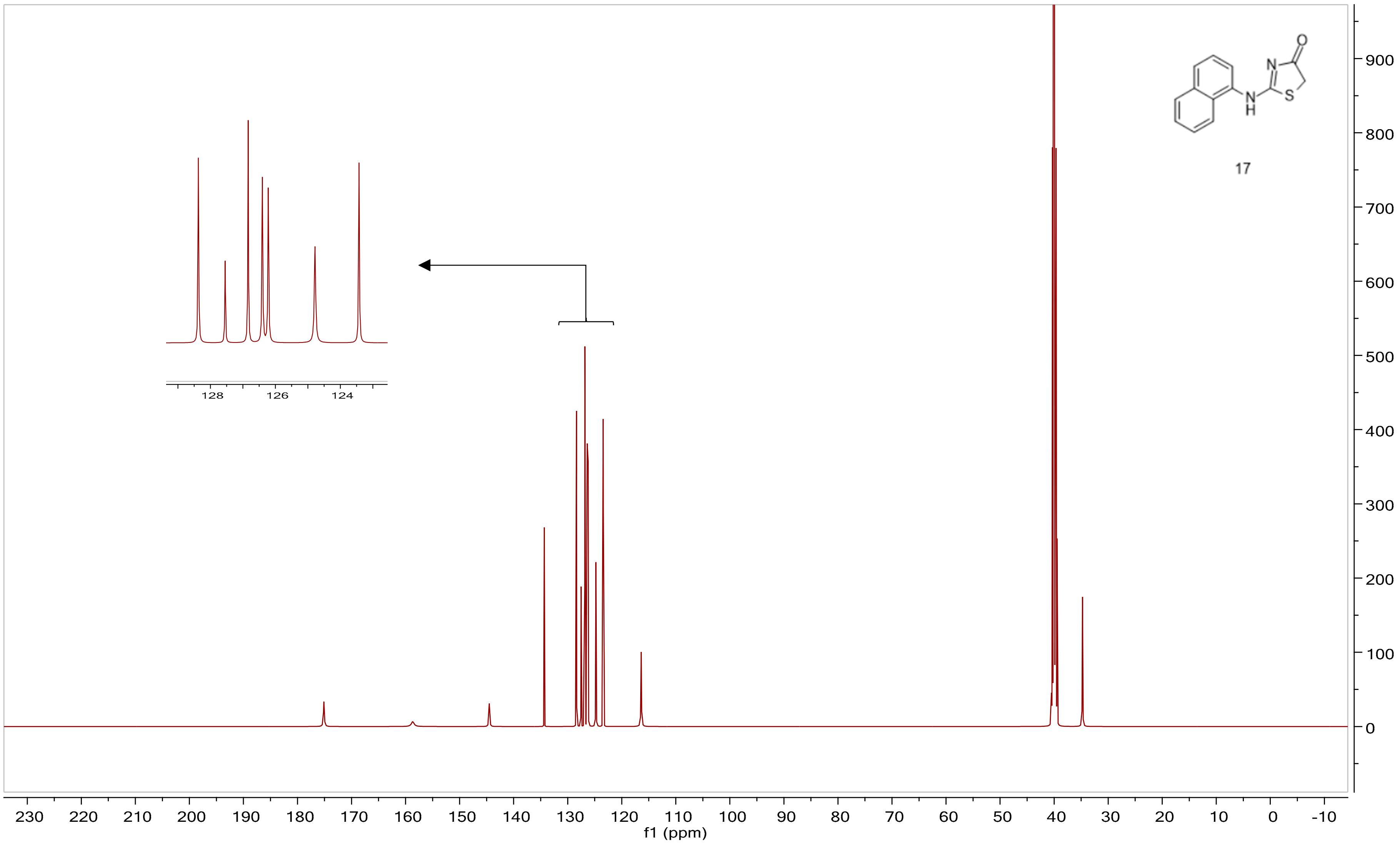

Figure S56. ^{13}C NMR spectrum of analog **17**

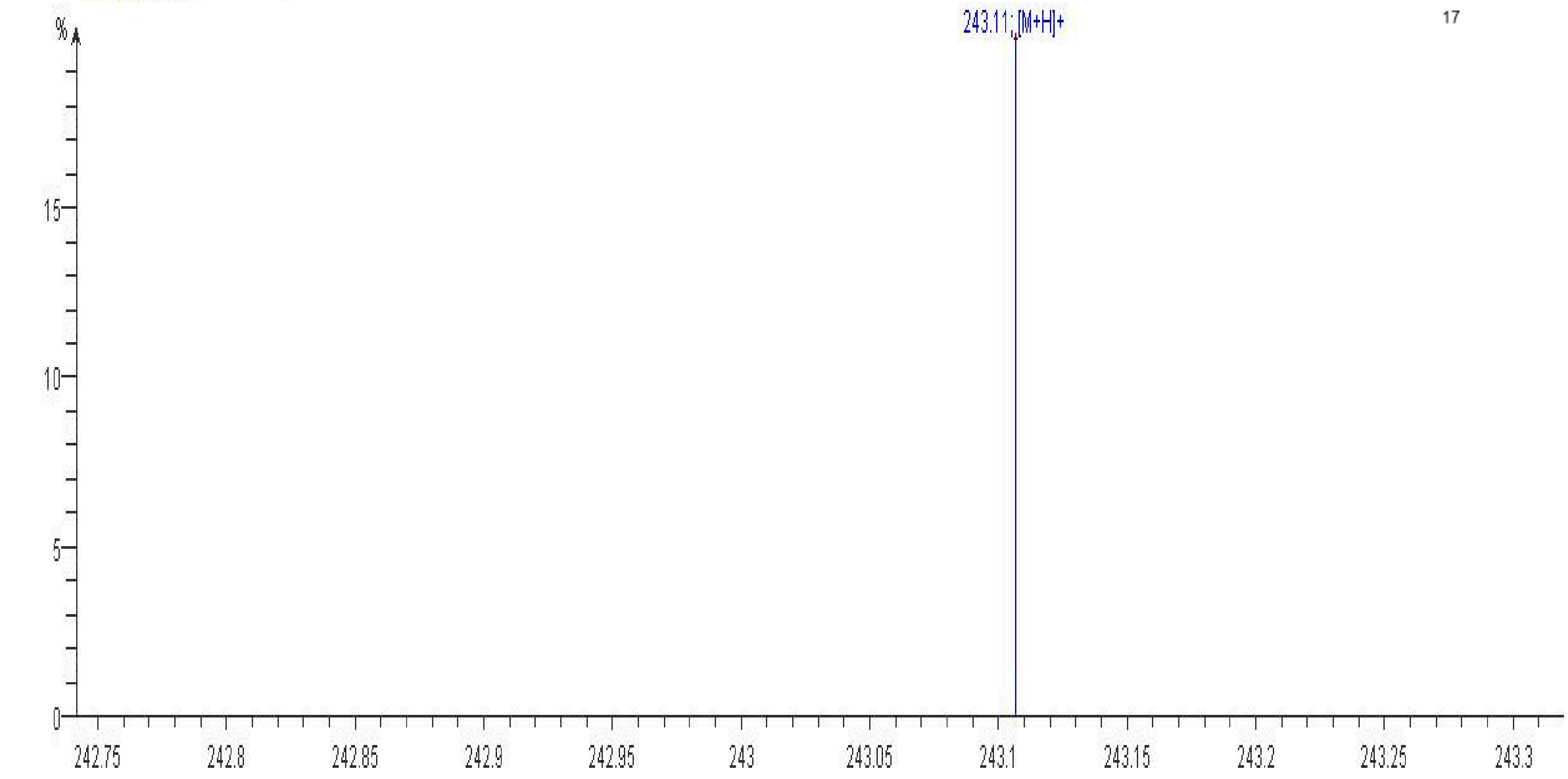

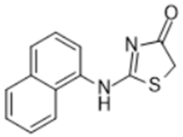

17

Figure S57. LRMS (ESI+) spectrum of analog **17**

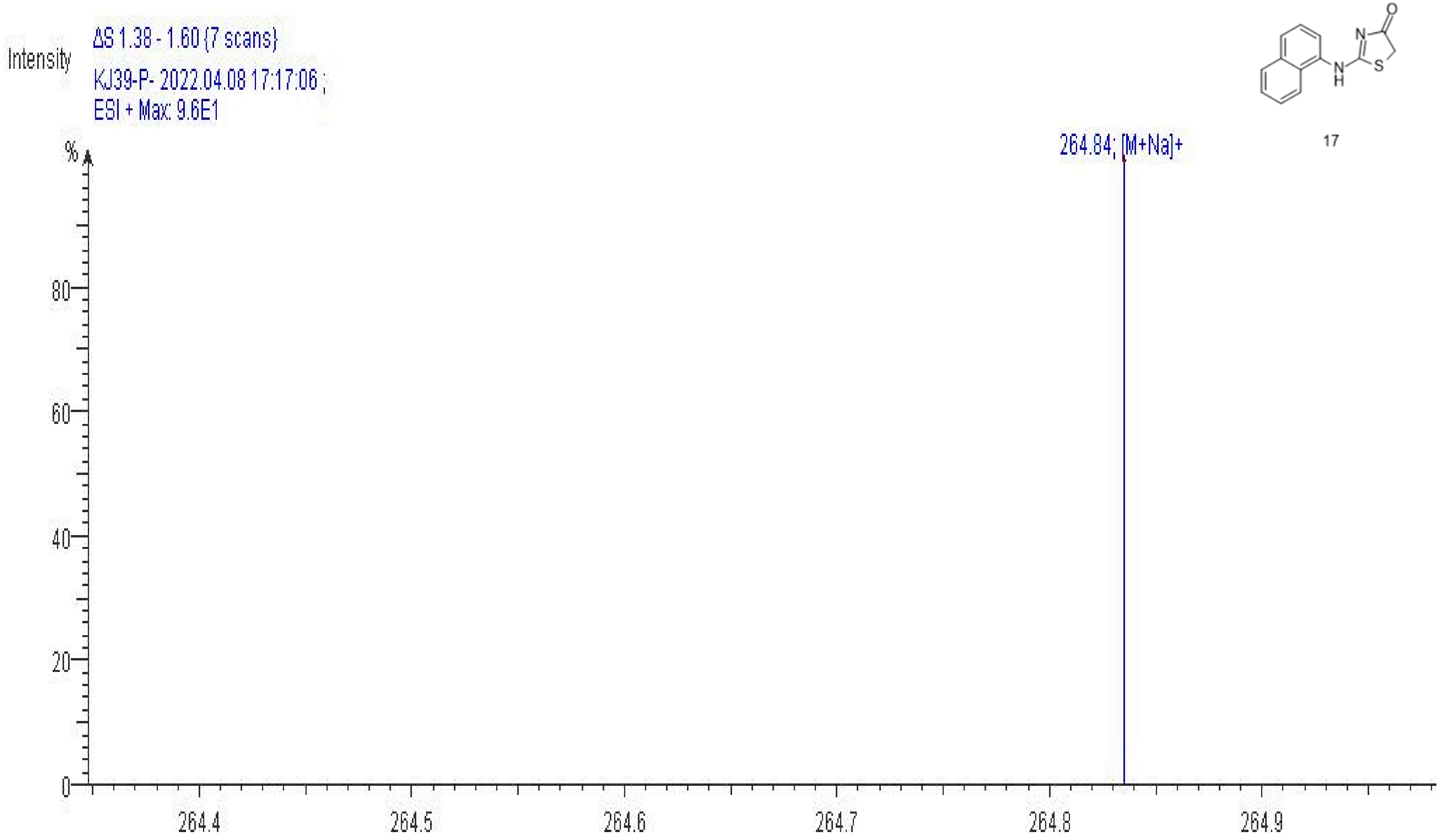

17

Figure S58. LRMS (ESI+) spectrum of analog **17**

Figure S59. In silico 2D results obtained from docking simulation of ligands (1–9 and 11–15) with mushroom tyrosinase (PDB ID: 2Y9X)

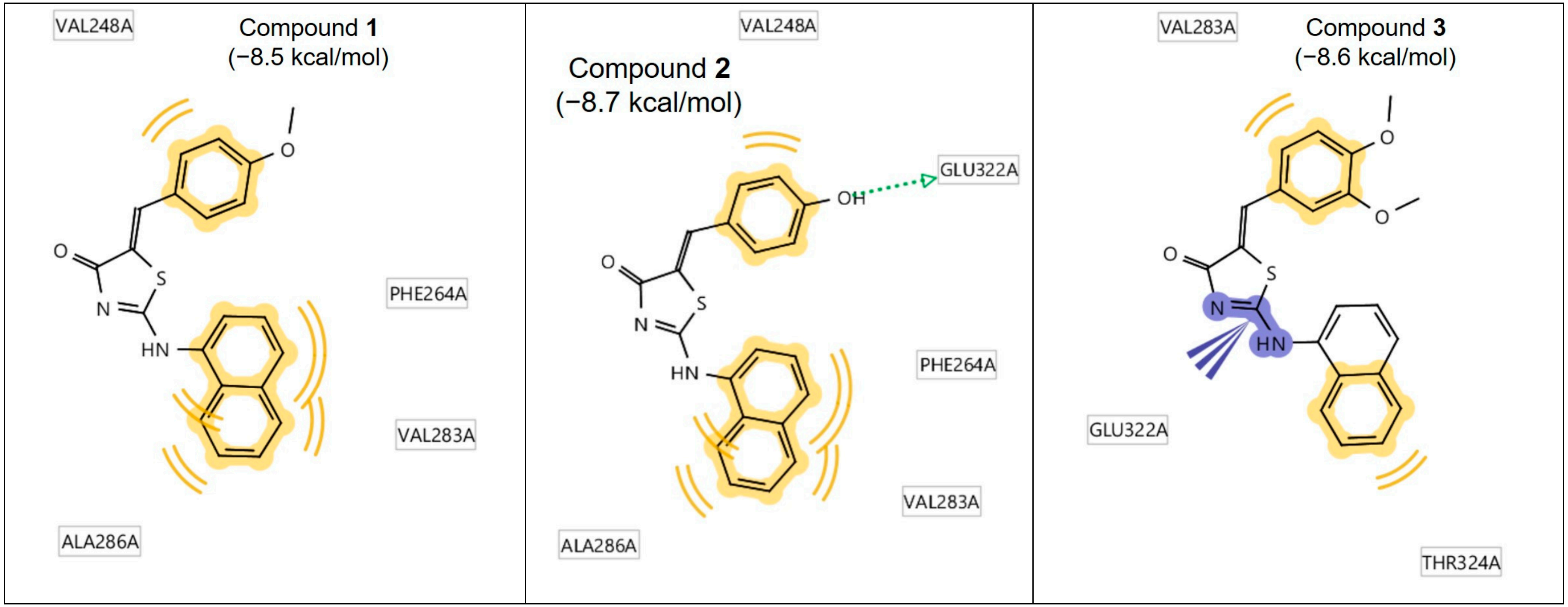

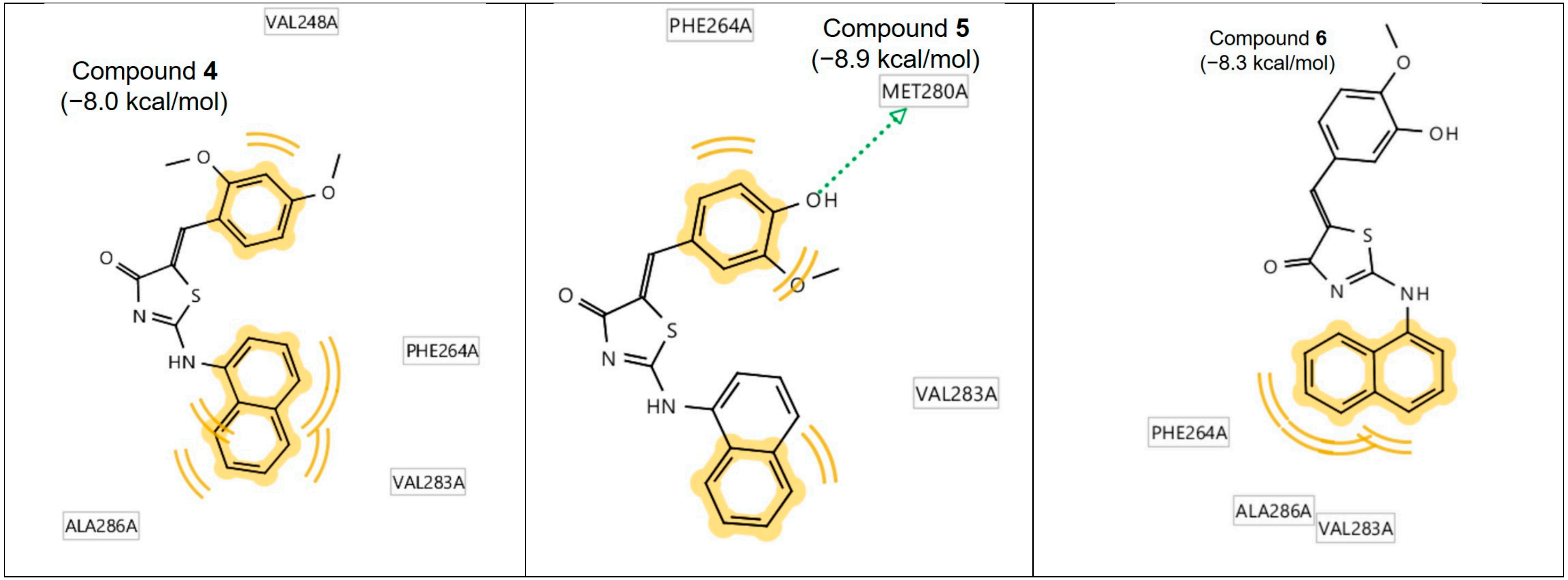
VAL248A
Compound 4
(−8.0 kcal/mol)
O
O
O
S
N
HN
PHE264A
VAL283A
ALA286A
PHE264A
Compound 5
(−8.9 kcal/mol)
MET280A
OH
O
O
S
N
HN
VAL283A
Compound 6
(−8.3 kcal/mol)
O
OH
S
O
N
NH
PHE264A
ALA286A
VAL283A

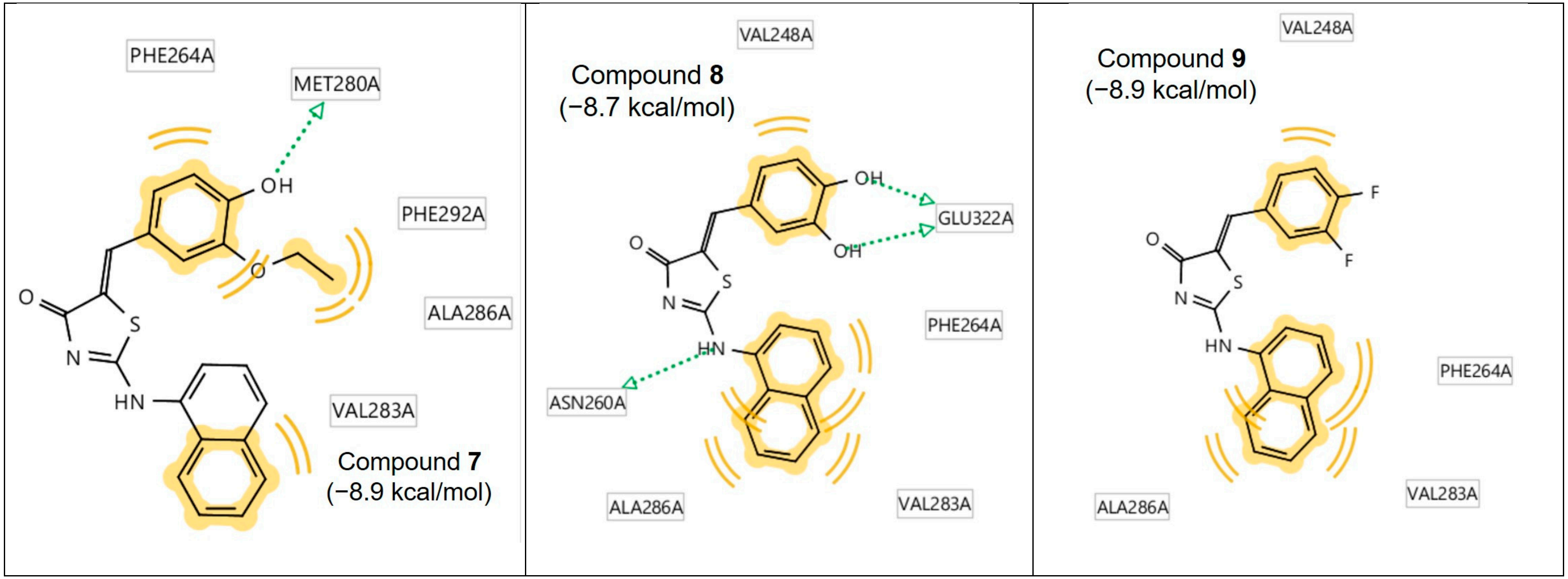
PHE264A
MET280A
OH
PHE292A
O
ALA286A
O
S
N
HN
VAL283A
Compound **7**
(−8.9 kcal/mol)
VAL248A
Compound **8**
(−8.7 kcal/mol)
OH
GLU322A
OH
O
S
N
HN
PHE264A
ASN260A
ALA286A
VAL283A
VAL248A
Compound **9**
(−8.9 kcal/mol)
F
F
O
S
N
HN
PHE264A
ALA286A
VAL283A

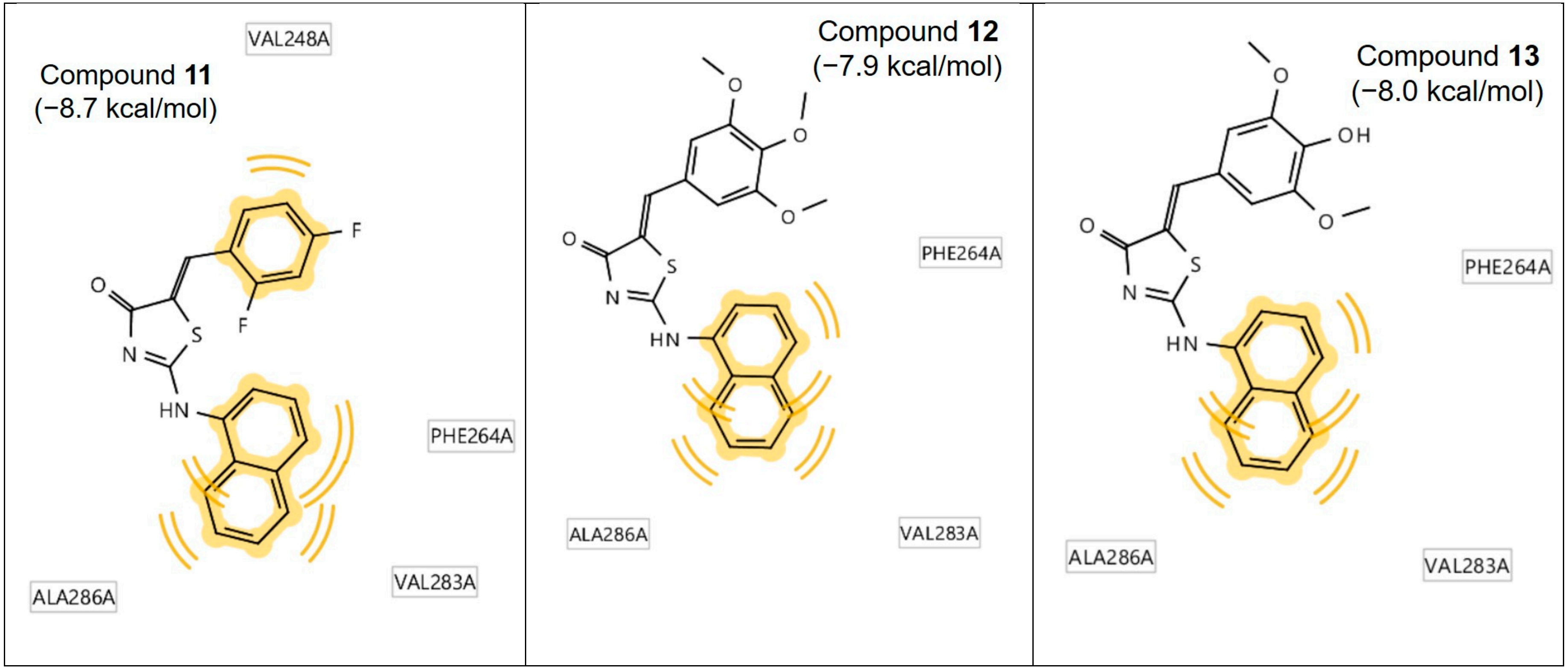

Compound 11
(−8.7 kcal/mol)
VAL248A
PHE264A
ALA286A
VAL283A
Compound 12
(−7.9 kcal/mol)
PHE264A
ALA286A
VAL283A
Compound 13
(−8.0 kcal/mol)
PHE264A
ALA286A
VAL283A

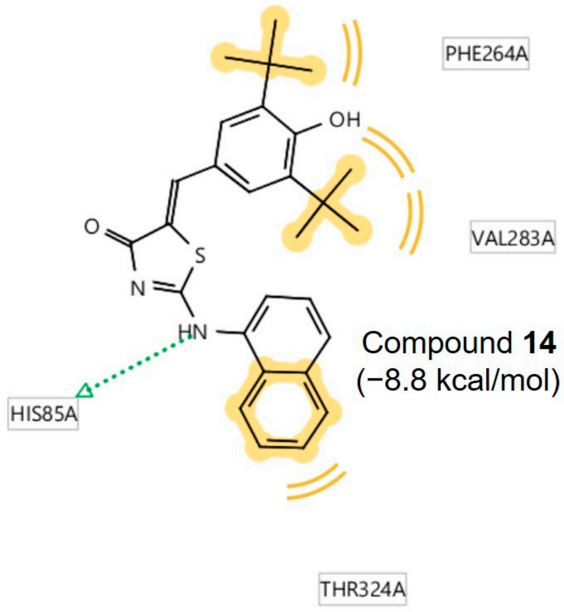<br>Compound **14**<br>(−8.8 kcal/mol)		
